# Impact of close interpersonal contact on COVID-19 incidence: evidence from one year of mobile device data

**DOI:** 10.1101/2021.03.10.21253282

**Published:** 2021-03-12

**Authors:** Forrest W. Crawford, Sydney A. Jones, Matthew Cartter, Samantha G. Dean, Joshua L. Warren, Zehang Richard Li, Jacqueline Barbieri, Jared Campbell, Patrick Kenney, Thomas Valleau, Olga Morozova

**Affiliations:** 1 Department of Biostatistics, Yale School of Public Health, New Haven, CT, USA; 2 Department of Statistics & Data Science, Yale University, New Haven, CT, USA; 3 Department of Ecology & Evolutionary Biology, Yale University, New Haven, CT, USA; 4 Yale School of Management, New Haven, CT, USA; 5 Epidemic Intelligence Service, Centers for Disease Control & Prevention, Atlanta, GA, USA; 6 Infectious Diseases Section, Connecticut Department of Public Health, New Haven, CT, USA; 7 Department of Statistics, University of California, Santa Cruz, Santa Cruz, CA, USA; 8 Whitespace Solutions, Ltd, Alexandria, VA, USA; 9 Program in Public Health and Department of Family, Population and Preventive Medicine, Stony Brook University, NY, USA

**Keywords:** SARS-CoV-2, infectious disease, transmission, mobility

## Abstract

Close contact between people is the primary route for transmission of SARS-CoV-2, the virus that causes coronavirus disease 2019 (COVID-19). We sought to quantify interpersonal contact at the population-level by using anonymized mobile device geolocation data. We computed the frequency of contact (within six feet) between people in Connecticut during February 2020 – January 2021. Then we aggregated counts of contact events by area of residence to obtain an estimate of the total intensity of interpersonal contact experienced by residents of each town for each day. When incorporated into a susceptible-exposed-infective-removed (SEIR) model of COVID-19 transmission, the contact rate accurately predicted COVID-19 cases in Connecticut towns during the timespan. The pattern of contact rate in Connecticut explains the large initial wave of infections during March–April, the subsequent drop in cases during June–August, local outbreaks during August–September, broad statewide resurgence during September–December, and decline in January 2021. Contact rate data can help guide public health messaging campaigns to encourage social distancing and in the allocation of testing resources to detect or prevent emerging local outbreaks more quickly than traditional case investigation.

## One sentence summary

Close interpersonal contact measured using mobile device location data explains dynamics of COVID-19 transmission in Connecticut during the first year of the pandemic.

## Introduction

Close contact between people is the primary route for transmission of the novel severe acute respiratory syndrome coronavirus 2 (SARS-CoV-2), the virus that causes coronavirus disease (COVID-19) [1]. Social distancing guidelines published by the United States (U.S.) Centers for Disease Control and Prevention (CDC) recommend that people stay at least six feet away from others to avoid transmission via direct contact or exposure to respiratory droplets [2]. Throughout the world, non-pharmaceutical interventions, including social distancing guidelines and stay-at-home orders, have been employed to encourage the physical separation of people and reduce the risk of COVID-19 transmission via close contact [3–6]. U.S. states with the lowest levels of self-reported social distancing behavior are experiencing most severe COVID-19 outbreaks [7].

While individual-level compliance with social distancing guidelines can be difficult to measure, researchers have proposed population-level mobility metrics based on mobile device geolocation data as a proxy measure for physical distancing and movement patterns during the COVID-19 pandemic [8–12]. Investigators have characterized geographic and temporal changes in mobility metrics following non-pharmaceutical interventions like social distancing guidelines and stay-at-home mandates during the COVID-19 pandemic [11, 13–22]. Researchers have also studied the association between mobility metrics and COVID-19 cases or other proxy measures of transmission [11, 23–34]. Most mobility metrics measure aggregated movement patterns of individual mobile devices: time spent away from home, distance traveled, or density of devices appearing in an area during a given time interval. CDC reports mobility metrics from Google, Safegraph, and Cuebiq [35]. Some mobility metrics measure spatial relationships among individual devices. Klein et al. [12] measure “colocation” events, in which reported locations of two devices lie within a roughly 60 square foot spatial grid cell. Couture et al. [16] compute a “device exposure index” that measures the colocation of devices within a sample of preselected venues like restaurants or retail establishments. Chang et al. [30] use colocation matrices from Facebook [36] that measure the probability that devices from different geographic areas appear in the same 600-meter square region for five minutes, aggregated by week. Morley et al. [37] use the “human encounters” metric from Unacast [38, 39] that measures the frequency of two devices being within 50 meters of each other for an hour or less. Finally, Cuebiq offers a contact index measuring when two or more devices are within 50 feet of each other within five minutes [40].

Existing mobility metrics might not capture simultaneous colocation of devices, do not measure contact within a two-meter distance associated with highest transmission risk (via direct contact or exposure to respiratory droplets), and might not take intrinsic mobile device spatial location error (horizontal uncertainty) into account. A better measure of contact events – the primary behavioral risk factor for transmission – could help explain historical patterns of transmission, assist policymakers in targeting interventions and messaging campaigns to encourage social distancing, guide public health response measures such as enhanced testing and contact tracing, and provide early warning to detect and prevent emerging outbreaks. By using highly detailed mobile device geolocation data and a novel probabilistic method for assessing close proximity, we sought to quantify total intensity of close interpersonal contact (within six feet) at the population-level (contact rate) and to use contact rate to explain patterns of COVID-19 incidence and predict emergence of new COVID-19 cases in the state of Connecticut, U.S. during February 1, 2020 – January 31, 2021.

## Setting: Connecticut

Connecticut (population 3.565 million), like other states in the northeastern U.S., experienced a strong initial wave of COVID-19 infections during March–April 2020 following outbreaks in the New York City area [41, 42]. On March 17, Connecticut Governor Ned Lamont closed schools [43–46], and issued a statewide “Stay Safe, Stay Home” mandate to take effect on March 23, 2020 [47]. Governor Lamont’s executive order recommended that nonessential businesses cease all in-person functions, closed in-person dining at restaurants, and cancelled all in-person community gatherings. The mandate excluded healthcare, food service, law enforcement, and other essential services.

As case counts declined, Connecticut followed a gradual reopening plan designed to resume economic activity while minimizing the risk of transmission via close contact between people. On May 20, the state entered Phase 1, permitting the following to open at 50% capacity with social distancing: hair salons and barbershops, outdoor zoos and museums, outdoor dining, outdoor recreation, retail shopping, university research, and offices, although work from home was strongly encouraged [48]. On June 17, Phase 2 began, permitting indoor religious services at 25% capacity and capped at 100 people, outdoor religious services capped at 150 people, and opening indoor dining, hair salons, personal service businesses, and libraries at 50% capacity [49]. A serology study to measure prevalence of SARS-CoV-2-specific IgG antibodies was conducted among adult Connecticut residents residing in non-congregate settings during June–July [50]. The study estimated a seroprevalence of 4.0% (90% confidence interval 2.0%–6.0%). Participants in the study reported their risk mitigation behaviors: 73% avoided public places, 75% avoided gatherings of families or friends, and 97% wore a mask at least some of the time. In July, Governor Lamont delayed the state’s planned summer move to Phase 3 – which would have loosened occupancy restrictions on bars and restaurants – because of surges in transmission occurring elsewhere in the U.S. [51].

Connecticut experienced low COVID-19 incidence and declining hospitalization during June–August, but in August a major outbreak occurred in Danbury, a town in the western part of the state [52]. During August–September, in-person education resumed at many colleges, universities, and primary/secondary schools in Connecticut. By mid-September, the state was facing a broad resurgence of COVID-19 transmission. On September 17, the Connecticut Department of Public Health reported that the number of new cases per week for the previous 4 weeks was 62% higher than the average number of new cases per week in July and early August [53]. These signs of resurgence were initially concentrated in southeastern Connecticut, where few COVID-19 cases were identified during the initial spring wave in March–April [54, 55].

Public health officials identified travel, social gatherings, workplaces, churches, universities, and recreational sports as contributing to transmission [56]. Nevertheless, on October 8, the state began Phase 3 reopening, permitting 50% capacity in houses of worship capped at 200 people, uncapped outdoor religious gatherings with social distancing, and opening indoor dining, hair salons, personal service businesses, and libraries at 75% capacity [49]. On November 6, as COVID-19 case counts continued to increase, Connecticut reverted to “Phase 2.1”, reducing indoor restaurant seating, indoor and outdoor event capacity, and placing caps on attendance [49]. Case counts increased through December and began to decline in January 2021. Overall, Connecticut residents complied with state guidelines and mandates to reduce close contact. In a survey of risk mitigation behaviors throughout the U.S., Lazer et al. [7] reported that Connecticut ranked 9th among U.S. states in self-reported social distancing during fall 2020, and 6th in self-reported mask wearing. But case counts indicate that Connecticut experienced widely varying temporal and geographic dynamics of COVID-19 incidence over the course of the pandemic.

## Computing contacts from anonymized mobile device geolocation data

We obtained anonymized mobile device geolocation data for a sample of devices in Connecticut from X-Mode. During May 1, 2020 through January 31, 2021, we observed a total of 788,842 unique (anonymized) device IDs, representing roughly 22% of the approximately 3.565 million residents of Connecticut (though some of those devices may have belonged to people residing elsewhere). An average of 141,617 unique devices were observed per day. For each week, an average of 80.5% of device IDs from the prior week were present in the data. Devices might not be present in the dataset if the user turns off the device or does not interact with applications that report location data. Using device geolocation records consisting of anonymized device IDs, GPS coordinates, date/time stamps, and GPS location error estimates (horizontal uncertainty), we calculated the location in which each device had the most location records and designated that area as the device’s primary dwell location (i.e., town of residence of device owner).

**Figure 1:**
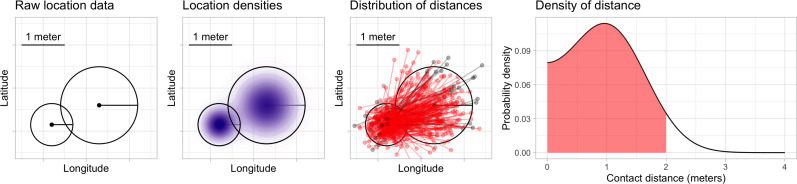
Schematic illustration of contact probability calculation. From left to right: raw locations, including horizontal uncertainty estimates, for two mobile devices are transformed into approximate location probability densities. The distribution of distances from points drawn randomly from these densities is computed. Sampled distances are shown here for illustrative purposes in red (when sampled device locations are within six feet apart) and gray (when sampled locations are more than six feet apart); in our implementation, the distribution of these distances is computed analytically. The shaded area under the density is the probability that the devices are within six feet.

A contact event was computed by using a probabilistic algorithm that computes the likelihood of simultaneous 2-meter proximity between pairs of devices across geographic areas. For each device, we identify sets of records where devices were in spatial proximity to one another and stationary. A limitation of mobile device gelocation data is that it is not possible to precisely quantify the duration a device is stationary because device locations are collected asynchronously and irregularly over time. For each potential contact event, we compute the probability that the two device locations are within six feet by assuming that the reported device locations arise from a two-dimensional Gaussian probability distribution whose variance is computed by using the horizontal uncertainty measure, and correct the distance to account for the curvature of the earth. Figure 1 shows a schematic illustration of the contact event probability calculation.

We define the “contact rate” as the total number of contact events per day among observed devices at the town level; the contact rate is computed by summing daily contact probabilities for each device and assigning that sum to the device primary dwell location. A detailed description of the mobile device geolocation data, computation of the probability of contact, spatial aggregation of the contact probabilities to estimate contact rate, and coverage of mobile devices across Connecticut is given in the Supplementary Materials.

The mobile device geolocation and COVID-19 case data contain no individually identifying meta-data and were aggregated by day and town. This work was approved by the Yale University institutional review board. This work was also reviewed by CDC and was conducted consistently with applicable federal law and CDC policy ^1^.

## Statewide contact trends

**Figure 2:**
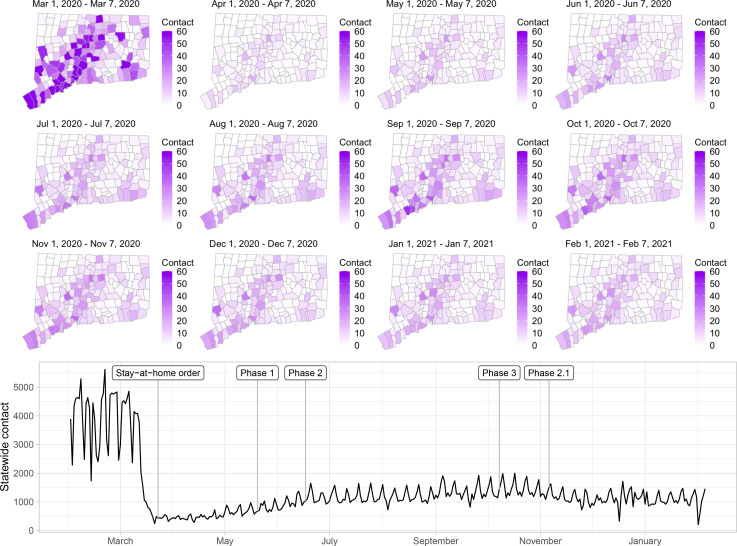
Estimated contact rate among mobile devices in our dataset in Connecticut from February 2020 to February 2021. At top, maps show the number of contacts in Connecticut’s 169 towns per day during weeks beginning on the first of each month. Darker colors indicate higher contact. At bottom, statewide contact shows the daily frequency of close contact within six feet between distinct devices in our dataset. Governor Ned Lamont’s stay-at-home order and reopening phases 1, 2, 3, and 2.1 indicated. The state reverted to the more restrictive “Phase 2.1” in response to rising case counts in November.

Figure 2 shows the contact rate by town in Connecticut during February 1 – January 31, 2021. Maps show the weekly average of daily contact rate by town, where darker colors in maps indicate higher contact rate. The daily contact rate is shown in the plot below. The statewide contact rate dropped dramatically in March, about one week before Governor Lamont issued the statewide stay-at-home mandate on March 23 [47]. News of surging COVID-19 hospitalization and responses in the New York area [57, 58], closure of public schools, and anticipation of a possible stay-at-home order might have played a role in reducing contact before the mandate was announced. After staying low during most of April, the contact rate began to rise slowly throughout the state during June–August. Incidence of infection was likely much higher during the first wave than the second, but steadily increasing availability of SARS-CoV-2 testing yielded higher case counts in the second wave. An interactive web application for exploring the contact rate in Connecticut is available at https://forrestcrawford.shinyapps.io/ct_social_distancing. The Supplementary Materials describe the web application in detail.

The Supplementary Material presents a comparison of the contact rate to mobility metrics from Google [59], Apple [60], Facebook [61], Descartes Labs [14, 62], and Cuebiq [40]. Most mobility metrics provided by these companies returned to values near the February/March baseline by the beginning of July. In contrast, the contact rate shown in Figure 2 shows that close interpersonal contact stayed low and rose slowly during June–August, 2020. Mobility metrics returned more quickly to the February 2020 baseline (or higher) compared to the contact rate and do not explain the low COVID-19 incidence achieved in Connecticut during June–August, 2020.

One explanation for the discrepancy between close contact and mobility metrics is that it is possible to travel far from home, to many distinct points of interest, or to many geographic areas, without coming into close contact with others. This might be what occurred in the summer of 2020: as Connecticut began its phased reopening plan, people resumed more normal patterns of away-from-home movement – work, shopping, or recreational activities – while maintaining social distancing. For this reason, when mobility metrics are used as proxy measures of close interpersonal contact, they might overstate the risk of disease transmission.

## Prediction of COVID-19 cases in Connecticut towns

To evaluate the contact rate as a predictor of COVID-19 burden in Connecticut, we use confirmed COVID-19 case data from non-congregate settings reported to the Connecticut Department of Public Health. We excluded cases among residents of long-term care facilities, managed residential communities (e.g., assisted living facilities), or correctional institutions. We aggregated non-congregate case data by day of sample collection, by town. We obtained town-level population estimates from the American Community Survey [63, 64].

We predict transmission of SARS-CoV-2 and COVID-19 cases in Connecticut towns using a continuous-time deterministic compartmental transmission model based on the susceptible-exposed-infective-removed (SEIR) process [65]. We accommodate geographical variation in transmission within Connecticut and estimated features of COVID-19 disease progression, hospitalization, and death. The model incorporates flexible time-varying case-finding rates at the town level. We incorporate the contact rate into the time-varying transmission risk by multiplying the standardized contact rate by the product of the baseline transmission rate and the estimated number of susceptible and infectious individuals in each town. We fit the model to statewide data, and produce model projections for each of Connecticut’s 169 towns using the town population size, time-varying contact rate, estimated initial infection fraction, and time-varying case-finding rate. The model is conceptually similar to other SEIR-type COVID-19 transmission models making use of mobility data, but incorporates much geographic variation in transmission rates [26, 66–74]. The model and calibration procedure are described in detail in [65] and in the Supplementary Materials.

**Figure 3:**
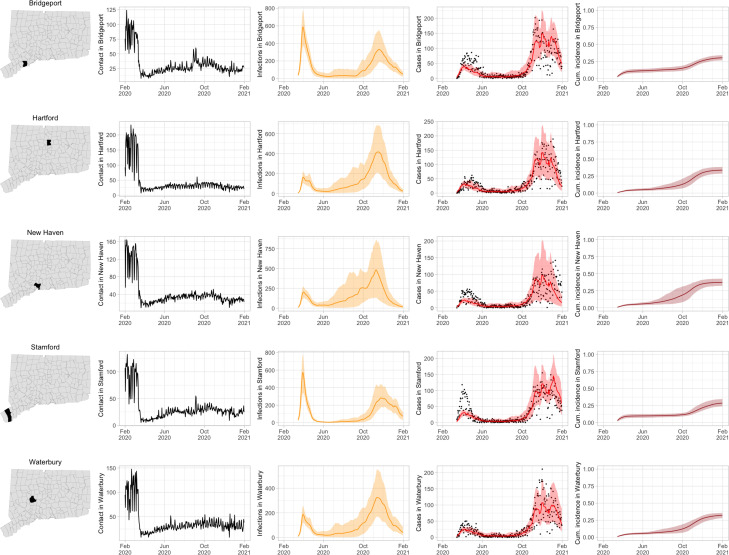
Contact rates, COVID-19 cases, and model predictions (with 95% uncertainty intervals) of infections, cases, and cumulative incidence proportion in the five largest cities by population in Connecticut: Bridgeport, New Haven, Hartford, Stamford, and Waterbury. Black dots show confirmed non-congregate COVID-19 case counts.

Figure 3 shows contact rates, estimated SARS-CoV-2 infections, observed and estimated case counts, estimated cumulative incidence, as well as 95% uncertainty intervals for model estimates, for the five largest cities by population in Connecticut: Bridgeport, Hartford, New Haven, Stamford, and Waterbury. Contact rates in these towns largely mirror rates in the state as a whole. Model estimates track the pattern of case counts through the full course of the epidemic, including the dramatic reduction in transmission during June–August. In some towns, e.g. Stamford, case counts are under-estimated in model projections during the first wave during March–April 2020. In these cases, dynamics of SARS-CoV-2 infections may differ from the dynamics of case counts because the estimated case detection rate (via viral testing) varied dramatically over time and geography.

### Role of contact in local outbreaks

**Figure 4:**
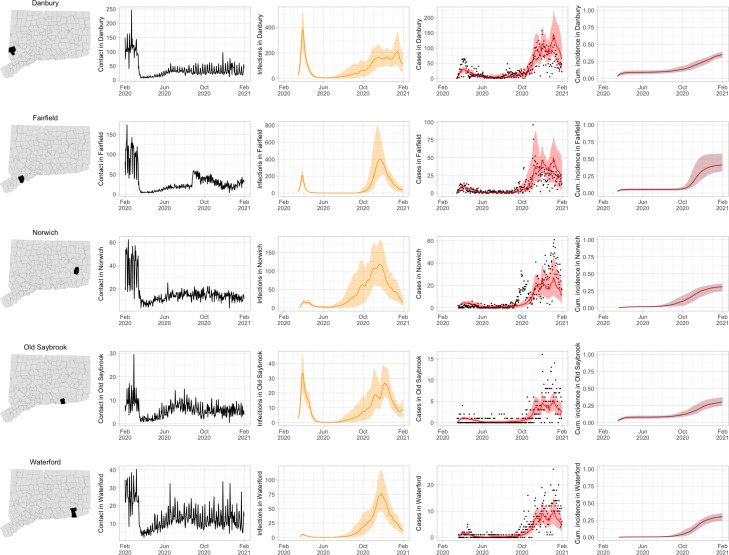
Contact rates, COVID-19 cases, and model predictions (with 95% uncertainty intervals) of infections, cases, and cumulative incidence proportion in several towns in Connecticut whose case or contact patterns differ from that of the state as a whole: Danbury, Fairfield, Norwich, Old Saybrook, and Waterford. Public health officials declared an outbreak in Danbury in mid-August 2020. Fairfield experienced outbreaks linked to two universities in September 2020. Norwich, Old Saybrook, and Waterford, in the eastern part of the state, were mostly spared during the first wave of infection, and had quickly rising case counts in fall 2020.

As COVID-19 case counts in Connecticut decreased during June–August, new and more heterogeneous patterns of transmission emerged. Figure 4 shows contact rates, confirmed non-congregate COVID-19 case counts, and 95% uncertainty intervals for cases in five Connecticut towns where incidence patterns differed from those of the larger cities shown in Figure 3.

During June–August, the only known community-wide COVID-19 outbreak in Connecticut occurred in the town of Danbury (population 84,479) [52]. During August 2–20, at least 178 new COVID-19 cases were reported, a significant increase from 40 cases reported during the prior week. Contact tracing investigations by public health officials attributed the outbreak to travel, but the contact rate was high in Danbury beginning in July and genomic analyses suggested the outbreak was closely linked to lineages already circulating in New York City and Connecticut [75, 76]. Predictions from the model including contact rates from Danbury suggest that this outbreak might have been part of a long-term increase in infections that began earlier in July and continued mostly unabated through November.

The town of Fairfield, bordering the larger city of Bridgeport, has a population of 62,105 people, and contains two universities, both of which reopened for in-person education in mid-August. The university communities experienced a surge in cases during September–October after students returned [77]. Students had access to frequent COVID-19 testing, and test coverage in this community was likely higher than in the general population, so infections among students might have been more likely to be reported to public health authorities. Contact rates in both Fairfield and the adjacent city of Bridgeport increased (Figures 3 and 4) during September shortly after students arrived on campus. The consequence of this increase in contact rate is evident in the rise in case counts for Fairfield two to three weeks later.

The eastern part of Connecticut was largely spared in the first wave of infections during March–April, but Norwich (population 39,136) and nearby towns experienced a strong surge in cases beginning in mid-September [54, 55]. Contact rose more quickly in these towns, compared to the western part of the state, following the beginning of Phase 1 in May 2020. Low testing coverage during the spring and summer of 2020, imported infections from neighboring Rhode Island, and lower compliance with social distancing measures might have played a role in outbreaks in the eastern part of the state.

Contact data do not explain all variations in confirmed non-congregate COVID-19 case counts. Though the model fits cases well overall in large cities, it can fail to capture variation in case counts in smaller cities where testing coverage is lower, or in settings where case-finding effort varied over the time. For example, high case counts corresponding to outbreak investigations involving extensive testing in Danbury during August, and Norwich during September/October, do not directly reflect changes in contact, and are not captured by the model projections.

### Contact may provide advance warning of COVID-19 cases

To assess the relationship between close interpersonal contact and COVID-19 cases without SEIR-type model assumptions about the dynamics of transmission, we fit a hierarchical Bayesian space-time statistical model to predict cases using town-level contact data. In a model that included 28 prior days of contact data, lagged contact from 3 to 7 days prior is significantly associated with current-day cases, in agreement with known features of the time to development of symptomatic disease [78–83]. An increase of 10 contacts in each of the previous 28 days within an average town gives rise to an increase in cases by a factor of 1.29 (95% credible interval [1.22, 1.37]) within that town. A model that includes contact predicts cases better than one without contact, according to goodness-of-fit criteria [84, 85]. The model structure and results are described in detail in the Supplementary Materials.

## Discussion

Public health decision-makers track the COVID-19 pandemic using metrics – syndromic surveillance data, cases, hospitalizations, deaths – that lag disease transmission by days or weeks. In this paper, we have described a method for population-level surveillance of close interpersonal contact, the primary route for person-to-person transmission of SARS-CoV-2, by using anonymized mobile device geolocation data. The contact rate can reveal high-contact conditions likely to spawn local outbreaks, or areas where residents experience high contact rates, days or weeks before the resulting cases are detected by public health authorities through testing, traditional case investigation, and contact tracing. Because mobile device geolocation data are passively collected, contact rates are invariant to allocation and availability of public health resources for case finding. For this reason, contact rates could serve as a better early-warning signal for outbreaks than cases alone, especially when test volume is low. Contact rates could also have advantages over surveillance approaches using mobility metrics because interpersonal contact within six feet is more directly related to the likelihood of disease transmission by direct contact or respiratory droplets.

Contact rates could benefit public health efforts to prevent transmission of SARS-CoV-2 in two ways. First, community engagement programs could be directed to locations where the contact rate is high to improve social distancing practices or provide additional protective measures like ensuring adequate ventilation, environmental cleaning, and mask use. Second, enhanced testing in areas with high contact rates, and residential areas of people experiencing that contact, could lead to earlier and more complete detection of cases. Earlier and more complete detection of cases enables faster and more complete isolation of cases and quarantine of contacts, which are crucial to stop transmission and stop outbreaks.

Contact rates also might be a useful addition to mathematical models of infectious disease transmission for prediction of COVID-19 infections or cases. In the early stages of the COVID-19 pandemic, researchers employed variations on the classical SEIR epidemic model [86, 87] to predict the initial wave of infections, estimate parameters like the basic reproduction number, and assess the effect of non-pharmaceutical interventions [e.g. 65, 88, 89, in Connecticut]. These models often assumed a constant population-level contact rate that is subsumed into a transmissibility parameter, or estimated contact rate from survey data collected prior to the pandemic [90, 91].

We have focused in this study on the U.S. state of Connecticut, but the usefulness of anonymized and passively collected contact data could be generalized to other settings. In the U.S., where mobile phone usage is high, states or towns can implement contact surveillance at low cost by working with private sector mobile device data providers. Like Connecticut, other states and countries experienced constrained testing availability in the early stages of the pandemic, and uneven geographic distribution of testing after test volume increased. Non-pharmaceutical interventions such as stay-at-home mandates, business and school closures, and social distancing guidelines also had uneven adoption and compliance varied across time and geography. Surveillance of contact rates could help officials better distribute testing resources and monitor intervention compliance in numerous settings. Internationally, mobile phone ownership has grown quickly but might be low in some developing countries [92], making contact surveillance less feasible in these settings.

The contact rate employed here has several advantages over existing mobility metrics and measures of mobile device density and proximity. First, the contact rate has been designed specifically to measure interpersonal contact within 6-feet relevant to COVID-19 transmission, as defined by CDC [1]. In contrast, mobility metrics primarily measure movement, which might not be a good proxy measure of close interpersonal contact. For each potential contact event between two devices, we use the reported device locations and horizontal uncertainty measurements to compute the probability that the devices were within six feet of one another. In this way, each potential contact event is weighted by the likelihood that the people carrying the devices were close enough for transmission to occur. In contrast, Unacast’s “human encounters” metric measures the frequency of two devices being within 50 meters of one another. Because the Unacast definition includes interactions that are at a distance much farther than six feet, many are unlikely to involve the potential for disease transmission. The contact rate used here incorporates close interpersonal contact occurring in every location in Connecticut, not only at pre-selected venues [e.g. 32, 93] therefore, the contact rate might be a better proxy for population-level transmission risk when there are prevalent infections.

Contact data derived from mobile device geolocation data have limitations. First, not all devices in Connecticut appear in the sample: during May 1– November 28, 2020, we observed a total of 788,842 unique device IDs, representing roughly 22% of the 3.6 million residents of Connecticut. An analysis in the Supplementary Materials shows that there is no evidence of systematic under-coverage of mobile devices as a function of town population sizes, but coverage declines slightly in towns with higher percent of residents identifying themselves as non-White, lacking a high school degree, and below the poverty level. Under-coverage among particularly vulnerable populations could result in under-counting of potential transmission events likely to affect these populations. Second, horizontal uncertainty varies by device and location, making close interpersonal contact that occurs in some areas more difficult to detect with certainty. Third, the duration of time a device was stationary is unknown because location data are reported asynchronously and at irregular intervals. Fourth, using anonymized mobile device geolocation data we do not observe individual-level demographic information, whether a potential contact occurred indoors or outdoors, nor additional individual-level infection risk factors or risk mitigation behavior like mask-wearing, hand washing, avoidance of touching surfaces or avoidance of crowded indoor spaces. However, CDC recommends the determination of close contact should be made “irrespective of whether the person with COVID-19 or the contact was wearing a mask” [94].

The contact rate might not detect all types of close interpersonal contact relevant for disease transmission and does not distinguish between physical contact and close proximity. We exclude contact occurring at primary dwell locations, so contact between pairs of people while at their shared same primary dwell locations is not represented in the contact rate. As a result our model projections may not adequately capture household transmission. Close contact that occurs while traveling, for example on a bus or train, might not be detected because devices are not stationary. Devices located on different floors of the same building might report nearby locations, even if the devices are separated by one or more floors. Location information might be reported by each device at irregular intervals, so we might not observe some kinds of fleeting contact. Contact that occurs outside of Connecticut is not recorded in our dataset. In particular, we did not observe information about contact for people who live in Connecticut and work in the New York City area.

Statewide contact rate based on mobile device geolocation data helps explain Connecticut’s success in avoiding a broad resurgence in COVID-19 cases during June–August 2020, emergence of localized outbreaks during late August–September, and a broad statewide resurgence during October–December. In addition to explaining historical patterns of transmission, incorporating contact rates into an SEIR transmission model might improve prediction of future COVID-19 cases and outbreaks at the town level, which could inform targeted allocation of public health prevention measures, such as SARS-CoV-2 testing and contact tracing with subsequent isolation or quarantine. Contact rate estimated from mobile device geolocation data can help improve population-level surveillance of close interpersonal contact, guide public health messaging campaigns to encourage social distancing, and in allocation of testing resources to detect or prevent emerging local outbreaks.

## Supplementary Material

1

## Footnotes

1 See e.g., 45 C.F.R. part 46, 21 C.F.R. part 56; 42 U.S.C. §241(d); 5 U.S.C. §552a; 44 U.S.C. §3501 et seq.
