# Supplementary material for: Impact of close interpersonal contact on COVID-19 incidence: evidence from one year of mobile device data
Source: medRxiv. 2021 Mar 12:2021.03.10.21253282. Preprint. [Version 1] doi: 10.1101/2021.03.10.21253282 (PMC7987027; doi:10.1101/2021.03.10.21253282)
Supplement: 1 [file NIHPP2021.03.10.21253282-supplement-1.pdf]

medRxiv preprint doi: https://doi.org/10.1101/2021.03.10.21253282; this version posted March 12, 2021. The copyright holder for this preprint (which was not certified by peer review) is the author/funder, who has granted medRxiv a license to display the preprint in perpetuity.
It is made available under a CC-BY 4.0 International license .

Supplement to "Impact of close interpersonal contact on COVID-19 incidence: evidence from one year of mobile device data"

Forrest W. Crawford[1,2,3,4], Sydney A. Jones[5,6], Matthew Cartter[6,7], Samantha G. Dean[1], Joshua L. Warren[1], Zehang Richard Li[7], Jacqueline Barbieri[8], Jared Campbell[8], Patrick Kenney[8], Thomas Valleau[8], Olga Morozova[9]

1. Department of Biostatistics, Yale University School of Public Health, New Haven, CT, USA.
2. Department of Statistics & Data Science, Yale University, New Haven, CT, USA.
3. Department of Ecology & Evolutionary Biology, Yale University, New Haven, CT, USA.
4. School of Management, Yale University, New Haven, CT, USA.
5. Epidemic Intelligence Service, Centers for Disease Control & Prevention, Atlanta, GA, USA.
6. Infectious Diseases Section, Connecticut Department of Public Health, New Haven, CT, USA.
7. Department of Statistics, University of California, Santa Cruz, Santa Cruz, CA, USA.
8. Whitespace Solutions, Ltd, Alexandria, VA, USA.
9. Program in Public Health and Department of Family, Population and Preventive Medicine, Stony Brook University, NY, USA.

March 10, 2021

1 Materials & Methods

1.1 Overview: computing the contact rate

Anonymized mobile device geolocation data for devices located within the state of Connecticut are received from a third-party provider, X-Mode. Mobile device users consent to sharing of their location information when they accept the terms and conditions of the applications that report location data. Mobile devices also permit users to turn off location sharing. The mobile device geolocation records contain anonymized unique device IDs that persist over time, GPS coordinates, date/time stamps, and GPS location error estimates (also called horizontal uncertainty) measured in meters. The data used in the analysis presented in this paper contain no individually identifying meta-data.

From these records we calculate the location in which each device had the most location records and designate that area as the device's primary dwell location. The primary dwell location is used to determine whether distinct devices reside at the same location. A device's primary dwell location is considered to be the best estimate of

medRxiv preprint doi: https://doi.org/10.1101/2021.03.10.21253282; this version posted March 12, 2021. The copyright holder for this preprint (which was not certified by peer review) is the author/funder, who has granted medRxiv a license to display the preprint in perpetuity.
It is made available under a CC-BY 4.0 International license .

the home location of the device user. We do not use time of day to determine primary dwell location, as this could lead to mis-characterization of primary dwell location for people who work at night instead of during the daytime, for example.

For each device, we identify clusters of records as locations where other devices were stationary and nearby. When two devices were stationary and in proximity to one another at the same time, the device locations and their corresponding GPS location errors are used to calculate a probability that the devices were in close contact, within six feet (approximately two meters). The resulting information is used to generate a contact event record including the date, time, location and horizontal accuracy of each device involved in the contact, both device IDs, and the computed probability of contact between the devices.

To avoid measuring spurious contact between people who are not actually close to one another, or contact between people who live together, we do not record contacts that occur in some places. A buffered polygon derived from roadway center lines is used to determine if a given contact event occurred on a roadway. If so, then the contact record excluded from the calculation of the contact rate within that region. This could result in missing close contact that occurs in vehicles, such as on buses, trains, or carpooling. Similarly, all contact events for devices at their estimated primary dwell location are tagged and excluded when computing contact rates.

1.2 Measuring close contact

We consider a simplified version of geospatial location information on Euclidean plane $\mathbb{R}^2$. We modify this setting below to account for the curvature of the earth. Suppose that for device location point i we observe the triple (X_i, Y_i, R_i) where (X_i, Y_i) is the reported location (in longitude and latitude) of the mobile device and R_i is the radius of horizontal uncertainty associated with the device location. We assume that the horizontal uncertainty radius R_i is the $(1-\alpha) \times 100\%$ quantile of the radial density of the device location. Specify this distribution as a symmetric bivariate Gaussian centered at the true device location (μ_x, μ_y) with covariance matrix $\sigma_i^2 I$, where I is the 2×2 identity matrix. Then (X_i, Y_i) has density

$$f(x, y|\sigma_i^2) = \frac{1}{2\pi\sigma_i^2} \exp\left[-\frac{1}{2\sigma_i^2}\left((x-\mu_x)^2 + (y-\mu_y)^2\right)\right].$$

If $R_i = r_i$ is the horizontal uncertainty associated with the $(1-\alpha) \times 100\%$ quantile radial density level set of the point i, then $r_i = \sigma_i \Phi^{-1}(1-\alpha)$, where $\Phi^{-1}(\cdot)$ is the standard normal quantile function. We can therefore

medRxiv preprint doi: https://doi.org/10.1101/2021.03.10.21253282; this version posted March 12, 2021. The copyright holder for this preprint (which was not certified by peer review) is the author/funder, who has granted medRxiv a license to display the preprint in perpetuity.
It is made available under a CC-BY 4.0 International license .

estimate the variance σ_i^2 by

$$\hat{\sigma}_i^2 = r_i^2/(\Phi^{-1}(1-\alpha))^2. \tag{1}$$

In this paper, we use $\alpha = 0.05$.

Define the Euclidean distance between the reported location of two points (X_i, Y_i, R_i) and (X_j, Y_j, R_j) as

$$D_{ij} = \sqrt{(X_i - X_j)^2 + (Y_i - Y_j)^2}$$

and fix a distance $\epsilon > 0$. In this paper, we use ϵ equal to two meters. We want to evaluate the probability that points i and j are within ϵ meters of one another. This probability can be expressed as

$$\begin{aligned} \Pr(D_{ij} \le \epsilon) &= \Pr\left(\sqrt{(X_i - X_j)^2 + (Y_i - Y_j)^2} \le \epsilon\right) \\ &= \Pr\left((X_i - X_j)^2 + (Y_i - Y_j)^2 \le \epsilon^2\right) \\ &= \Pr\left(\frac{(X_i - X_j)^2 + (Y_i - Y_j)^2}{\sigma_i^2 + \sigma_j^2} \le \frac{\epsilon^2}{\sigma_i^2 + \sigma_j^2}\right). \end{aligned} \tag{2}$$

Now under the assumption that (X_i, Y_i) and (X_j, Y_j) have independent bivariate Gaussian distribution, the variance-scaled quantity

$$\frac{(X_i - X_j)^2 + (Y_i - Y_j)^2}{\sigma_i^2 + \sigma_j^2} \tag{3}$$

follows the non-central chi-square distribution with 2 degrees of freedom and non-centrality parameter

$$\frac{(\mu_{xi} - \mu_{xj})^2 + (\mu_{yi} - \mu_{yj})^2}{\sigma_i^2 + \sigma_j^2}. \tag{4}$$

Since the true device locations and variances in (4) are not observed, we substitute the observed device locations X_i, Y_i, X_i, and Y_j, as well as the estimated variances $\hat{\sigma}_i^2$ and $\hat{\sigma}_j^2$ computed from (1). Because the variance-scaled squared distance (3) follows the non-central Chi-square distribution, the probability that the two devices are within two meters, $D_{ij} \le 2$, can be computed using standard statistical software.

In reality the Earth is not a plane and the Euclidean distance D_{ij} is shorter than the true distance between i and j on the surface of the Earth. But for distant points or those whose uncertainty radius is large, it is necessary to evaluate longer distances on the surface of the Earth. We therefore substitute the Haversine distance [1] for the Euclidean distance D_{ij} in the calculation above. The resulting Gaussian approximation is useful for small geodesic distances because we are interested in points that are less than two meters apart.

medRxiv preprint doi: https://doi.org/10.1101/2021.03.10.21253282; this version posted March 12, 2021. The copyright holder for this preprint (which was not certified by peer review) is the author/funder, who has granted medRxiv a license to display the preprint in perpetuity.
It is made available under a CC-BY 4.0 International license .

To describe computation of the contact rate, let $Z_i(t) = (X_i(t), Y_i(t), R_i(t))$ be the location and corresponding horizontal uncertainty radius for device i at time t. A *potential contact* between devices i and j at time t occurs when the locations of the two devices $Z_i(t)$ and $Z_j(t)$ are stationary and nearby. Let $D_{ij}(t)$ be the computed distance between the two points i and j. When a potential contact occurs between i and j at time t, let

$$P_{ij}(t) = \Pr(D_{ij}(t) \leq \epsilon)$$

be the probability that these devices are within ϵ meters of each other. Let A_{ad} be the set of pairs of devices for which a potential contact event occurred within area a on day d. For a potential contact between a pair $\{i, j\}$, let t_{ij} be the time of the potential contact. In area a on day d, the expected number of contacts is the sum of the probabilities of contact, across every potential contact event. We compute two contact rates for each area a and day d. First, we aggregate contact probabilities by the area in which the contact occurred. The contact rate by region of contact is

$$C_{ad}^{\text{loc}} = \sum_{\{i,j\} \in A_{ad}} P_{ij}(t_{ij}). \tag{5}$$

Next, we aggregate contacts by the region (town) of the device's primary dwell location. Let A be the set of all regions and let $h(j)$ be the primary dwell region of device j. The device home contact rate is

$$C_{ad}^{\text{home}} = \sum_{b \in A} \sum_{\{i,j\} \in A_{bd}} P_{ij}(t_{ij}) \mathbb{1}\{h(i) = a \text{ or } h(j) = a\}, \tag{6}$$

where the indicator function $\mathbb{1}\{\cdot\}$ is 1 if its argument is true, and 0 otherwise.

1.3 Community case definition

Laboratory tests for SARS-CoV-2 and cases of COVID-19 are reportable to the Connecticut Department of Public Health [2]. A confirmed case of COVID-19 is defined as detection of severe acute respiratory syndrome coronavirus 2 ribonucleic acid (SARS-CoV-2 RNA) in a clinical or autopsy specimen using a molecular amplification test [3]. In this analysis, COVID-19 cases and SARS-CoV-2 tests reported to the Connecticut Department of Public Health among persons residing in long-term care facilities, managed residential communities (e.g., assisted living facilities) or correctional institutions were excluded. Cases and tests among staff working at these locations were not excluded.

The Connecticut Department of Public Health used data from COVID-19 case report forms and laboratory test

medRxiv preprint doi: https://doi.org/10.1101/2021.03.10.21253282; this version posted March 12, 2021. The copyright holder for this preprint (which was not certified by peer review) is the author/funder, who has granted medRxiv a license to display the preprint in perpetuity.
It is made available under a CC-BY 4.0 International license .

reports to identify cases and tests among residents of congregate settings as follows. For COVID-19 cases, public health follow-up is dependent on whether the person resides in a congregate setting (facility does contact tracing) or not (local and state public health do contact tracing). Therefore, the Connecticut COVID-19 case report form includes a field for healthcare providers to indicate whether the patient resided in a congregate setting, and if so, the type of setting. Because case report forms are not received for all cases, this information might also be completed during case investigations by state or local health department staff. For this analysis, cases among congregate setting residents were excluded by using these case report form data.

To identify tests among congregate setting residents, the Connecticut DPH used a geocoding process because residence in a congregate setting is not included in the laboratory result report form, and the number of tests is too large for public health staff to review individual records. This means that the likelihood of misclassification of congregate setting residence among cases is lower than the likelihood of misclassification for tests.

The home address of each person tested is geocoded by the Connecticut Department of Public Health by using a custom composite locator that first attempts to match on the parcel, then street map data. Approximately 90% of test reports are successfully geocoded. To identify tests performed among residents of congregate settings, geocoded results are compared to a spatial layer of known long term care facilities, managed residential communities, and correctional institutions. Records geocoded to within a 150 foot buffer around these locations are categorized as tests performed among residents of congregate settings.

After the steps above were used to exclude cases and tests among congregate setting residents, the Connecticut Department of Public Health tabulated the aggregate count of cases and tests by town for each day, which were used in this analysis. No individually identifiable information was used in the analysis reported here, nor were any linkages made between mobile device data and cases and tests reported to the Connecticut Department of Public Health.

1.4 Data privacy and confidentiality

The mobile device location data contain no individually identifying meta-data. After computing the probability of contact for each potential contact event, we sum contact probabilities by day and by town using (5) and (6). No individual device geolocation data are retained or used in the analysis presented in this paper.

medRxiv preprint doi: https://doi.org/10.1101/2021.03.10.21253282; this version posted March 12, 2021. The copyright holder for this preprint (which was not certified by peer review) is the author/funder, who has granted medRxiv a license to display the preprint in perpetuity.
It is made available under a CC-BY 4.0 International license .

1.5 Transmission model for prediction of COVID-19 infections, cases, hospitalizations, and deaths

We developed a county-stratified deterministic model based on the susceptible-exposed-infective-removed (SEIR) framework to represent COVID-19 transmission and predict case counts in Connecticut using the contact rate. Here we provide a brief description of the model and calibration approach. A detailed description of the model is given in [4]. In the model, the population of each county is divided into 10 compartments. Individuals begin in the susceptible (S) compartment. Exposed individuals (E) may develop either asymptomatic (A), mild (I_M), or severe (I_S) infection. Asymptomatic and mild infections resolve without hospitalization and do not lead to death. Mild symptomatic cases self-isolate (move to R_M) shortly after development of symptoms, and transition to recovery (R) when infectiousness ceases. All severe cases require hospitalization (H) unless hospitalization capacity is exhausted, in which case they transition to $\bar{H}$ representing hospital overflow, then to recovery (R) or death (D). The model assumes a closed population and does not capture non-COVID-19 deaths. Let N_i be the population size of county i and J_i the set of counties adjacent to county i. Let $C^{(i)}$ represent hospitalization capacity in county i, which may vary over time. Transmission dynamics for county i are given by the following system of ordinary differential equations (ODE):

$$\begin{aligned}
\frac{\mathrm{d}S^{(i)}}{\mathrm{d}t} &= -\beta S^{(i)}\left[(1-k_n)\frac{I_M^{(i)}+I_S^{(i)}+k_A A^{(i)}}{N_i}+\frac{k_n}{|J_i|}\sum_{j\in J_i}\frac{I_M^{(j)}+I_S^{(j)}+k_A A^{(j)}}{N_j}\right]\\
\frac{\mathrm{d}E^{(i)}}{\mathrm{d}t} &= \beta S^{(i)}\left[(1-k_n)\frac{I_M^{(i)}+I_S^{(i)}+k_A A^{(i)}}{N_i}+\frac{k_n}{|J_i|}\sum_{j\in J_i}\frac{I_M^{(j)}+I_S^{(j)}+k_A A^{(j)}}{N_j}\right]-\delta E^{(i)}\\
\frac{\mathrm{d}A^{(i)}}{\mathrm{d}t} &= q_A\delta E^{(i)}-\alpha_A A^{(i)}\\
\frac{\mathrm{d}I_M^{(i)}}{\mathrm{d}t} &= q_{I_M}\delta E^{(i)}-\alpha_{I_M} I_M^{(i)}\\
\frac{\mathrm{d}R_M^{(i)}}{\mathrm{d}t} &= \alpha_{I_M} I_M^{(i)}-\gamma_{R_M} R_M^{(i)}\\
\frac{\mathrm{d}I_S^{(i)}}{\mathrm{d}t} &= q_{I_S}\delta E^{(i)}-\alpha_{I_S} I_S^{(i)}\\
\frac{\mathrm{d}H^{(i)}}{\mathrm{d}t} &= (1-\eta^{(i)})\alpha_{I_S} I_S^{(i)}-\gamma_H H^{(i)}\\
\frac{\mathrm{d}\bar{H}^{(i)}}{\mathrm{d}t} &= \eta^{(i)}\alpha_{I_S} I_S^{(i)}-\gamma_{\bar{H}}\bar{H}^{(i)}\\
\frac{\mathrm{d}D^{(i)}}{\mathrm{d}t} &= \gamma_H m_H H^{(i)}+\gamma_{\bar{H}} m_{\bar{H}}\bar{H}^{(i)}\\
\frac{\mathrm{d}R^{(i)}}{\mathrm{d}t} &= \alpha_A A^{(i)}+\gamma_{R_M} R_M^{(i)}+\gamma_H(1-m_H)H^{(i)}+\gamma_{\bar{H}}(1-m_{\bar{H}})\bar{H}^{(i)},
\end{aligned} \tag{7}$$

medRxiv preprint doi: https://doi.org/10.1101/2021.03.10.21253282; this version posted March 12, 2021. The copyright holder for this preprint (which was not certified by peer review) is the author/funder, who has granted medRxiv a license to display the preprint in perpetuity.
It is made available under a CC-BY 4.0 International license .

where $q_{I_M} = 1 - q_A - q_{I_S}$. The function $\eta^{(i)} = \left[1 + \exp(0.5(C^{(i)} - H^{(i)}))\right]^{-1}$ is a "soft" hospitalization capacity overflow function. The models captures infection "community" transmission in non-congregate settings, and excludes cases and deaths occurring in settings like nursing homes and prisons. We assume that recovered individuals remain immune to reinfection for the duration of the study period. The analysis was performed using the R statistical computing environment [5]. We used package deSolve to perform numerical integration of the ODE system [6].

1.5.1 Time-varying model parameters

Recognizing that many of the model parameters are unlikely to be constant over time, we represent the most critical parameters as functions of time. These parameters include: transmission parameter β, rates α_{I_M} and α_A (reciprocals of average duration of infectiousness among mildly symptomatic and asymptomatic individuals), q_{I_S} - proportion of infections, which are severe and require hospitalization, rate of hospital discharge γ_H, and hospital case fatality ratio m_H.

We use data on close contact to parameterize temporal dynamics of transmission parameter β:

$$\beta(t) = \beta_0 M_{\text{contact}}(t) \exp[B(t)],$$

where $M_{\text{contact}}(t)$ is a measure of close contact at time t relative to the pre-epidemic level, and $\exp[B(t)]$ is a function that approximates residual changes in transmission parameter β that are not explained by changes in close contact or other time-varying parameters. $B(t)$ is a smooth function obtained by applying spline smoothing on a piecewise linear function $B^*(t)$, where $B^*(t)$ is modeled with $B^*(w) = \epsilon_{[(w-t_0)/14]}$ defined on bi-weekly knots $w = \{t_0, t_0 + 14, t_0 + 28, \ldots\}$ over the observation period and linearly imputed between the knots. We model the vector of random effects $\boldsymbol{\epsilon}$ using a random walk of order one:

$$\epsilon_0 = 0, \quad \epsilon_i | \epsilon_{i-1} \sim \mathcal{N}(\epsilon_{i-1}, \sigma_\epsilon^2).$$

For the hyperparameter σ_ϵ we let

$$\frac{1}{\sigma_\epsilon^2} \sim \text{Gamma}(a_\epsilon, b_\epsilon),$$

with shape parameter $a_\epsilon = 2.5$ and rate parameter $b_\epsilon = 0.1$.

medRxiv preprint doi: https://doi.org/10.1101/2021.03.10.21253282; this version posted March 12, 2021. The copyright holder for this preprint (which was not certified by peer review) is the author/funder, who has granted medRxiv a license to display the preprint in perpetuity.
It is made available under a CC-BY 4.0 International license .

Rates of isolation or recovery α_{I_M} and α_A are assumed to depend on the testing volume. Widespread testing is assumed to identify infectious cases sooner, resulting in faster isolation and shorter duration of infectiousness. Via its relationship to age distribution of cases, probability of severe infection is assumed to change over time, likely due to higher adoption of social distancing measures by individuals with worse prognosis [7]. Time-varying rate of hospital discharge γ_H was obtained from the Connecticut Hospital Association (CHA) based on the analysis of insurance claims data. Temporal variation in the hospital case fatality ratio m_H was estimated based on data on daily hospital deaths and admissions.

Table 1 summarizes the parameters in the model and their meaning. We fix some of the model parameters whose values can be estimated based on prior research.

1.5.2 Calibration data

We calibrate the joint distribution of model parameters at the state level using the observed dynamics of confirmed COVID-19 hospitalizations census, cumulative COVID-19 hospitalizations, and cumulative number of deaths among hospitalized cases, which was obtained from the Connecticut Hospital Association [35]. In addition to close contact data, we use the following data to inform time-varying model parameters: average hospital length of stay by month (source: CHA [35]), testing volume data, and data on the age distribution of confirmed cases (source: Connecticut Department of Public Health (CT DPH) daily reports [36]). We calculated non-institutionalized county-level population and age structure in Connecticut using the 2014–2018 estimates of the American Community Survey [37]. Daily total available hospital beds (including occupied) in each county were obtained from the Connecticut Hospital Association/CHIMEData [35, 38] and used as hospitalization capacity values on a given date.

Since available hospitalizations data does not disaggregate by the patient's place of residence at the time of diagnosis or hospitalization, we estimate the time-varying distribution of hospitalizations and hospital deaths coming from congregate and non-congregate settings based on the daily distribution of COVID-19 death counts occurring in hospitals by the type of residence at the time of diagnosis (congregate vs. non-congregate) and a case fatality ratio among hospitalized residents of congregate settings. These data were obtained from Connecticut Department of Public Health.

medRxiv preprint doi: https://doi.org/10.1101/2021.03.10.21253282; this version posted March 12, 2021. The copyright holder for this preprint (which was not certified by peer review) is the author/funder, who has granted medRxiv a license to display the preprint in perpetuity.
It is made available under a CC-BY 4.0 International license .

Parameter	Meaning	Value	Source
q_A	Proportion of infections that are asymptomatic	Calibrated, prior mean = 0.4	[8–10]
$q_{I_S,0}/(1-q_A)$	Proportion of infections that are severe among symptomatic	0.065	[11–13]
β_0	Transmission parameter at baseline	Calibrated, prior mean = 1	Diffuse prior assumed
δ	1/duration of latency	1/4	Of 6 days incubation, 2 days are presymptomatic [10, 12, 14–23]
$\alpha_{A,0}$	1/duration of asymptomatic infectiousness at baseline	1/7	[16, 24–29]
k_A	Relative infectiousness of asymptomatic compared to symptomatic	0.5	[9, 15, 26, 30, 31]
$\alpha_{I_M,0}$	1/duration of symptomatic infectiousness at baseline	1/4	[11, 12, 14]; of 4 days, 2 days are presymptomatic [15–17]
γ_{R_M}	1/duration of isolation among mild symptomatic	1/7	[24, 28, 29, 32]
α_{I_S}	1/time between infectiousness onset and hospitalization	Calibrated, prior mean = 1/9	[15–17, 33, 34]
γ_H	1/length of hospital stay	Time-varying	CHA data on average hospital length of stay by month [35]
$m_{H,0}$	Hospital case fatality ratio at baseline	Calibrated, prior mean = 0.13	Estimated from CHA COVID-19 hospitalization data [35]
k_n	Proportion of contact between adjacent counties	0.015	Assumed
E_0	Number of exposed individuals at the epidemic onset	Calibrated, prior mean = 150	Assumed
L_H	Hospitalization reporting lag	Calibrated, uniform prior on integers between 5 - 14 days	Assumed
L_D	Death reporting lag	Calibrated, uniform prior on integers between 5 - 14 days	Assumed

Table 1: Transmission model parameters. Fixed parameters are drawn from the cited sources. Calibrated parameters are estimated using the Bayesian procedure described below. Durations are measured in days.

medRxiv preprint doi: https://doi.org/10.1101/2021.03.10.21253282; this version posted March 12, 2021. The copyright holder for this preprint (which was not certified by peer review) is the author/funder, who has granted medRxiv a license to display the preprint in perpetuity.
It is made available under a CC-BY 4.0 International license .

1.5.3 Model calibration and Bayesian posterior inference

We calibrate a joint posterior distribution of model parameters $\boldsymbol{\theta} = (\beta_0, q_A, \alpha_{I_S}, m_{H,0}, E_0, L_H, L_D, \boldsymbol{\epsilon})$ and hyperparameters $\boldsymbol{\sigma} = (\sigma_h, \sigma_u, \sigma_d, \sigma_\epsilon)$ to the observed data using a Bayesian approach with a Gaussian likelihood. We also accommodate reporting lags in observed hospitalizations (L_H) and hospital deaths (L_D). The distributions of hospitalizations census, cumulative hospitalizations, and hospital deaths are given by:

$$h(t) \sim \mathcal{N}\left(H(t, L_H, \boldsymbol{\theta}), \sigma_h^2\right), \tag{8}$$

$$u(t) \sim \mathcal{N}\left(U(t, L_H, \boldsymbol{\theta}), \sigma_u^2\right), \tag{9}$$

$$d(t) \sim \mathcal{N}\left(D(t, L_D, \boldsymbol{\theta}), \sigma_d^2\right), \tag{10}$$

where $H(t, L_H, \boldsymbol{\theta})$, $U(t, L_H, \boldsymbol{\theta})$, and $D(t, L_D, \boldsymbol{\theta})$ are model-projected hospitalizations census (lagged by L_H), cumulative hospitalizations (lagged by L_H), and cumulative hospital deaths (lagged by L_D) at time t under parameter values $\boldsymbol{\theta}$. Reporting lags L_H and L_D are correlated with other model parameters, including latency period, time between infection and hospitalization, time between infection and death, and length of hospital stay, therefore L_H and L_D should not be strictly interpreted as reporting lags. We assume the date of epidemic onset to be February 16, 2020 - 21 days before the first case was officially confirmed in Connecticut on March 8th, 2020, and initialize the model with E_0 exposed individuals at the time of epidemic onset and set the initial size of all downstream compartments to be zero. We assume a prior distribution on E_0 and calibrate it along with other model parameters. The county-level distribution of E_0 is fixed and was estimated based on the county population size and dates of first registered case and death in each county. We put the same independent Inv-Gamma(a, b) prior on the three hyperparameters σ_h^2, σ_u^2, and σ_d^2 with $a = 50$ and $b = 5 \times 10^7$.

We construct the joint posterior distribution over unknown parameters $(\boldsymbol{\theta}, \boldsymbol{\sigma})$ as

$$\begin{aligned} p(\boldsymbol{\theta}, \boldsymbol{\sigma} | h(t), u(t), d(t)) \propto p(\boldsymbol{\theta}) p(\boldsymbol{\sigma}) & \prod_{t \in t_H} \left[p\big(h(t) | H(t, L_H, \boldsymbol{\theta}), \sigma_h\big)\right]^{w_h z(t)} \\ & \prod_{t \in t_U} \left[p\big(u(t) | U(t, L_H, \boldsymbol{\theta}), \sigma_u\big)\right]^{w_u z(t)} \\ & \prod_{t \in t_D} \left[p\big(d(t) | D(t, L_D, \boldsymbol{\theta}), \sigma_d\big)\right]^{w_d z(t)}. \end{aligned} \tag{11}$$

Each likelihood term is weighted by $z(t)$ (observation weight at time t) times the weight assigned to a respective time series. We set $w_h = 0.89$, $w_u = 0.01$, and $w_d = 0.1$. We place a large weight on the hospitalizations

medRxiv preprint doi: https://doi.org/10.1101/2021.03.10.21253282; this version posted March 12, 2021. The copyright holder for this preprint (which was not certified by peer review) is the author/funder, who has granted medRxiv a license to display the preprint in perpetuity.
It is made available under a CC-BY 4.0 International license .

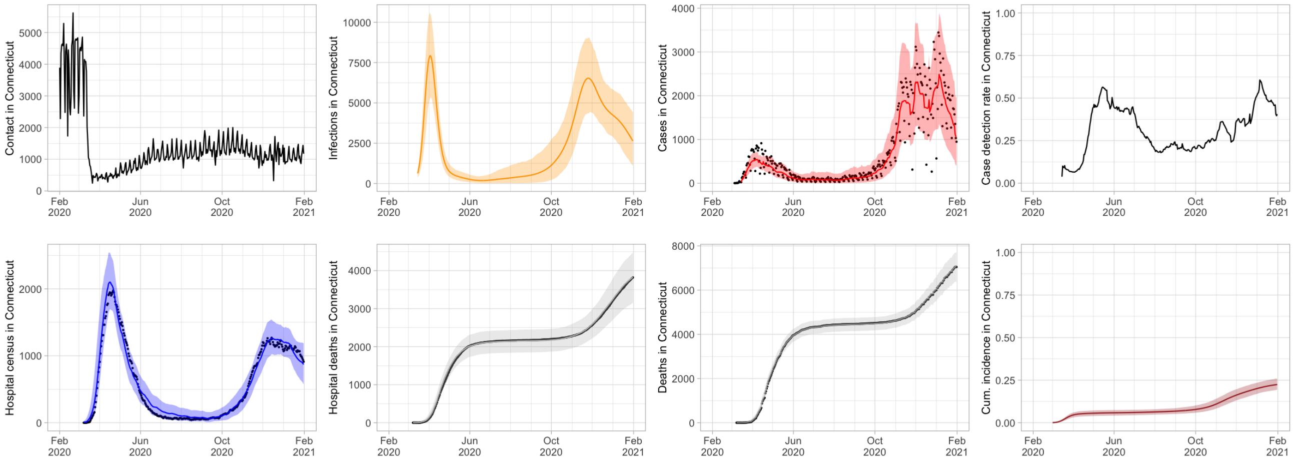

Figure 1: Connecticut statewide data and calibrated model projections. Top row from left to right: statewide contact, projected daily infections, projected (red line) and observed (black dots) cases, estimated statewide case detection rate. Bottom row from left to right: projected and observed hospital census, projected and observed hospital deaths, projected and observed total deaths, estimated cumulative incidence. Model projections are shown as lines with 95% posterior intervals. Observed data is shown as black dots.

census, since this time series is most sensitive to changes in epidemic dynamics, and a small weight on cumulative hospitalizations, since it measures a feature that is related to hospitalizations census. The range of observation times differ for different time series. For hospitalizations census and deaths, observation times start with the first non-zero observation. For cumulative hospitalizations, observation times start on May 29, 2020 when this indicator started being reported routinely. The last observation in all three time series is from February 15, 2021.

Sampling from the joint posterior distribution of (θ, σ) given in (11) is performed using Markov Chain Monte Carlo (MCMC). We employ a hybrid algorithm that combines elliptical slice sampling (ESS) [39], Gibbs sampling, and Metropolis-Hastings sampling with random walk proposals. Details of the algorithm implementation are provided in [4]. Bottom row of Figure 1 shows statewide model fit to hospital census, hospital deaths, and total deaths.

1.5.4 Model projections for case counts at the town level

To produce town-level projections, we use parameters estimated from the state-level joint posterior distribution and town-level inputs. Town-level data consist of the normalized close contact function, town population size, and town-specific initial model compartment conditions. Normalized close contact at the town level is obtained by adding an intercept to the town level contact that equals the median difference between the state and the

medRxiv preprint doi: https://doi.org/10.1101/2021.03.10.21253282; this version posted March 12, 2021. The copyright holder for this preprint (which was not certified by peer review) is the author/funder, who has granted medRxiv a license to display the preprint in perpetuity.
It is made available under a CC-BY 4.0 International license .

town-level contact for all dates t after April 7, 2020. This step enables the application of a random effects vector ϵ estimated at the state-level to the town-level contact data. The normalization approach preserves town-level contact dynamics and contact levels in each town early in the epidemic and immediately after the implementation of social distancing measures. Town-specific initial conditions were computed from the state-level posterior distribution of E_0 based on the town population size and its proximity to New York City.

We simulate epidemic dynamics for each of the 169 Connecticut towns using the model given in (7), modified to include only data from a single town. Using the model-projected dynamics of lagged daily infections within the town, we estimate the time-varying COVID-19 case-finding rate for each town using a log-linear regression model:

$$\mathbb{E}[\log(\text{Cases}_{it})] = \alpha_0 + \alpha_i + \gamma_t + \beta \log(\text{Infections}_{i(t-l)}),$$

where Cases_{it} is the 7-day moving average of reported COVID-19 cases in town i at time t, α_i is a town effect, γ_t is a time effect measured daily and $\text{Infections}_{i(t-l)}$ is the model-projected number of daily infections in town i at time $t-l$, and we set $l = 14$ days. As a final step to produce model-based estimates of daily cases in each of 169 Connecticut towns, we truncate regression-estimated town-level case-finding rate at 1 and apply this estimated rate to model-projected daily infections. The top row of Figure 1 shows statewide contact measure, projected infections, estimated and observed non-congregate COVID-19 case counts, and estimated case-detection rate. Estimates of statewide case counts is obtained by summing up estimated cases across 169 Connecticut towns.

1.6 Space-time regression model

To assess the relationship between close interpersonal contact and COVID-19 cases without assumptions about the dynamics of transmission, we develop a statistical model for describing the associations between town-level contact in previous days and current day COVID-19 cases using a distributed lag negative binomial regression framework. We emphasize that this “phenomenological” regression model does not use any SEIR-type assumptions about the relationship of contact, infections, and cases. Our purpose is to validate the usefulness of contact data in prediction of future case counts throughout the state.

In the model, each town has a unique set of parameters that describe the lagged associations with past contact, as we hypothesize that there may be spatial differences in these associations due to town-level features (e.g., population density). Spatial correlation between these parameters is also modeled since towns close to each

medRxiv preprint doi: https://doi.org/10.1101/2021.03.10.21253282; this version posted March 12, 2021. The copyright holder for this preprint (which was not certified by peer review) is the author/funder, who has granted medRxiv a license to display the preprint in perpetuity.
It is made available under a CC-BY 4.0 International license .

other may exhibit similar spatial patterns. Specifically, the model is given as

$$Y_{it}|r, p_{it} \overset{\text{ind}}{\sim} \text{Negative Binomial}\left(r, p_{it}\right), \ i = 1, \ldots, n, \ t = 1, \ldots, m,$$
$$\text{logit}\left(p_{it}\right) = \mathbf{x}_{it}^{\mathsf{T}}\boldsymbol{\beta} + \sum_{j=1}^{d} \text{z}_{i,t-j}\theta_{ij} + \eta_i + \lambda_t \tag{12}$$

where Y_{it} is the number of COVID-19 cases recorded in town i on day t, n is the total number of towns in the analysis ($n = 169$), and m is the total number of included days of data ($m = 351$). In our application of the model, the first day of analysis ($t = 1$) occurs on February 29th, 2020 and the final day ($t = 351$) occurs on February 13th, 2021.

In (12), we use the logit link function to connect the underlying probability that controls the expected number of daily cases in a location to the covariates of interest, where $\mathbf{x}_{it}$ is a vector of town-specific non-time varying covariates (i.e., median household income, total population, percent of the population living below the poverty level, percent of the population that is non-Hispanic White, percent of the population with some college education, and percent of the population that is $\geq$ 65 years) along with a day-of-the-week indicator to account for systematic case reporting patterns across time; $\boldsymbol{\beta}$ is the corresponding vector of regression parameters. Contact in town i on day t is denoted as z_{it}, and in our application of the model we include contact up to $d = 28$ days (i.e., 4 weeks) in the past. Town-specific (η_i) and time-specific (λ_t) random effects are included to account for separable spatial and temporal correlation, respectively, in the case data.

The θ_{ij} parameters are town- and lag-specific, and describe the association between contact j days earlier in the town and current day cases. The collection of all lag parameters from town i, $\boldsymbol{\theta}_i = \left(\theta_{i1}, \ldots, \theta_{i28}\right)^{\mathsf{T}}$, are decomposed into "global" and "local" effects to account for similarities in associations shared across all towns and town-specific deviations from that trend such that

$$\boldsymbol{\theta}_i = \boldsymbol{\theta} + \widetilde{\boldsymbol{\theta}}_i, \ i = 1, \ldots, n$$

where $\boldsymbol{\theta} = \left(\theta_1, \ldots, \theta_{28}\right)^{\mathsf{T}}$ represents the vector of parameters shared across all towns and $\widetilde{\boldsymbol{\theta}}_i = \left(\widetilde{\theta}_{i1}, \ldots, \widetilde{\theta}_{i28}\right)^{\mathsf{T}}$ are town-specific deviations from the global pattern. We anticipate that the global distributed lag parameters closer together in lag time will have similar behavior and model this directly by specifying

$$\boldsymbol{\theta}|\tau_{\theta}^2, \phi \sim \text{MVN}\left(\mathbf{0}_{28}, \ \tau_{\theta}^2\Sigma\left(\phi\right)\right)$$

medRxiv preprint doi: https://doi.org/10.1101/2021.03.10.21253282; this version posted March 12, 2021. The copyright holder for this preprint (which was not certified by peer review) is the author/funder, who has granted medRxiv a license to display the preprint in perpetuity.
It is made available under a CC-BY 4.0 International license .

where MVN represents the multivariate normal distribution, $\mathbf{0}_{28}$ is a vector of 28 zeros, and the correlation between the global lag parameters is defined by

$$\text{Corr}\left(\theta_j, \theta_{j'}\right) = \Sigma\left(\phi\right)_{jj'} = \exp\left\{-\phi|j - j'|\right\}.$$

The variance and smoothness of the process are described by τ_θ^2 and ϕ, respectively, where smaller values of ϕ suggest higher correlation between parameters across the lags.

Spatial and cross-correlations between the town-specific lag parameters ($\widetilde{\boldsymbol{\theta}}_i$) and town-specific intercepts (η_i) are modeled using a multivariate conditional autoregressive model [40] given as

$$\boldsymbol{\psi}_i = \left(\eta_i, \widetilde{\boldsymbol{\theta}}_i^{\text{T}}\right)^{\text{T}} | \boldsymbol{\psi}_{-i}, \rho, \Omega \overset{\text{ind}}{\sim} \text{MVN}\left(\frac{\rho \sum_{j=1}^n w_{ij} \boldsymbol{\psi}_j}{\rho \sum_{j=1}^n w_{ij} + 1 - \rho}, \frac{\Omega}{\rho \sum_{j=1}^n w_{ij} + 1 - \rho}\right), \ i = 1, \dots, n$$

where $\boldsymbol{\psi}_{-i}$ is the entire set of $\boldsymbol{\psi}_j$ vectors with $\boldsymbol{\psi}_i$ removed and w_{ij} is an indicator describing whether towns i and j share a common border or not. As is standard practice, towns are not considered to be neighbors of themselves, resulting in $w_{ii} = 0$ for all i. This model specifies that *a priori*, the set of parameters from a specific town have a multivariate normal distribution with a mean vector equal to a weighted mean of its neighbors vectors. The amount of spatial similarity between towns is controlled by $\rho \in (0, 1)$ where a value of ρ close to zero indicates near independence and close to one results in strong spatial smoothing. The cross-correlation between parameters within a given town is described by the unstructured covariance matrix Ω.

Finally, λ_t represents a flexible time trend that is common to all towns in the analysis and is defined using an autoregressive model such that

$$\lambda_t | \lambda_{t-1}, \alpha, \tau_\lambda^2 \overset{\text{ind}}{\sim} \text{N}\left(\alpha\lambda_{t-1}, \tau_\lambda^2\right), \ t = 1, \dots, m$$

where $\lambda_0 \equiv 0$ and $\alpha \in (0, 1)$ controls the level of similarity across time. This effect serves to account for temporal correlation between the case counts and describe global patterns of risk that vary across time.

1.6.1 Prior distributions

We complete the model by specifying prior distributions for all introduced parameters. When possible, we opt for weakly informative priors so that the data are the main drivers of the inference. The priors are given

medRxiv preprint doi: https://doi.org/10.1101/2021.03.10.21253282; this version posted March 12, 2021. The copyright holder for this preprint (which was not certified by peer review) is the author/funder, who has granted medRxiv a license to display the preprint in perpetuity.
It is made available under a CC-BY 4.0 International license .

as $r \sim$ Discrete Uniform $[1, 20]$, $\beta_j \overset{\text{iid}}{\sim}$ N $(0, 100^2)$ for all j, $\tau_\lambda^2, \tau_\theta^2 \overset{\text{iid}}{\sim}$ Inverse Gamma $(0.01, 0.01)$, $\alpha, \rho \overset{\text{iid}}{\sim}$ Uniform $(0.00, 1.00)$, $\phi \overset{\text{iid}}{\sim}$ Gamma $(1.00, 1.00)$, and $\Omega^{-1} \sim$ Wishart $(30, I_{29})$ where I_{29} is the 29 by 29 identity matrix.

1.6.2 Competing methods

To determine the importance of previous days contact on explaining trends in current day COVID-19 case counts, we also test two competing modeling options. First, we consider the “Global Contact” model, where (12) is modified such that $\boldsymbol{\theta}_i$ is replaced with $\boldsymbol{\theta}$ ($\boldsymbol{\theta}$ previously described). As a result, in Global Contact there are no town-specific lag parameters, only a single set that is shared across all towns. We expect this model to perform poorly compared to the full spatio-temporal model in (12) if there is spatial variability between these associations. Next, we consider the “No Contact” model, which modifies (12) by removing all of the contact information entirely. This model is likely to struggle to explain variability in the case data if prior days contact are important predictors. We note that both of these competing models technically represent subsets of the full spatio-temporal model in (12). For example, when $\widetilde{\boldsymbol{\theta}}_i \equiv \mathbf{0}$, the full model becomes Global Contact and when $\boldsymbol{\theta}_i \equiv \mathbf{0}$ it becomes No Contact. Due to this flexibility, we anticipate that the full model will perform well regardless of the spatio-temporal trends in the data.

We fit each method to the data and compare the findings using multiple methods. First, we use Watanabe–Akaike information criteria (WAIC) [41] to compare the explanatory ability of each method. WAIC is a Bayesian model selection tool that identifies the model, among a small subset of competitors, that has the best balance of fit to the data and complexity, with smaller WAIC values being preferred. Next, we consider a posterior predictive model comparison tool focused on the predictive ability of each method [42]. Specifically, we calculate the posterior mean of the deviance of the negative binomial regression model evaluated at the observed data and data generated from the posterior predictive distribution (from the same design points as the observed data). Smaller values of this metric suggest improved predictive performance of the corresponding method.

1.6.3 Model fitting

We fit all models using Markov chain Monte Carlo sampling techniques, including a Pólya-Gamma latent variable approach for obtaining semi-conjugacy for many of the parameters within the negative binomial regression

medRxiv preprint doi: https://doi.org/10.1101/2021.03.10.21253282; this version posted March 12, 2021. The copyright holder for this preprint (which was not certified by peer review) is the author/funder, who has granted medRxiv a license to display the preprint in perpetuity.
It is made available under a CC-BY 4.0 International license .

model [43]. From each model, we collect 100,000 samples from the joint posterior distribution after removing the first 10,000 iterations prior to convergence of the model. We further thin the samples by a factor of 10 to reduce posterior autocorrelation, resulting in 10,000 samples with which to make posterior inference. Convergence of the models was assessed by visually inspecting individual parameter trace plots and calculating Geweke's diagnostic [44] for all relevant parameters. Neither tool suggested any obvious signs of non-convergence.

2 Supplementary Text

2.1 Mobile device coverage and representativeness in Connecticut

We investigated the coverage of mobile devices in the sample by town population, percent who do not identify as "only white", percent without a high school degree, and percent below the poverty level as defined by the federal government's official poverty threshold, which accounts for household size, number of children, and age of householder. Demographic data were obtained from the American Community Survey [37, 45]. Figure 2 shows that device coverage tracks population size throughout the state, but coverage decreases slightly with the percent non-white, the percent that lack a high school degree, and percent below the poverty level. The town of Union is the smallest town in Connecticut, with a total population of 873 people. We do not know whether lower coverage is due to a lower percentage of town residents owning a mobile device, or because of systematic under-coverage of devices from these towns in our sample.

We received mobile device geolocation data in two overlapping batches, using different methods of anonymizing device IDs. In the first batch, covering February 1 – May 31, 2020, we observed a total of 573,452 unique device IDs in Connecticut. The average number of unique device IDs per day was 141,617. Each week an average of 80.5% of the device IDs carried over to the next week. In the second batch, covering May 1, 2020 to January 31, 2021, we observed a total of 788,842 unique device IDs in Connecticut. The average number of unique device IDs per day was 114,946. Each week an average of 77.6% of the device IDs carried over to the next week. Connecticut has a population of 3.565 million people [37, 45]. Contacts occurring within a given town are not captured if we cannot calculate a primary device dwell location for both devices involved in the contact. Individual device IDs might drop out of the sample for several reasons:

- the device leaves the state of Connecticut,

medRxiv preprint doi: https://doi.org/10.1101/2021.03.10.21253282; this version posted March 12, 2021. The copyright holder for this preprint (which was not certified by peer review) is the author/funder, who has granted medRxiv a license to display the preprint in perpetuity.
It is made available under a CC-BY 4.0 International license .

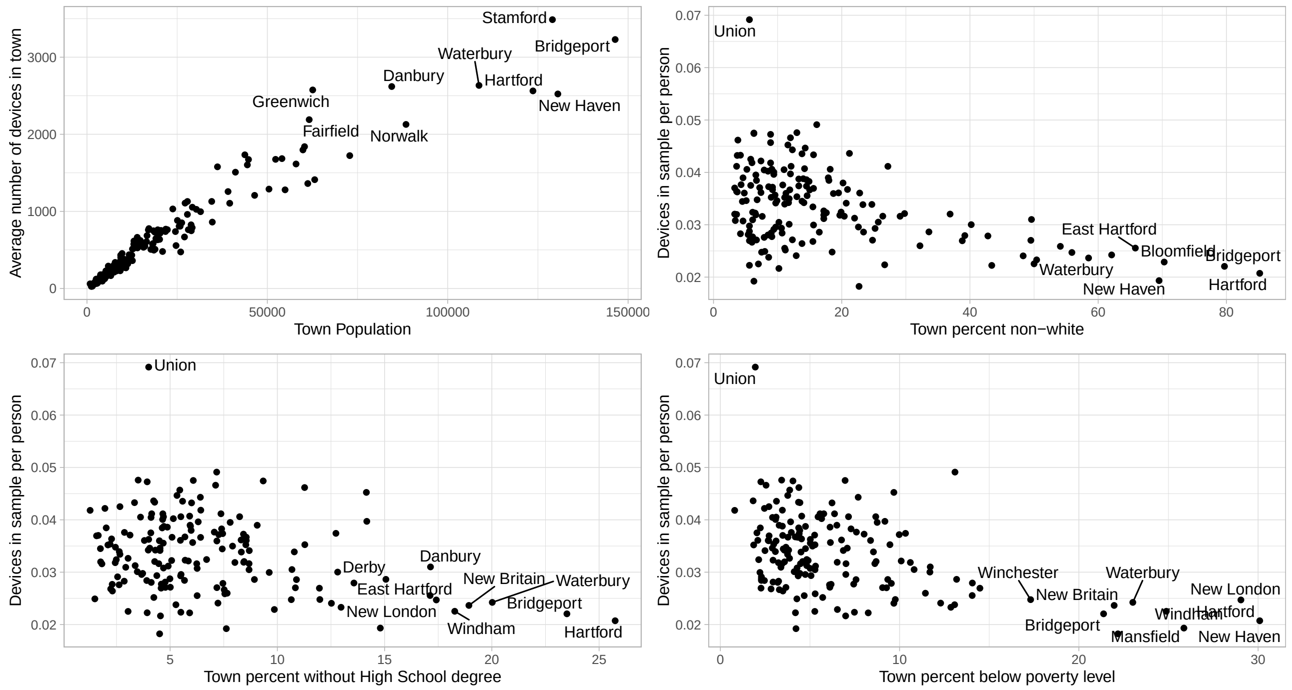

Figure 2: Number of mobile devices in the sample by Connecticut town. Top left: average number of devices in each town by total town population; top right: devices in the sample per person by town percent not identifying as "only white"; bottom left: devices in the sample per person by percent without a high school degree; and bottom right: devices in the sample per person by percent below the poverty level.

- the user uninstalls the applications reporting data to X-Mode,
- the user stops using an application that reports location data only while in use,
- the user rotates their device ID, or
- the user gets a new device with a new advertising ID.

Figure 3 shows the number of unique devices per day in the Connecticut sample.

2.2 Interactive web application

We created an interactive web application to allow users to explore contact patterns in Connecticut over time, available at `https://forrestcrawford.shinyapps.io/ct_social_distancing/`. Figure 4 shows a screen shot of the interactive application. The application uses the Shiny framework [46] for the R computing environment [5], Leaflet for mapping [47], and tigris for shapefiles [48]. The map explorer is based in part on Edward Parker's COVID-19 tracker [49] and code [50].

medRxiv preprint doi: https://doi.org/10.1101/2021.03.10.21253282; this version posted March 12, 2021. The copyright holder for this preprint (which was not certified by peer review) is the author/funder, who has granted medRxiv a license to display the preprint in perpetuity.
It is made available under a CC-BY 4.0 International license .

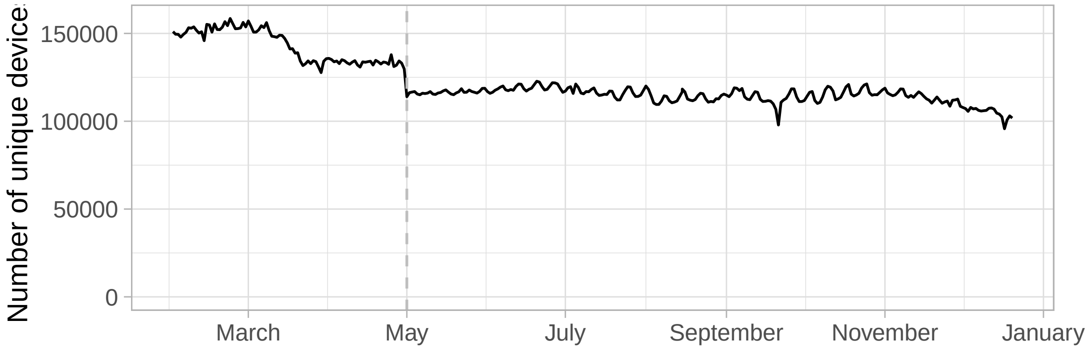

Figure 3: Number of unique mobile device IDs per day in the Connecticut sample. The vertical dashed line on May 1, 2020 corresponds to a change in the unique device ID coding from the data supplier.

The application shows contact by location of contact (5) and by device primary dwell town 6 for each day, at the town and census block group levels. Users can view the contact maps over time from February 1, 2020 to the present, as well as time trends of contact at the state and local levels. The application shows the top contact towns and census block groups throughout Connecticut, as well as points of interest – businesses, schools, hospitals – that help identify block groups.

2.3 Comparison of contact rate to mobility metrics

In order to compare the contact rate described in this paper to other mobility metrics, we acquired Connecticut mobility data from Google [51], Apple [52], Facebook [53], Descartes Labs [54, 55], and Cuebiq [56]. Google and Apple provide public data access, while Facebook and Cuebiq provide data through their respective Data For Good programs. We normalized all metrics to a day-of-week baseline using data from January or February depending on availability, and plot their percent change from baseline from February 2020 through January 2021.

Apple state-level data measures Apple Maps routing requests, categorized as transit, walking, or driving. Map routing requests are a proxy for mobility but might not represent actual trips. Movements for which Apple Maps directions are not needed, such as everyday trips for work, school, or shopping, might not be represented in routing request metrics. Figure 5 shows mobility metrics published by Apple using the day-of-week median during February 2 – February 29, 2020 as a baseline. While transit use remained below baseline during March 2020 through January 2021, driving and walking returned to baseline in June 2020. Driving and walking remained above baseline until November 2020, at which point they returned to near baseline through January 2021.

medRxiv preprint doi: https://doi.org/10.1101/2021.03.10.21253282; this version posted March 12, 2021. The copyright holder for this preprint (which was not certified by peer review) is the author/funder, who has granted medRxiv a license to display the preprint in perpetuity.
It is made available under a CC-BY 4.0 International license .

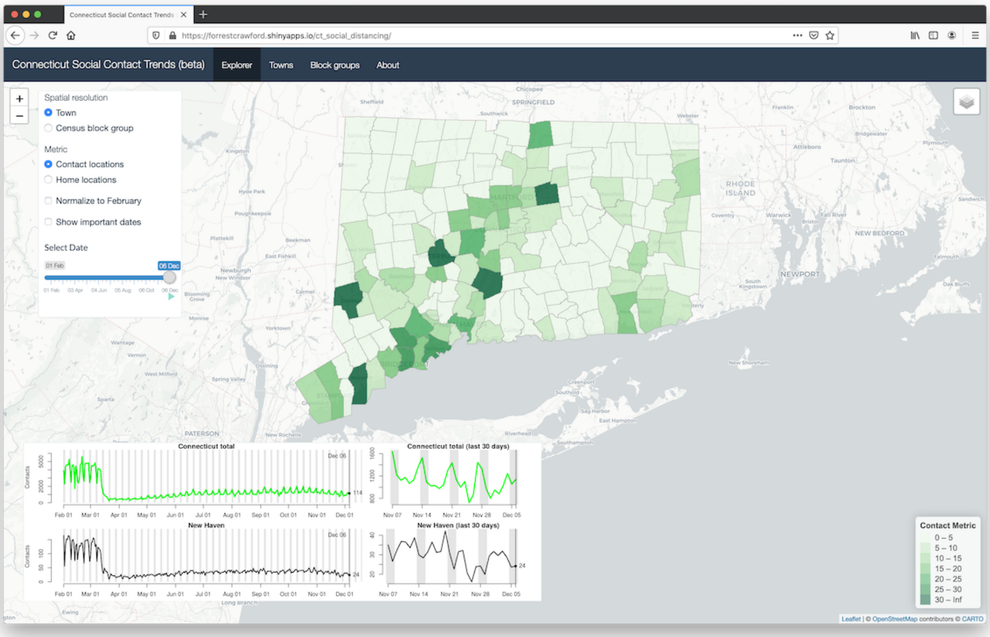

Figure 4: Interactive web application showing contact in Connecticut towns on December 6, 2020. The application can display the locations where contact is occurring or contact by the town of device primary dwell town.

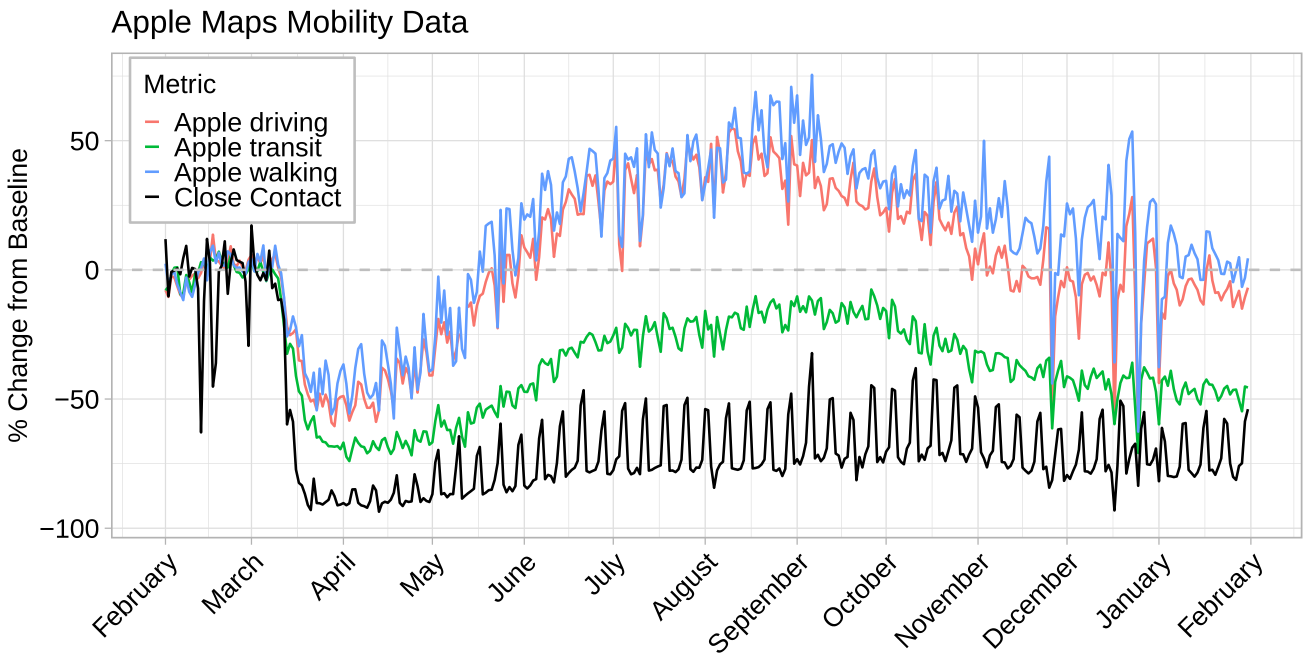

Figure 5: Comparison of Apple Maps mobility metrics to the contactr rate described in this paper during February 1 – January 31, 2021.

medRxiv preprint doi: https://doi.org/10.1101/2021.03.10.21253282; this version posted March 12, 2021. The copyright holder for this preprint (which was not certified by peer review) is the author/funder, who has granted medRxiv a license to display the preprint in perpetuity.
It is made available under a CC-BY 4.0 International license .

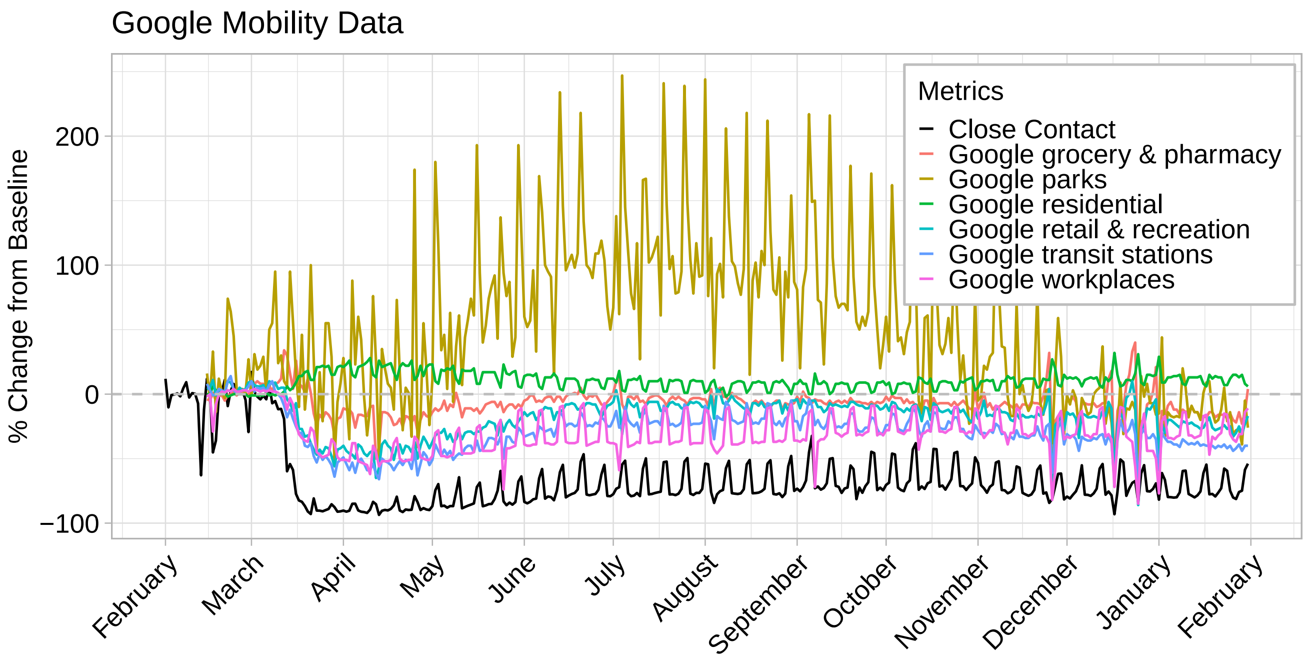

Figure 6: Comparison of Google mobility metrics to the contact rate described in this paper during February 1 – January 31, 2021.

Google state-level mobility data measured visits to areas of interest, categorized as grocery and pharmacy, parks, residential, retail and recreation, transit stations, and workplaces. More detailed information about the definitions of these areas of interest, and the completeness of these categories, is not available. Figure 6 shows mobility metrics published by Google using the day-of-week median from January 3, 2020 to February 6, 2020 as the baseline. All categories other than transit stations and workplaces returned to near baseline levels by summer 2020, and all categories other than residential remained near or below baseline throughout winter 2020.

Facebook county-level mobility data measured the number of 600m by 600m geographic units visited by a device in a day. This metric summarizes how mobile people from different counties are, but might not represent the distance of travel, time away from home, or potential close contacts with others. Figure 7 shows mobility metrics published by Facebook with day-of-week mean during February 2 – February 29, 2020 (excluding February 17) as the baseline. Facebook mobility levels returned to near baseline in all Connecticut counties by July 2020, with little difference between counties. From fall 2020 through January 2021, Facebook mobility levels for all counties decreased to slightly below baseline levels.

Cuebiq county-level mobility data measures a 7 day rolling average of the median distance traveled in a day,

medRxiv preprint doi: https://doi.org/10.1101/2021.03.10.21253282; this version posted March 12, 2021. The copyright holder for this preprint (which was not certified by peer review) is the author/funder, who has granted medRxiv a license to display the preprint in perpetuity.
It is made available under a CC-BY 4.0 International license .

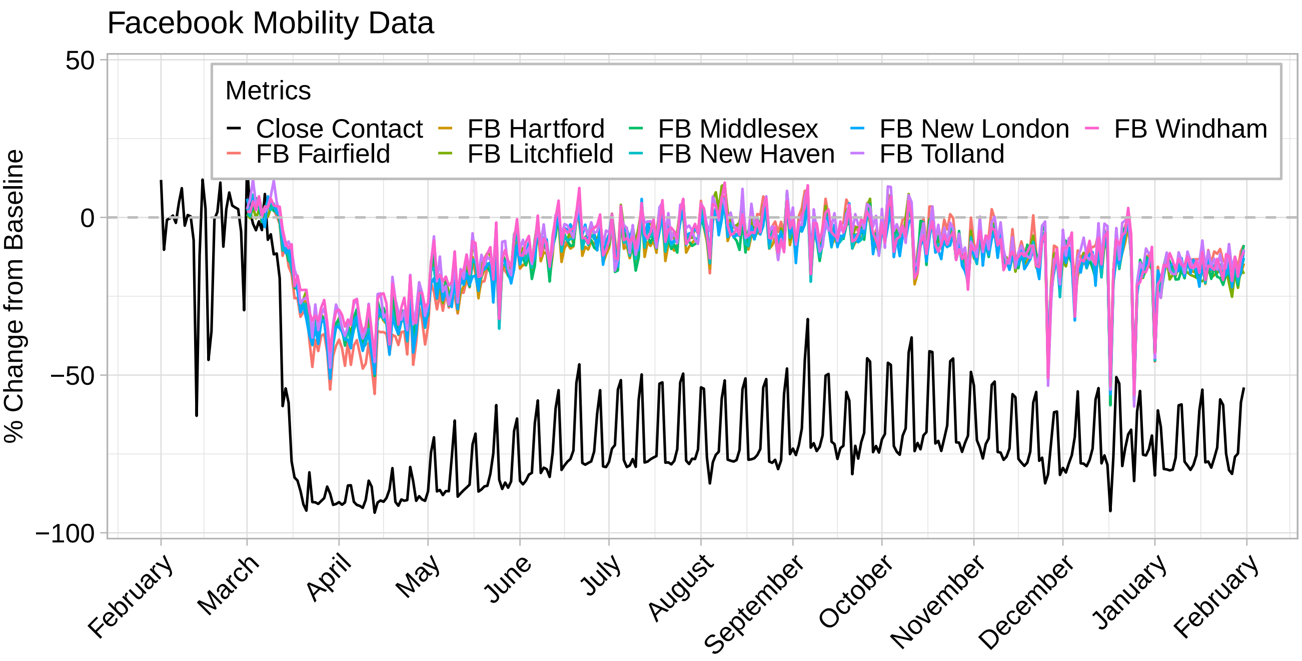

Figure 7: Comparison of Facebook (FB) mobility metrics to the contact rate described in this paper during February 1 – January 31, 2021.

and was available through November 1, 2020 [1]. Figure 8 shows mobility data provided by Cuebiq with day-of-week median during February 2 – February 29, 2020 as the baseline. By July 2020, Cuebiq mobility levels returned to near baseline. Figure 9 shows Cuebiq's metric for "contact", when two or more devices are within 50 feet of each other within five minutes. Information about whether this metric takes spatial error (horizontal uncertainty) into account is not available. In July 2020, Cuebiq contact levels remained further below baseline than the Cuebiq mobility metric. The Cuebiq 50-foot contact metric was closer to baseline than our calculated contact rate during summer and fall 2020.

Finally, Descartes Labs state-level mobility data represents maximum distance devices have moved from the first reported location in a given day. Figure 10 shows the mobility metric provided by Descartes Labs with day-of-week median during February 17 – March 7, 2020 as the baseline. It was exceptional amongst the data sources in that mobility remained notably below baseline during March 2020 – January 2021. However, the percent decline in close contact was consistently larger than the observed percent decline in the Descartes Labs mobility metric.

Every mobility metric we studied returns to baseline faster than the contact rate, though Descartes Labs' mo-

[1] Aggregated mobility data were provided by Cuebiq, a location intelligence and measurement platform. Through its Data for Good program, Cuebiq provides access to aggregated mobility data for academic research and humanitarian initiatives. This first-party data is collected from anonymized users who have opted-in to provide access to their location data anonymously, through a GDPR-compliant framework. It is then aggregated to the census-block group level to provide insights on changes in human mobility over time.

medRxiv preprint doi: https://doi.org/10.1101/2021.03.10.21253282; this version posted March 12, 2021. The copyright holder for this preprint (which was not certified by peer review) is the author/funder, who has granted medRxiv a license to display the preprint in perpetuity.
It is made available under a CC-BY 4.0 International license .

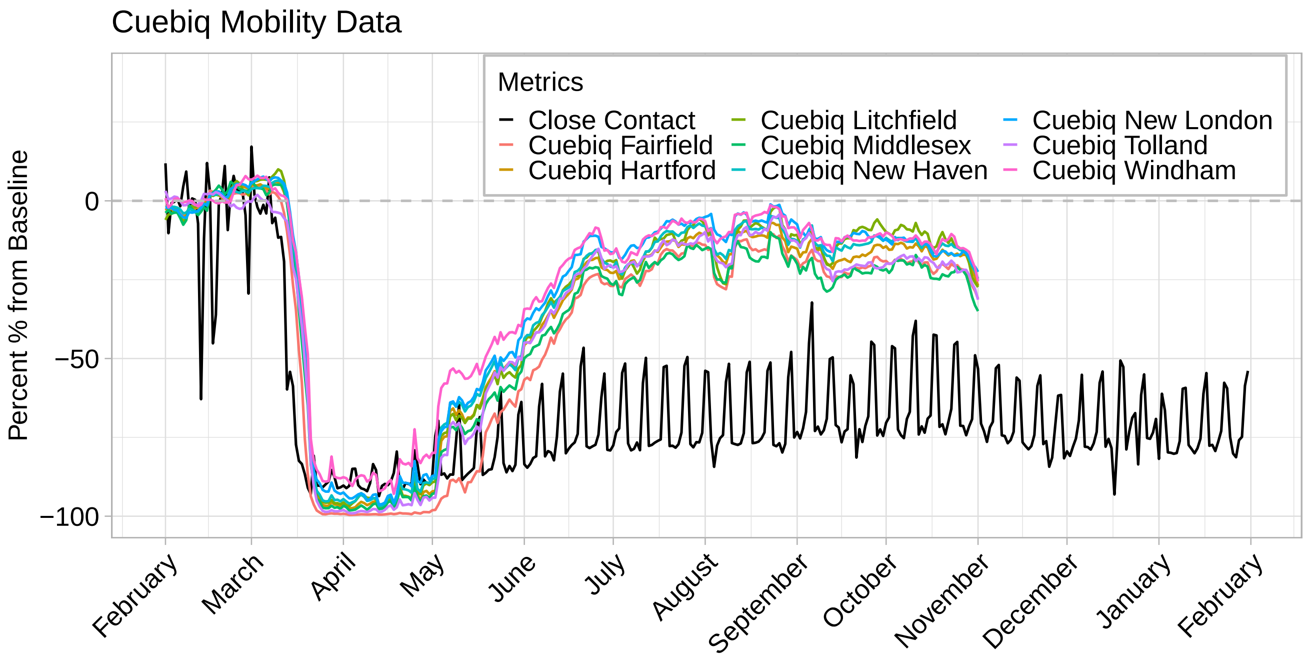

Figure 8: Comparison of Cuebiq mobility metrics to the contact rate described in this paper during February 1 – January 31, 2021. Cuebiq data available through November 1, 2020.

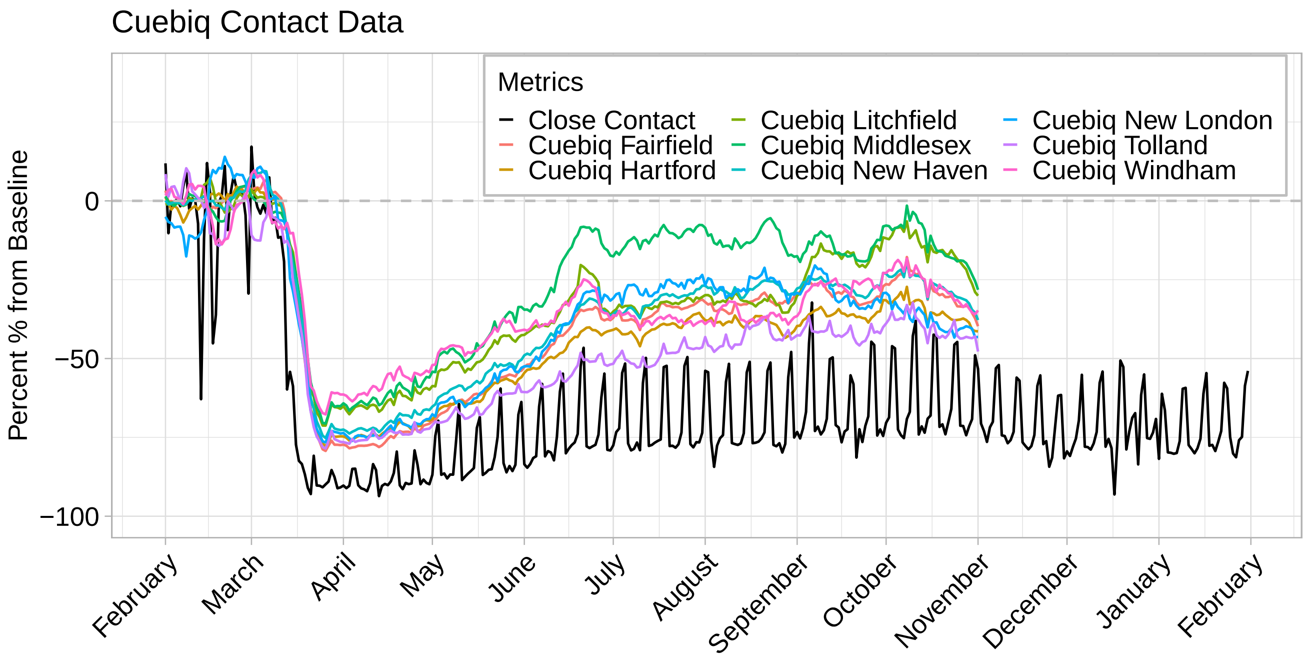

Figure 9: Comparison of the 50-foot Cuebiq contact metric to the contact rate described in this paper during February 1 – January 31, 2021. Cuebiq data available through November 1, 2020.

medRxiv preprint doi: https://doi.org/10.1101/2021.03.10.21253282; this version posted March 12, 2021. The copyright holder for this preprint (which was not certified by peer review) is the author/funder, who has granted medRxiv a license to display the preprint in perpetuity.
It is made available under a CC-BY 4.0 International license .

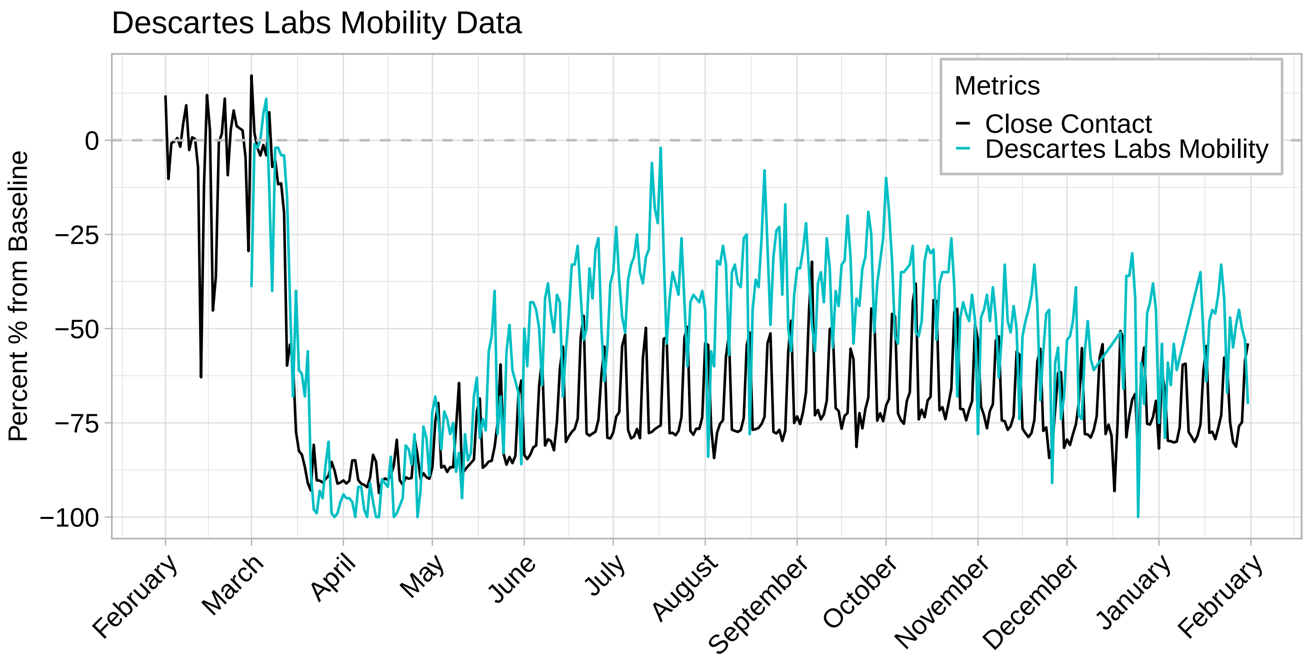

Figure 10: Comparison of Descartes Labs mobility metrics to the contact rate described in this paper during February 1 – January 31, 2021.

bility data provide the closest match to contact when presented on the percent change from February scale. In general, mobility metrics rebounded to February 2020 levels during summer 2020, and remained near this baseline or declined slightly through fall and winter 2020. Close contact, however, remained well below baseline levels through January 2021, suggesting that trends in mobility do not necessarily align with trends in close contact.

2.4 Space-time regression model results

In Table 2, we display the WAIC and posterior predictive deviance model comparison results. WAIC clearly favors the full spatio-temporal method even though the model is more complex than the two competitors as indicated by the effective number of parameters (p_{WAIC}). This improvement in model fit however, overpowers this increased penalization term, leading to an improved overall balance. In terms of prediction, the full model is again preferred and seems to predict data with similar features to the observed data better than the competing models. Global Contact fits and predicts better than No Contact, suggesting that including contact information in some form within the model leads to improvements in both areas. Overall, these results show the importance of including prior day contact information in a flexible manner in order to explain variability in COVID-19 cases. Based on these findings, we further explore results from the full spatio-temporal model.

medRxiv preprint doi: https://doi.org/10.1101/2021.03.10.21253282; this version posted March 12, 2021. The copyright holder for this preprint (which was not certified by peer review) is the author/funder, who has granted medRxiv a license to display the preprint in perpetuity.
It is made available under a CC-BY 4.0 International license .

Method	WAIC	p_{WAIC}	PP Deviance
Full Spatiotemporal	**187,849.30**	612.45	**68,177.60**
Global Contact	188,562.30	334.83	69,360.68
No Contact	188,680.40	328.88	69,535.07

Table 2: Model fit (WAIC), complexity (p_{WAIC}), and posterior predictive (ppd) deviance results from each of the competing methods.

	Relative Risk	
Variable	Mean	95% CrI
Population[a]	1.34	(1.24, 1.44)
Median Household Income (USD)[a]	0.94	(0.88, 0.99)
% Poverty[b]	0.88	(0.66, 1.15)
% White, Non-Hispanic[b]	0.89	(0.81, 0.99)
% Some College Education[b]	0.95	(0.67, 1.31)
% $\geq$ 65 Years[b]	0.77	(0.58, 0.98)

Table 3: Regression parameter posterior inference (posterior means and quantile-based 95% credible intervals) from the full spatio-temporal model. (a) 10,000 increase; (b) 10% increase.

In Table 3, we display posterior inference for the non-contact regression parameters (β_j), exponentiated for a relative risk interpretation. Results suggest that towns with increased population have more cases on average, and that towns with higher median household income and a larger proportion of residents that are non-Hispanic White and $\geq$ 65 years have fewer cases. None of the other included spatially-varying covariates had 95% credible intervals that excluded one.

In Figure 11, we show the estimated global lag parameters, $\boldsymbol{\theta}$, and 95% credible intervals across the different lag days from the full spatio-temporal version of the model. The figure suggests that increased contact on prior days 3-7 is associated with increased current day COVID-19 cases.

2.5 Transmission model predicted cases and estimated infections for each town

Figure 12 through 33 show contact, projected infections, cases, and cumulative incidence for each of the 169 towns in Connecticut.

medRxiv preprint doi: https://doi.org/10.1101/2021.03.10.21253282; this version posted March 12, 2021. The copyright holder for this preprint (which was not certified by peer review) is the author/funder, who has granted medRxiv a license to display the preprint in perpetuity.
It is made available under a CC-BY 4.0 International license .

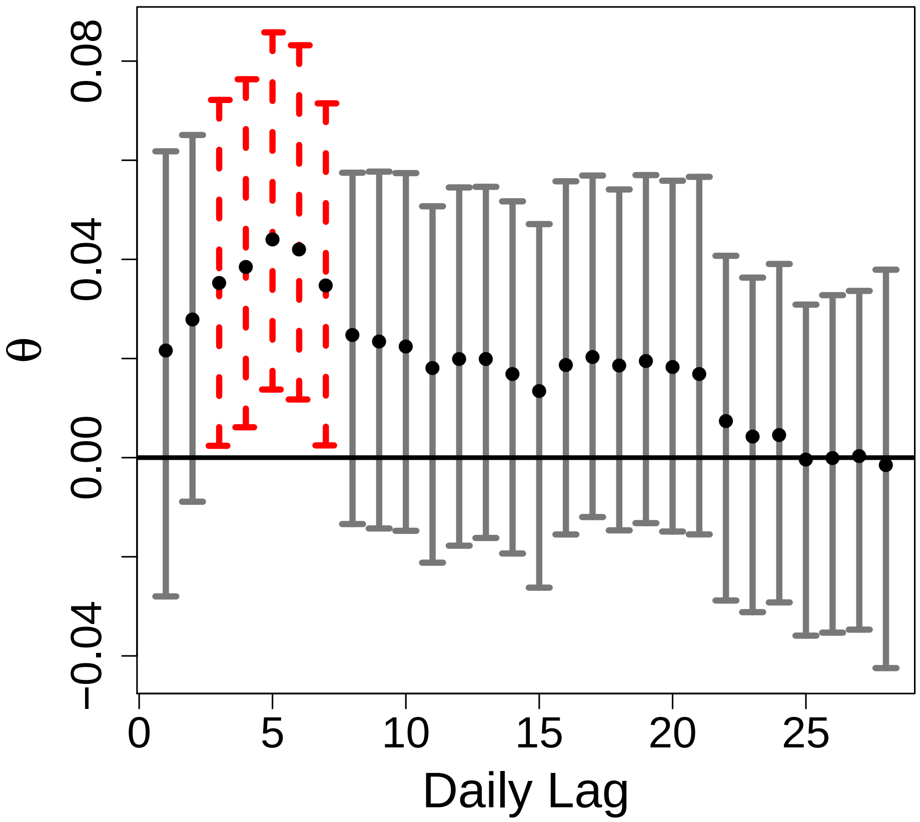

Figure 11: Estimated daily lag regression parameters (and 95% credible intervals) describing associations between contact and current day COVID-19 cases. Red bars indicate that the credible intervals exclude zero.

medRxiv preprint doi: https://doi.org/10.1101/2021.03.10.21253282; this version posted March 12, 2021. The copyright holder for this preprint (which was not certified by peer review) is the author/funder, who has granted medRxiv a license to display the preprint in perpetuity.
It is made available under a CC-BY 4.0 International license .

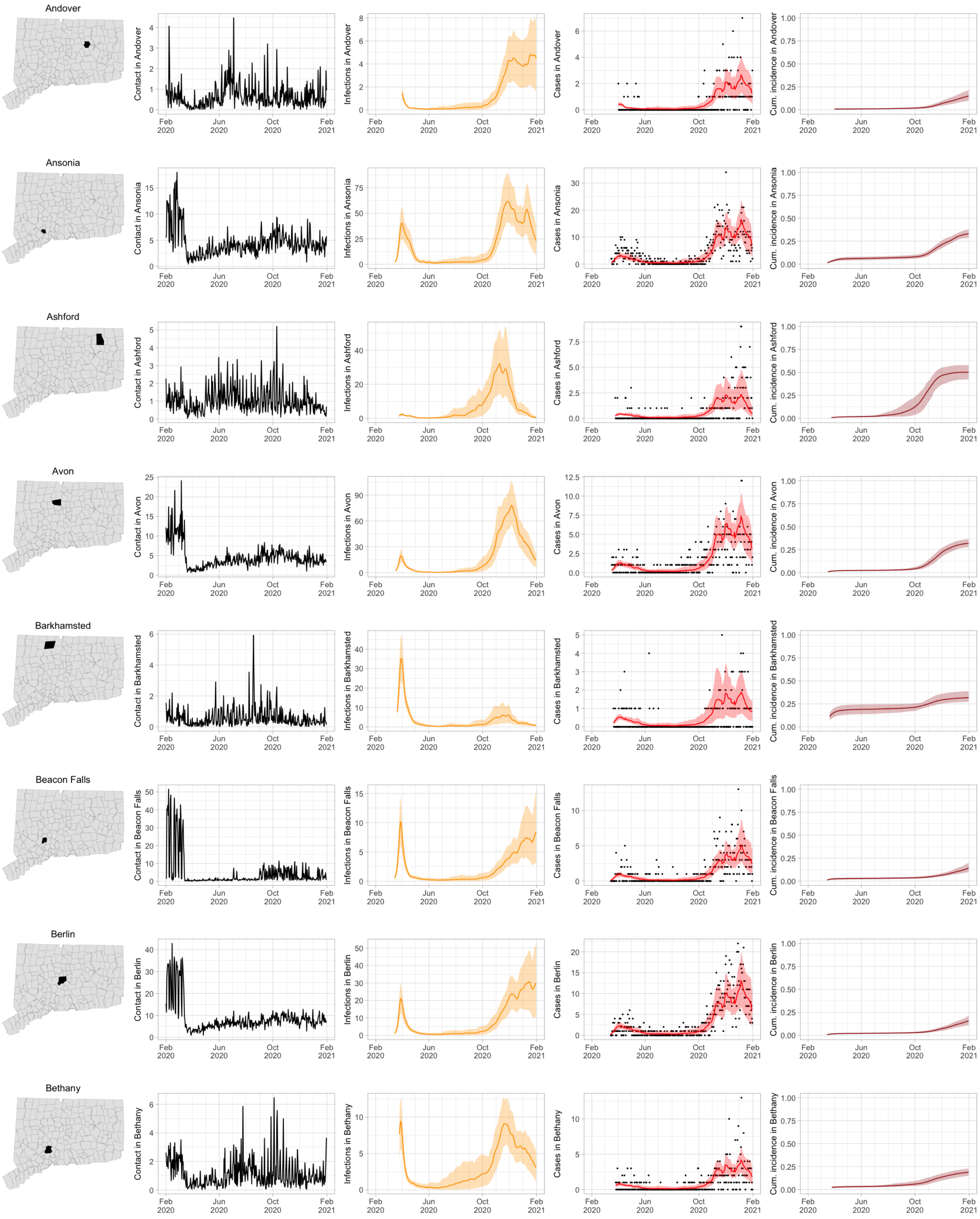

Figure 12: Contact for Connecticut towns and fitted SEIR model predictions with 95% uncertainty intervals.

medRxiv preprint doi: https://doi.org/10.1101/2021.03.10.21253282; this version posted March 12, 2021. The copyright holder for this preprint (which was not certified by peer review) is the author/funder, who has granted medRxiv a license to display the preprint in perpetuity.
It is made available under a CC-BY 4.0 International license .

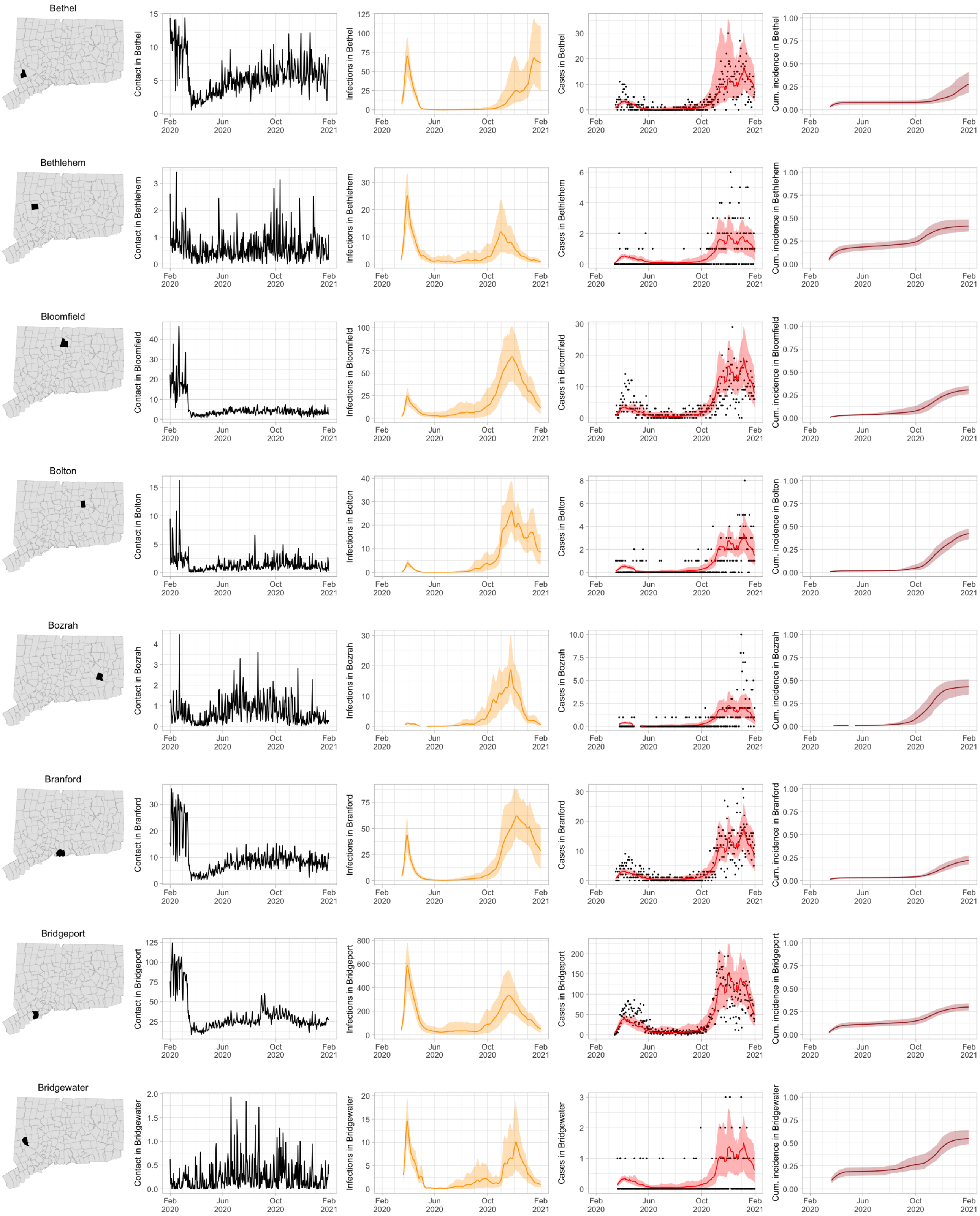

Figure 13: Contact for Connecticut towns and fitted SEIR model predictions with 95% uncertainty intervals.

medRxiv preprint doi: https://doi.org/10.1101/2021.03.10.21253282; this version posted March 12, 2021. The copyright holder for this preprint (which was not certified by peer review) is the author/funder, who has granted medRxiv a license to display the preprint in perpetuity.
It is made available under a CC-BY 4.0 International license .

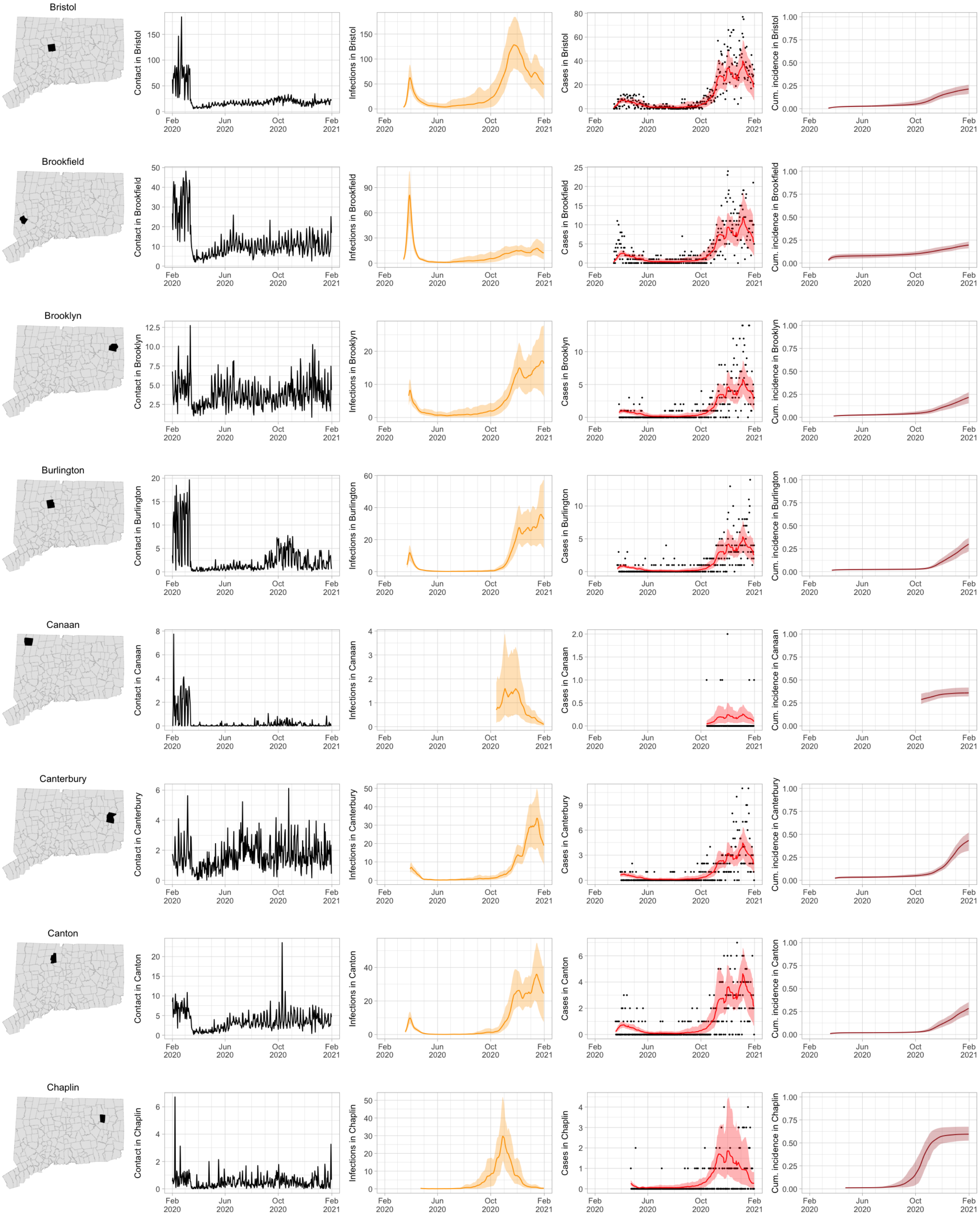

Figure 14: Contact for Connecticut towns and fitted SEIR model predictions with 95% uncertainty intervals.

medRxiv preprint doi: https://doi.org/10.1101/2021.03.10.21253282; this version posted March 12, 2021. The copyright holder for this preprint **(which was not certified by peer review)** is the author/funder, who has granted medRxiv a license to display the preprint in perpetuity.
It is made available under a CC-BY 4.0 International license .

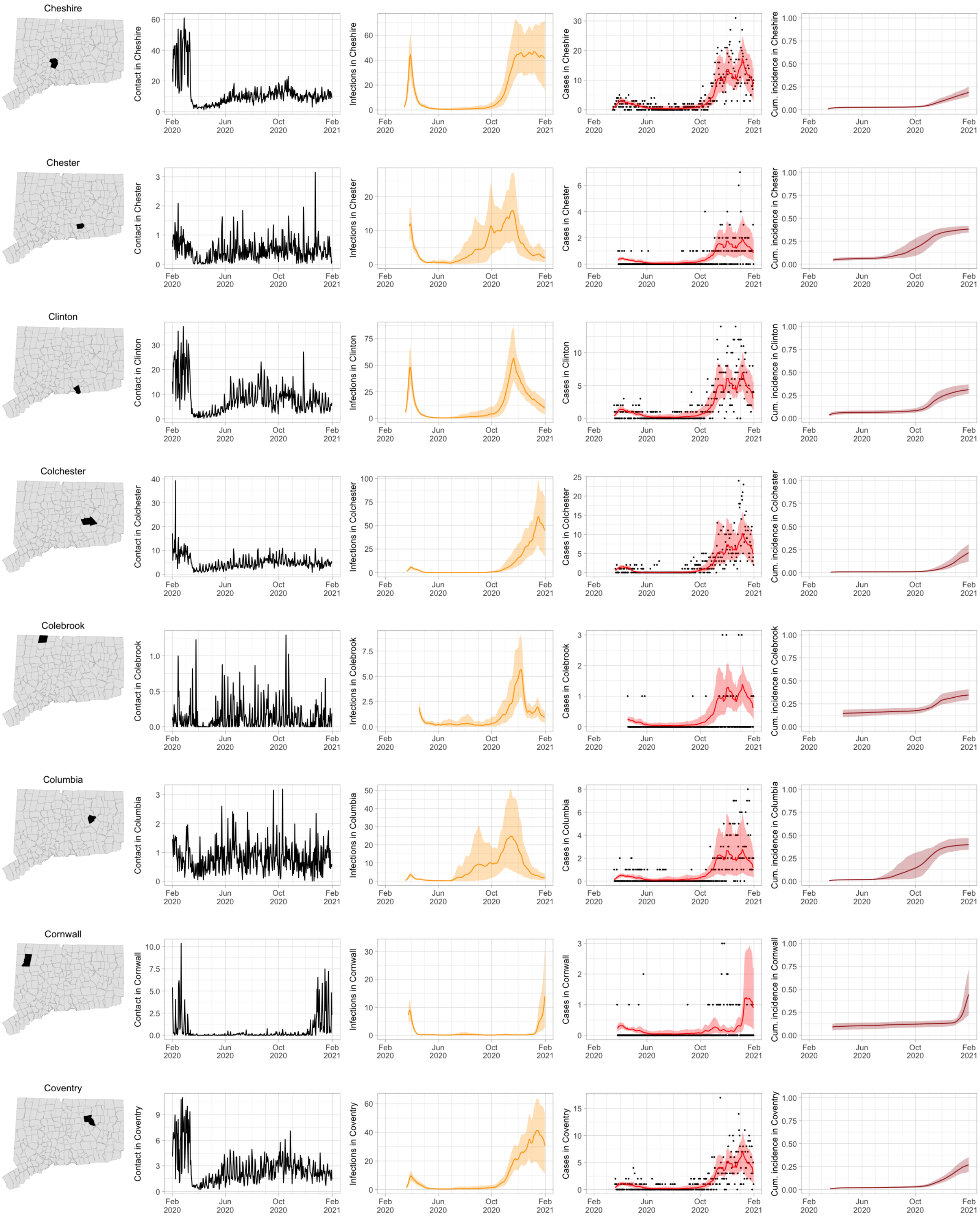

Figure 15: Contact for Connecticut towns and fitted SEIR model predictions with 95% uncertainty intervals.

medRxiv preprint doi: https://doi.org/10.1101/2021.03.10.21253282; this version posted March 12, 2021. The copyright holder for this preprint (which was not certified by peer review) is the author/funder, who has granted medRxiv a license to display the preprint in perpetuity.
It is made available under a CC-BY 4.0 International license .

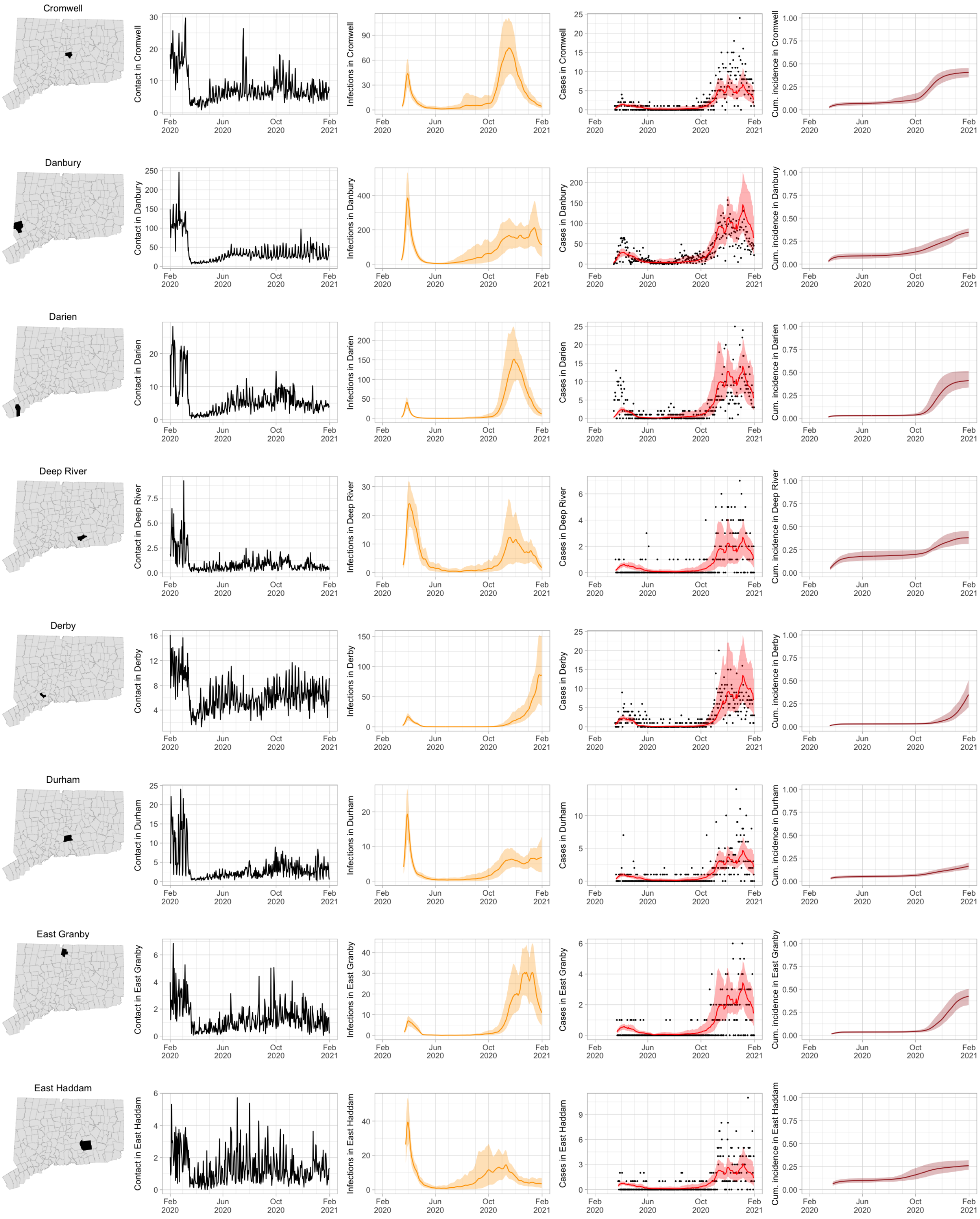

Figure 16: Contact for Connecticut towns and fitted SEIR model predictions with 95% uncertainty intervals.

medRxiv preprint doi: https://doi.org/10.1101/2021.03.10.21253282; this version posted March 12, 2021. The copyright holder for this preprint (which was not certified by peer review) is the author/funder, who has granted medRxiv a license to display the preprint in perpetuity.
It is made available under a CC-BY 4.0 International license .

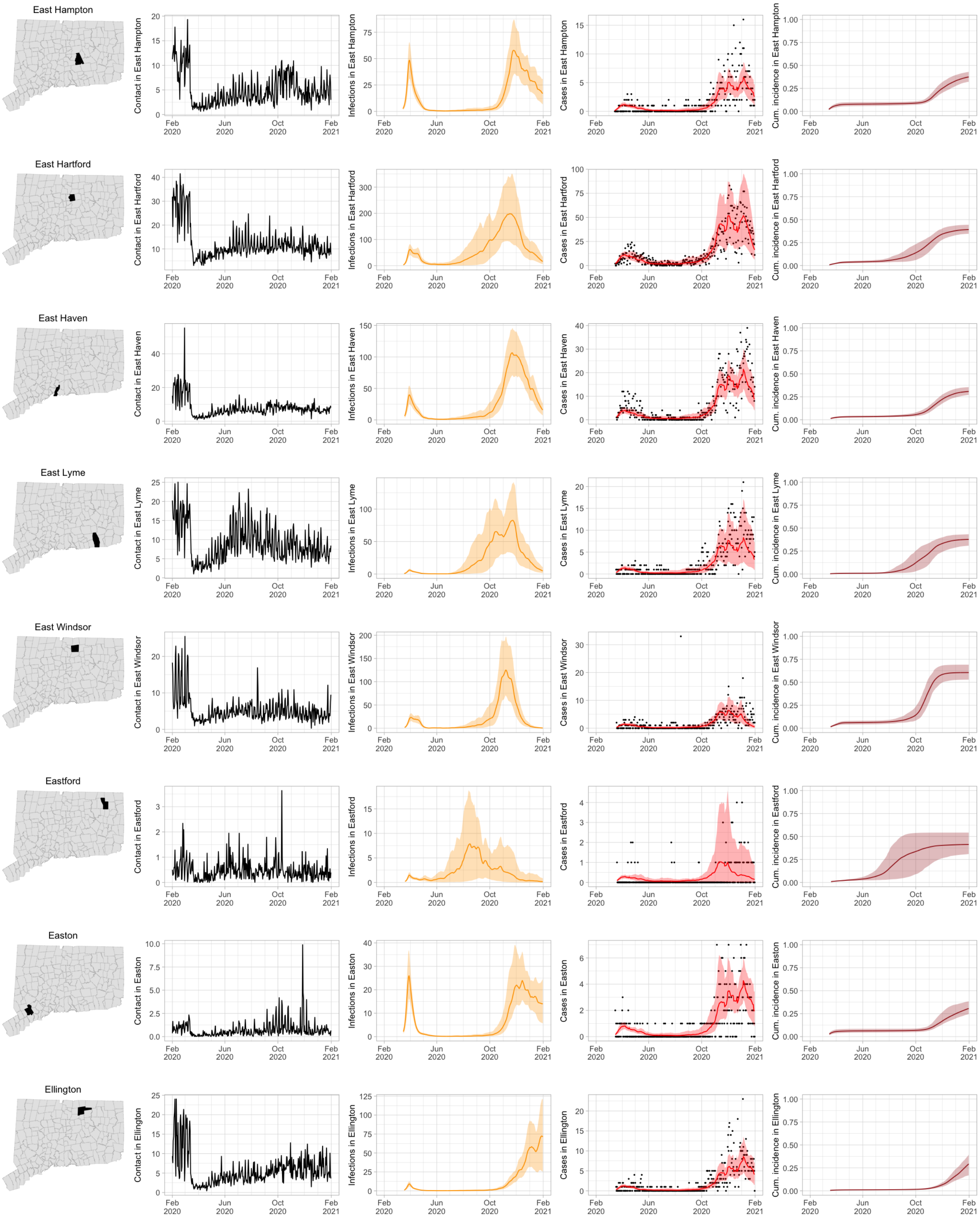

Figure 17: Contact for Connecticut towns and fitted SEIR model predictions with 95% uncertainty intervals.

medRxiv preprint doi: https://doi.org/10.1101/2021.03.10.21253282; this version posted March 12, 2021. The copyright holder for this preprint (which was not certified by peer review) is the author/funder, who has granted medRxiv a license to display the preprint in perpetuity.
It is made available under a CC-BY 4.0 International license .

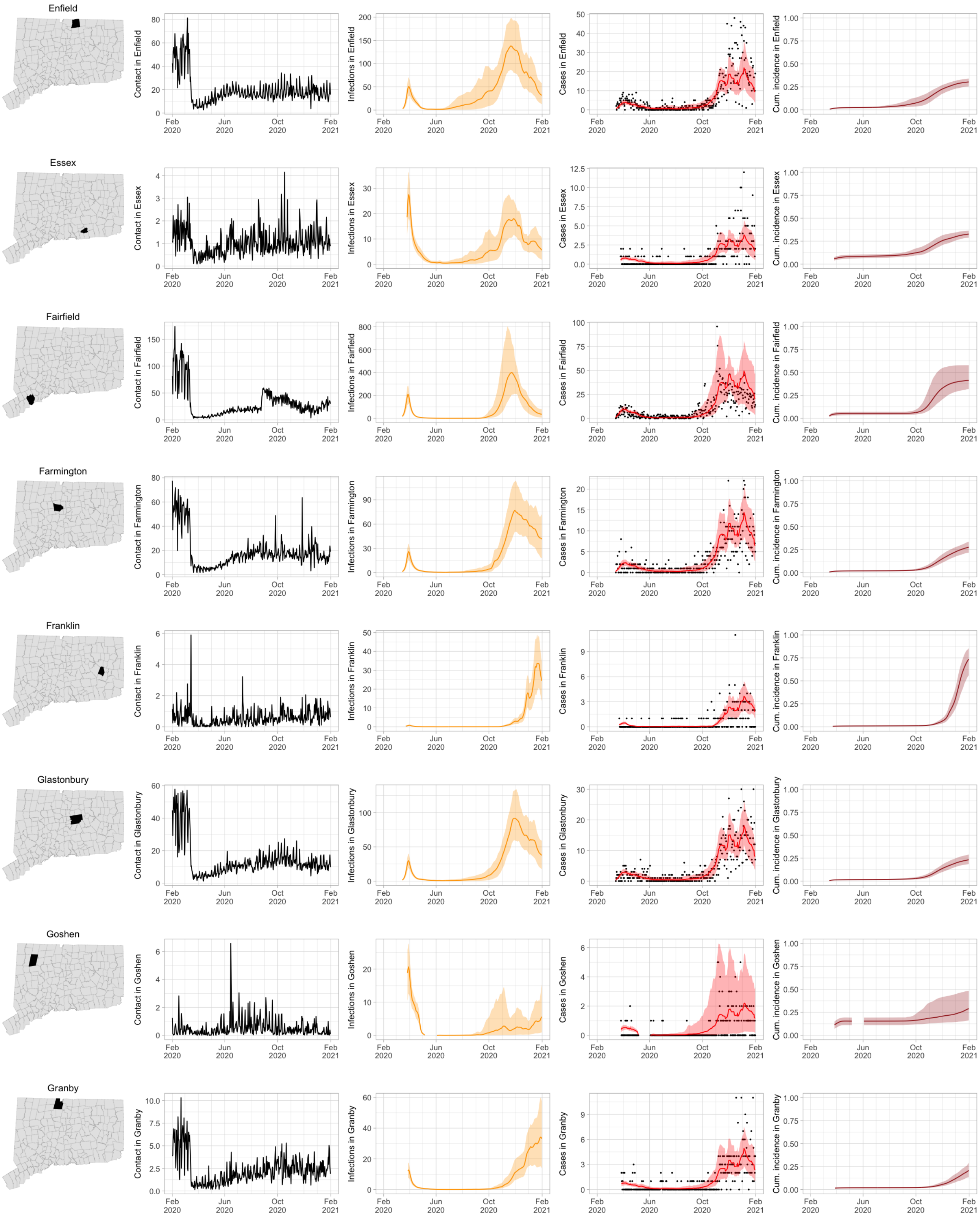

Figure 18: Contact for Connecticut towns and fitted SEIR model predictions with 95% uncertainty intervals.

medRxiv preprint doi: https://doi.org/10.1101/2021.03.10.21253282; this version posted March 12, 2021. The copyright holder for this preprint **(which was not certified by peer review)** is the author/funder, who has granted medRxiv a license to display the preprint in perpetuity.
It is made available under a CC-BY 4.0 International license .

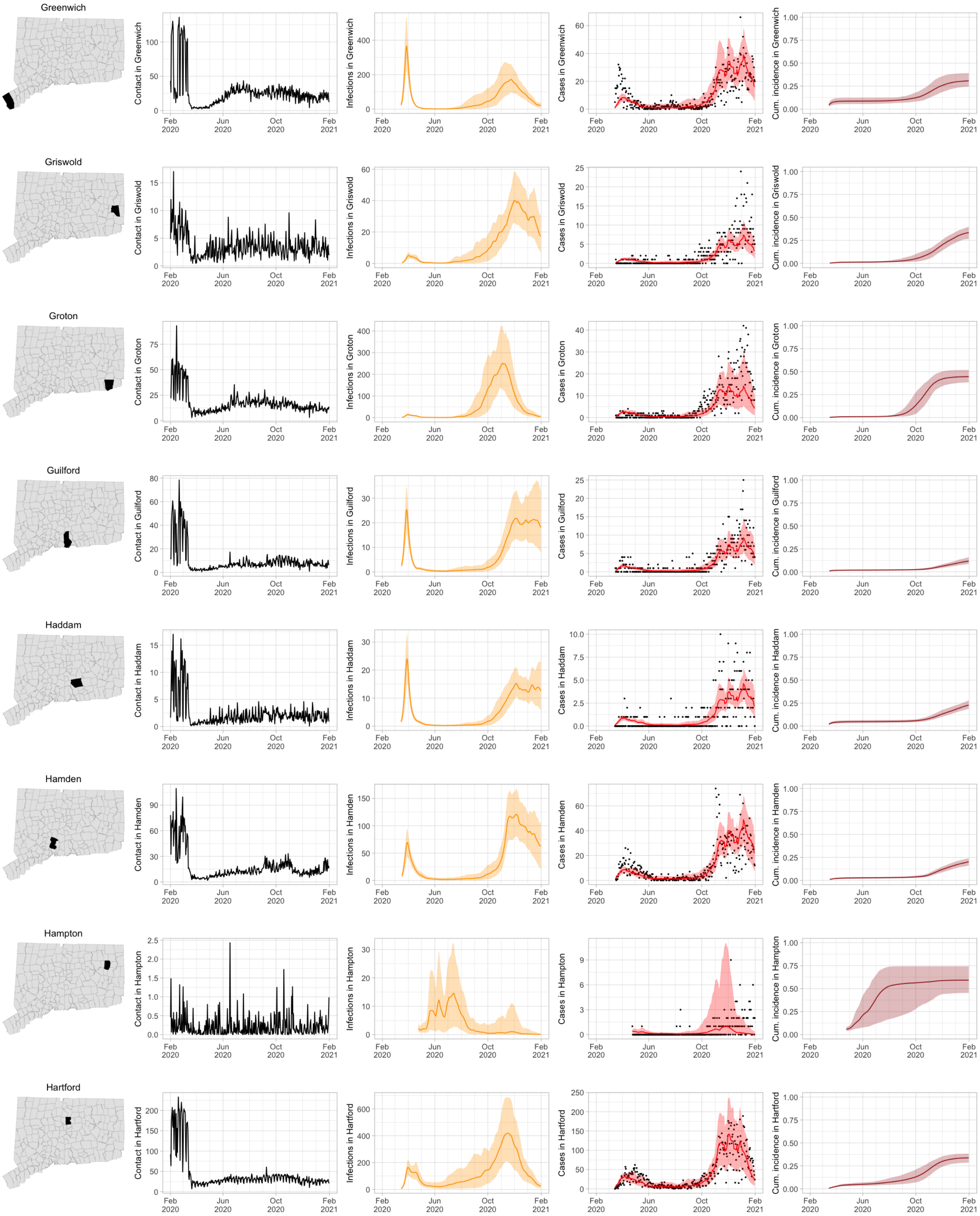

Figure 19: Contact for Connecticut towns and fitted SEIR model predictions with 95% uncertainty intervals.

medRxiv preprint doi: https://doi.org/10.1101/2021.03.10.21253282; this version posted March 12, 2021. The copyright holder for this preprint (which was not certified by peer review) is the author/funder, who has granted medRxiv a license to display the preprint in perpetuity.
It is made available under a CC-BY 4.0 International license .

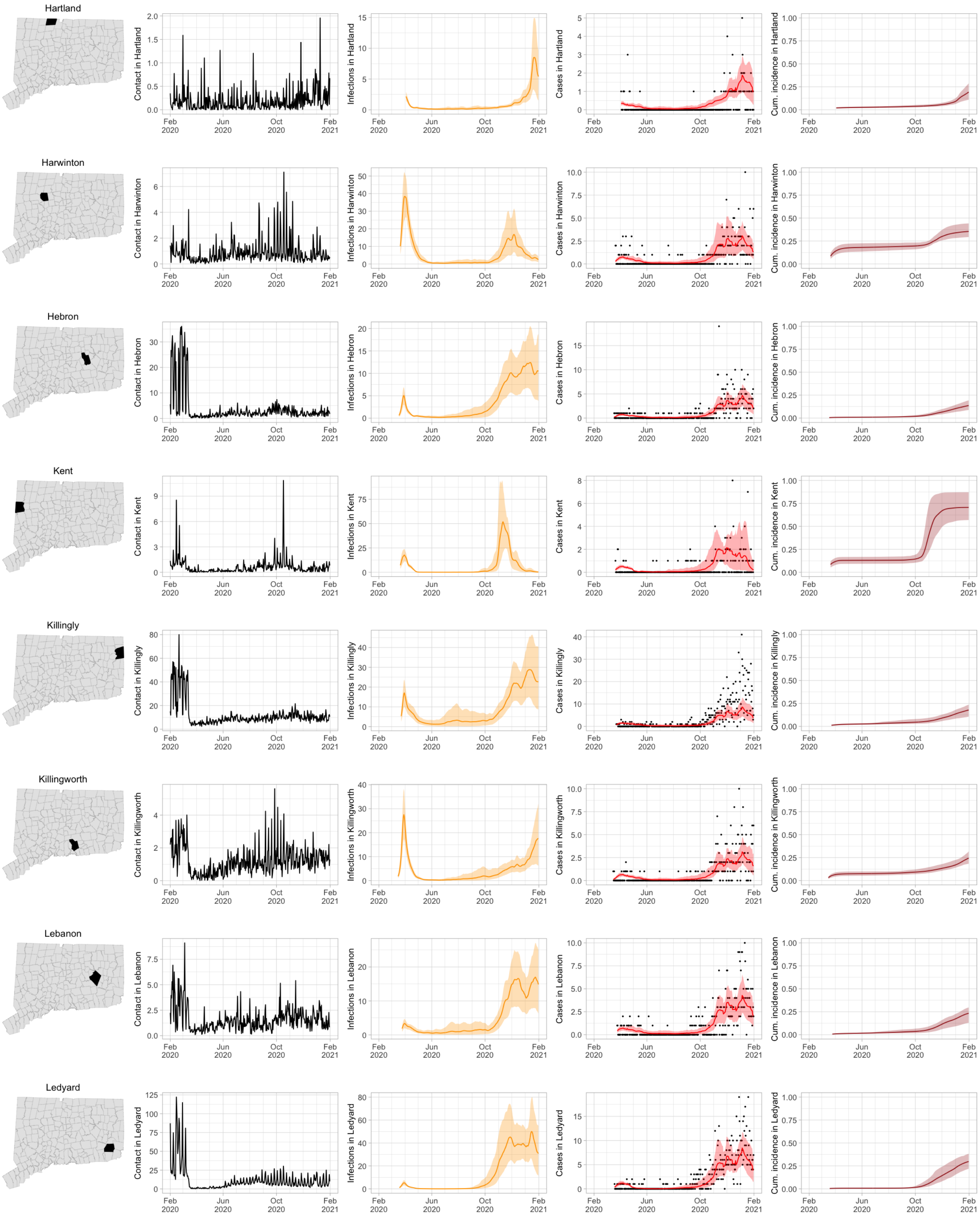

Figure 20: Contact for Connecticut towns and fitted SEIR model predictions with 95% uncertainty intervals.

medRxiv preprint doi: https://doi.org/10.1101/2021.03.10.21253282; this version posted March 12, 2021. The copyright holder for this preprint (which was not certified by peer review) is the author/funder, who has granted medRxiv a license to display the preprint in perpetuity.
It is made available under a CC-BY 4.0 International license .

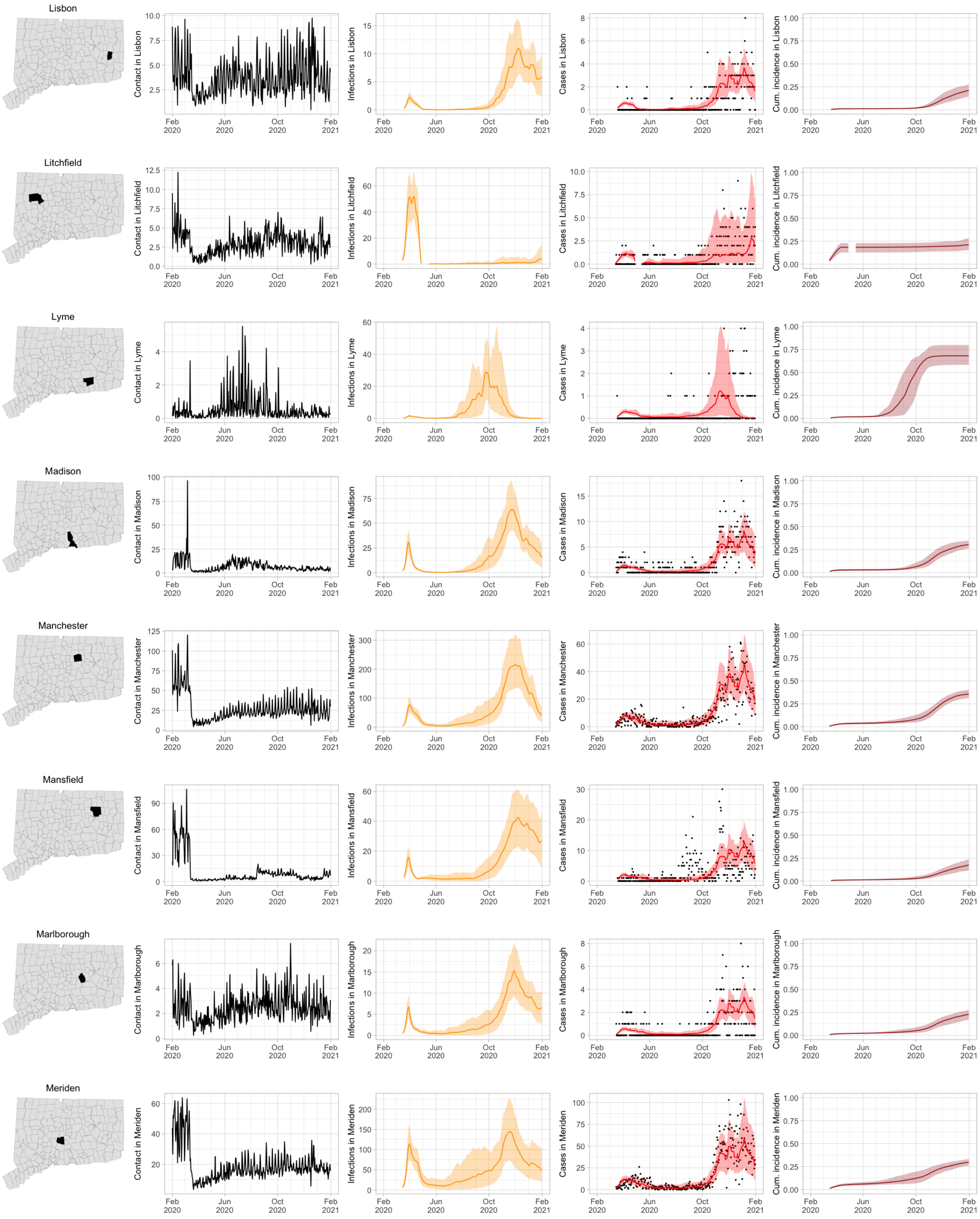

Figure 21: Contact for Connecticut towns and fitted SEIR model predictions with 95% uncertainty intervals.

medRxiv preprint doi: https://doi.org/10.1101/2021.03.10.21253282; this version posted March 12, 2021. The copyright holder for this preprint (which was not certified by peer review) is the author/funder, who has granted medRxiv a license to display the preprint in perpetuity.
It is made available under a CC-BY 4.0 International license .

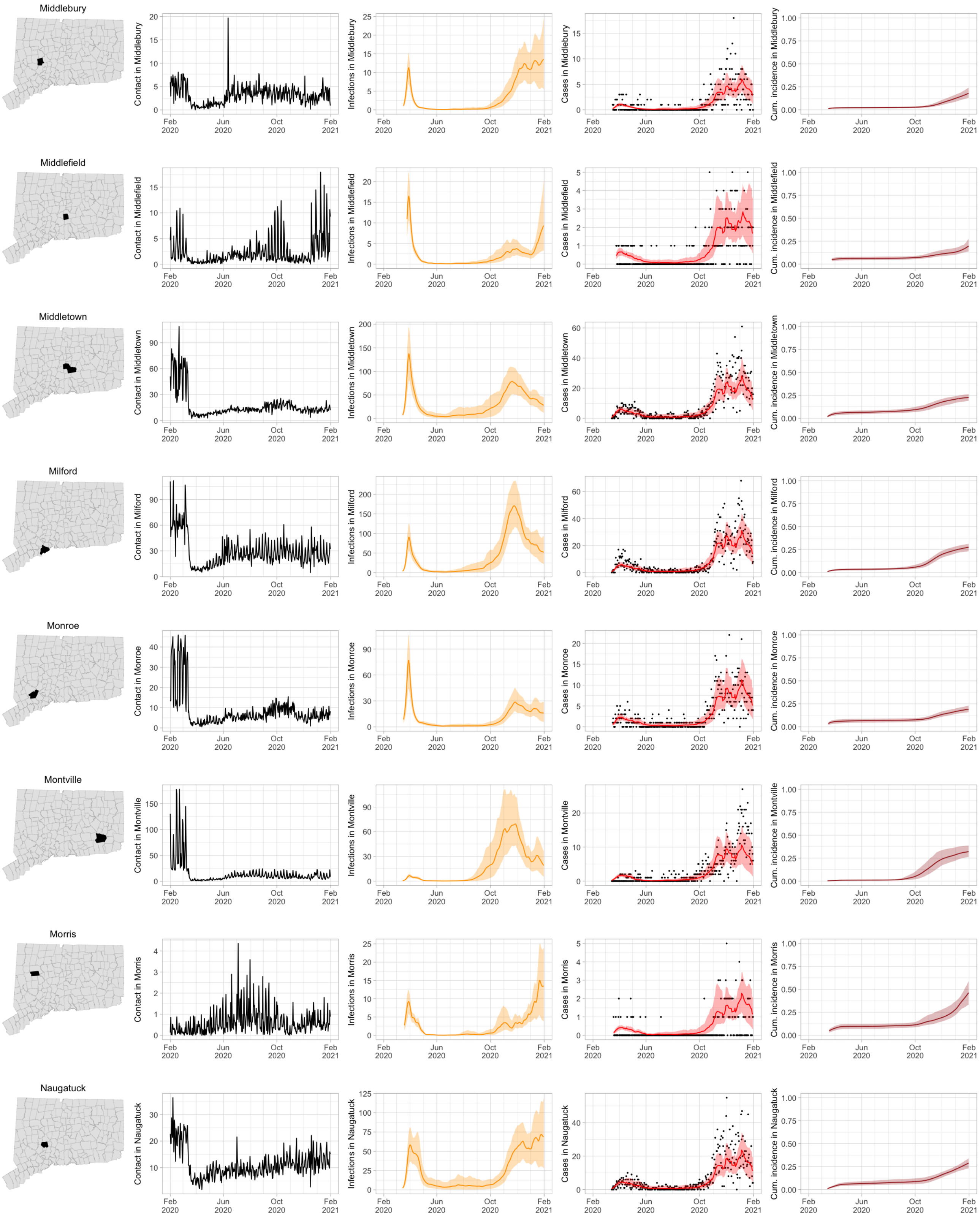

Figure 22: Contact for Connecticut towns and fitted SEIR model predictions with 95% uncertainty intervals.

medRxiv preprint doi: https://doi.org/10.1101/2021.03.10.21253282; this version posted March 12, 2021. The copyright holder for this preprint (which was not certified by peer review) is the author/funder, who has granted medRxiv a license to display the preprint in perpetuity.
It is made available under a CC-BY 4.0 International license .

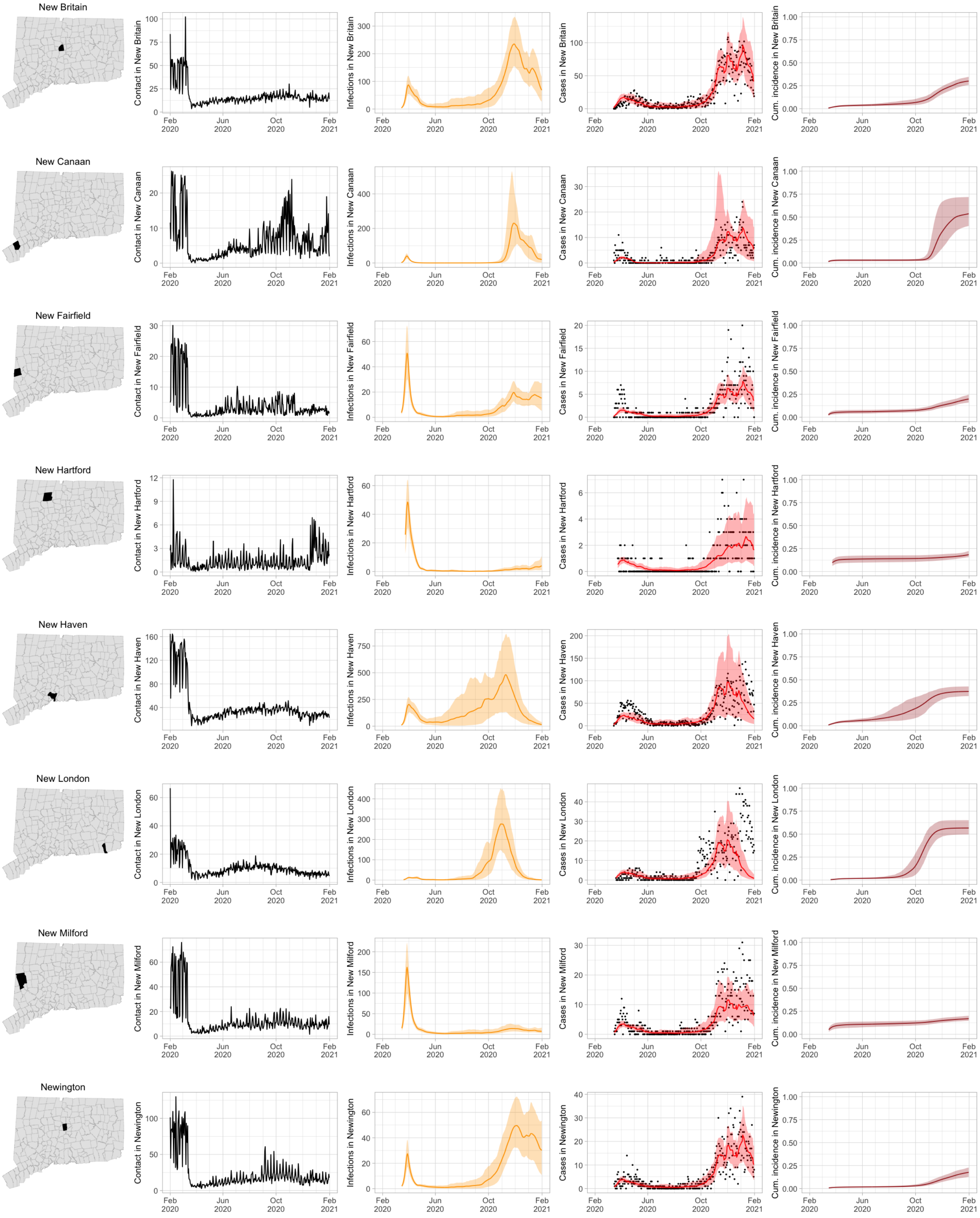

Figure 23: Contact for Connecticut towns and fitted SEIR model predictions with 95% uncertainty intervals.

medRxiv preprint doi: https://doi.org/10.1101/2021.03.10.21253282; this version posted March 12, 2021. The copyright holder for this preprint (which was not certified by peer review) is the author/funder, who has granted medRxiv a license to display the preprint in perpetuity.
It is made available under a CC-BY 4.0 International license .

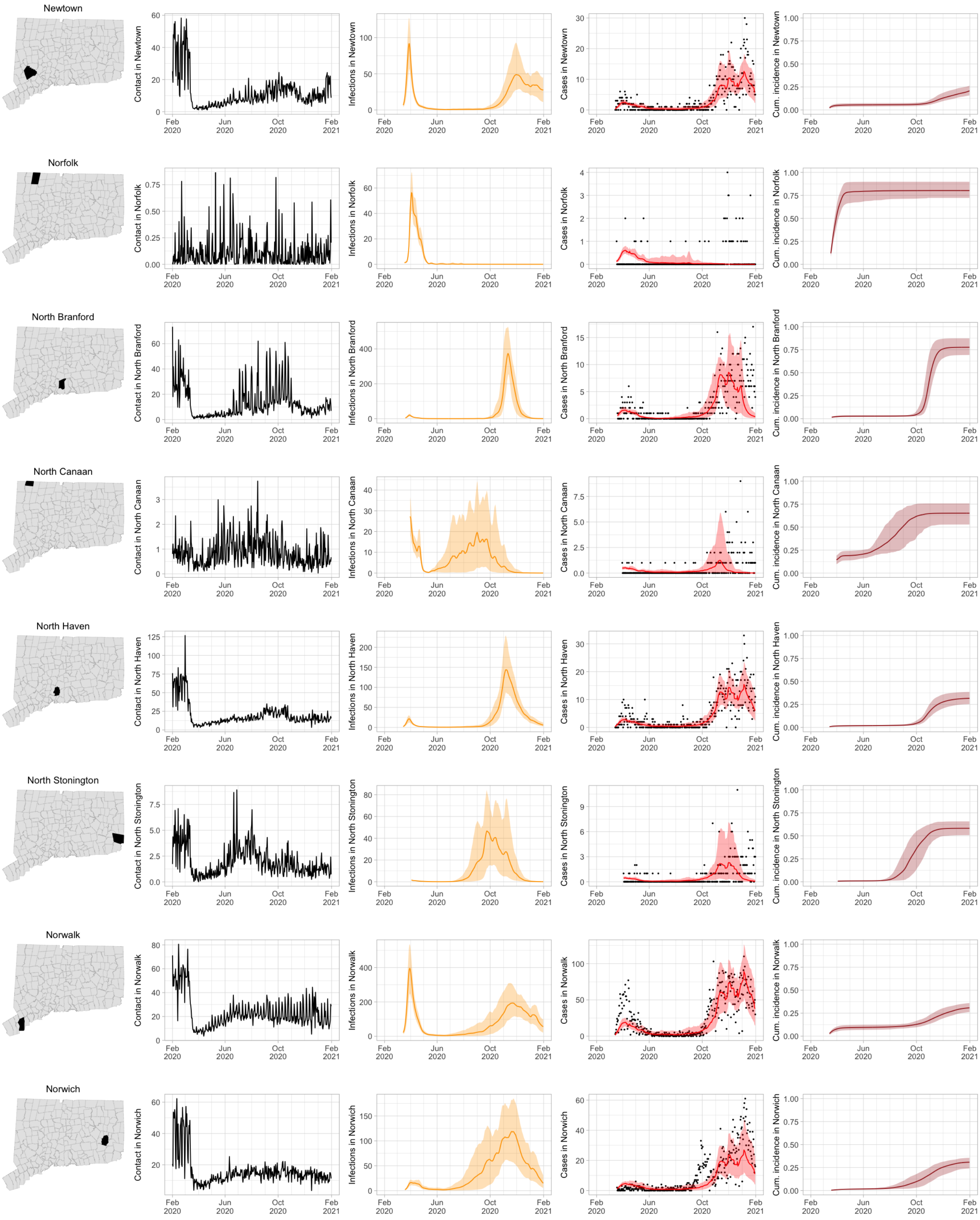

Figure 24: Contact for Connecticut towns and fitted SEIR model predictions with 95% uncertainty intervals.

medRxiv preprint doi: https://doi.org/10.1101/2021.03.10.21253282; this version posted March 12, 2021. The copyright holder for this preprint (which was not certified by peer review) is the author/funder, who has granted medRxiv a license to display the preprint in perpetuity.
It is made available under a CC-BY 4.0 International license .

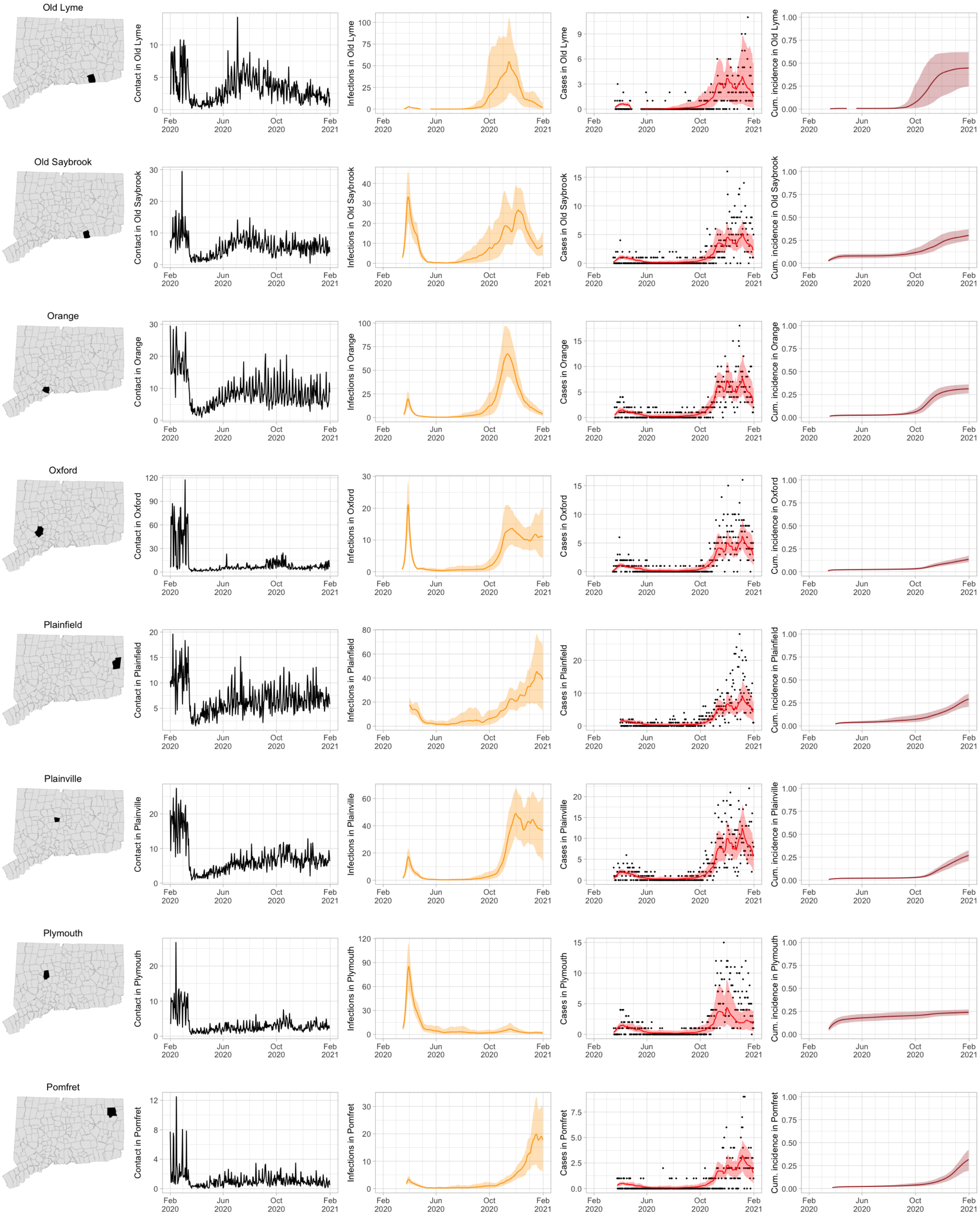

Figure 25: Contact for Connecticut towns and fitted SEIR model predictions with 95% uncertainty intervals.

medRxiv preprint doi: https://doi.org/10.1101/2021.03.10.21253282; this version posted March 12, 2021. The copyright holder for this preprint **(which was not certified by peer review)** is the author/funder, who has granted medRxiv a license to display the preprint in perpetuity.
It is made available under a CC-BY 4.0 International license .

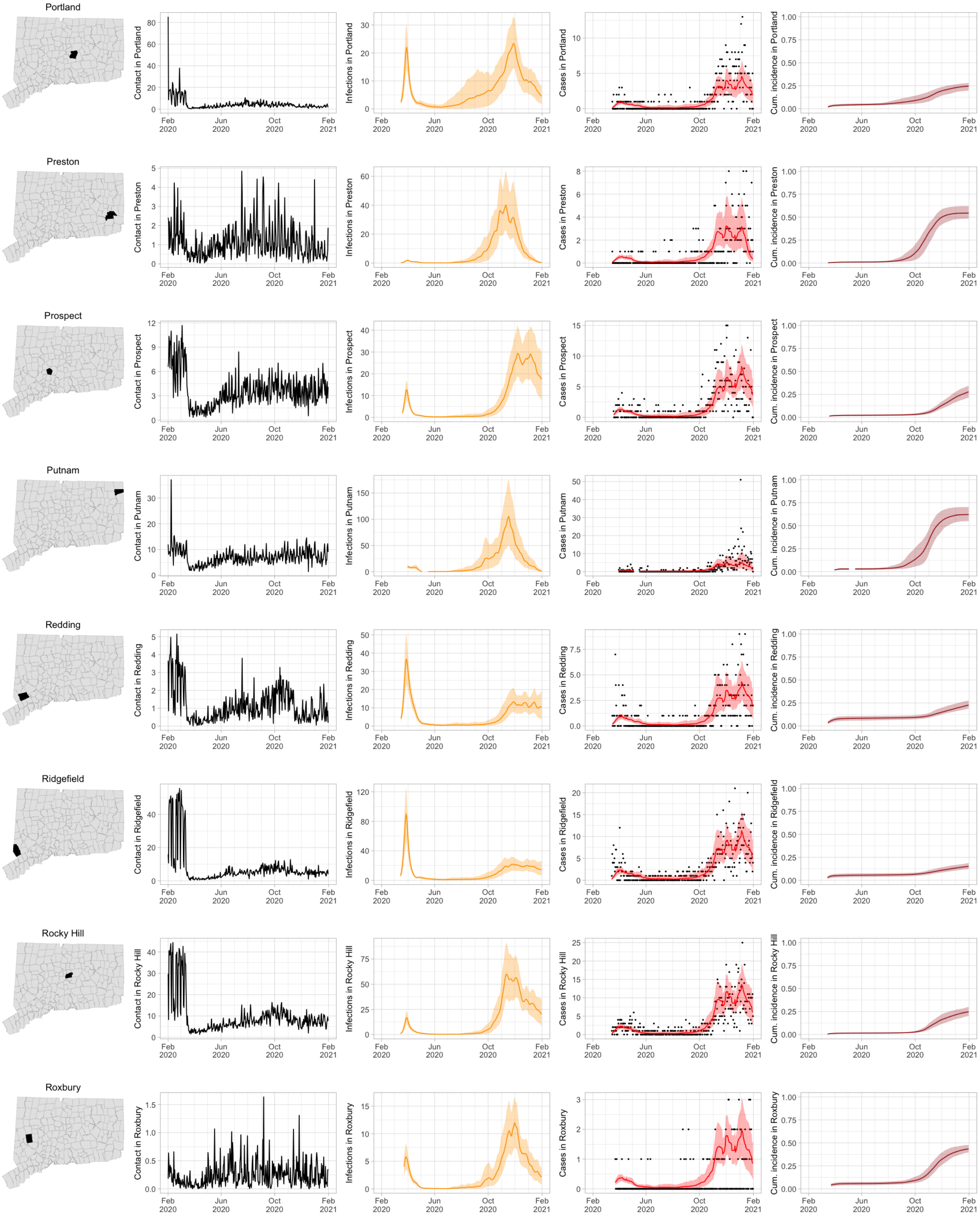

Figure 26: Contact for Connecticut towns and fitted SEIR model predictions with 95% uncertainty intervals.

medRxiv preprint doi: https://doi.org/10.1101/2021.03.10.21253282; this version posted March 12, 2021. The copyright holder for this preprint (which was not certified by peer review) is the author/funder, who has granted medRxiv a license to display the preprint in perpetuity.
It is made available under a CC-BY 4.0 International license .

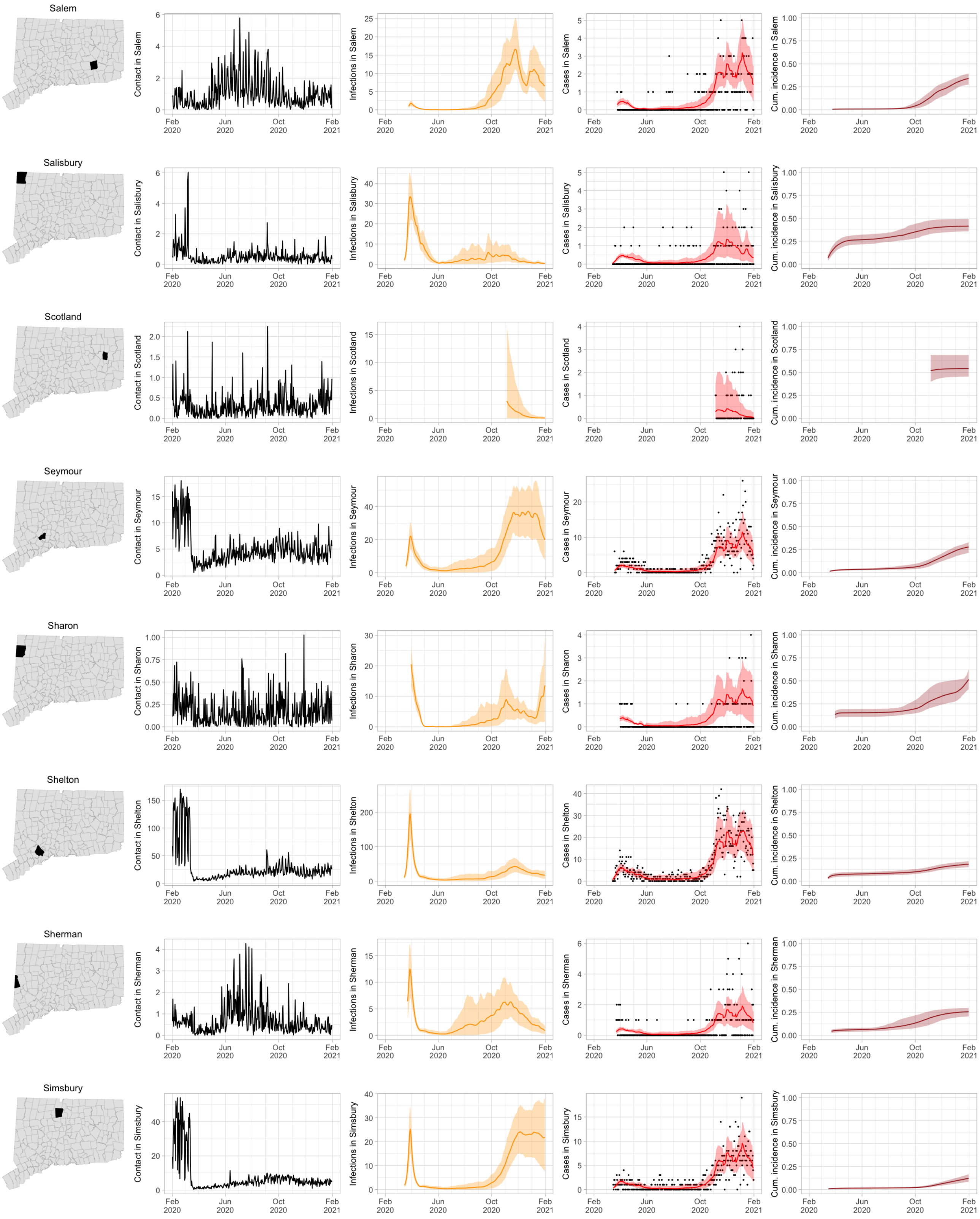

Figure 27: Contact for Connecticut towns and fitted SEIR model predictions with 95% uncertainty intervals.

medRxiv preprint doi: https://doi.org/10.1101/2021.03.10.21253282; this version posted March 12, 2021. The copyright holder for this preprint (which was not certified by peer review) is the author/funder, who has granted medRxiv a license to display the preprint in perpetuity.
It is made available under a CC-BY 4.0 International license .

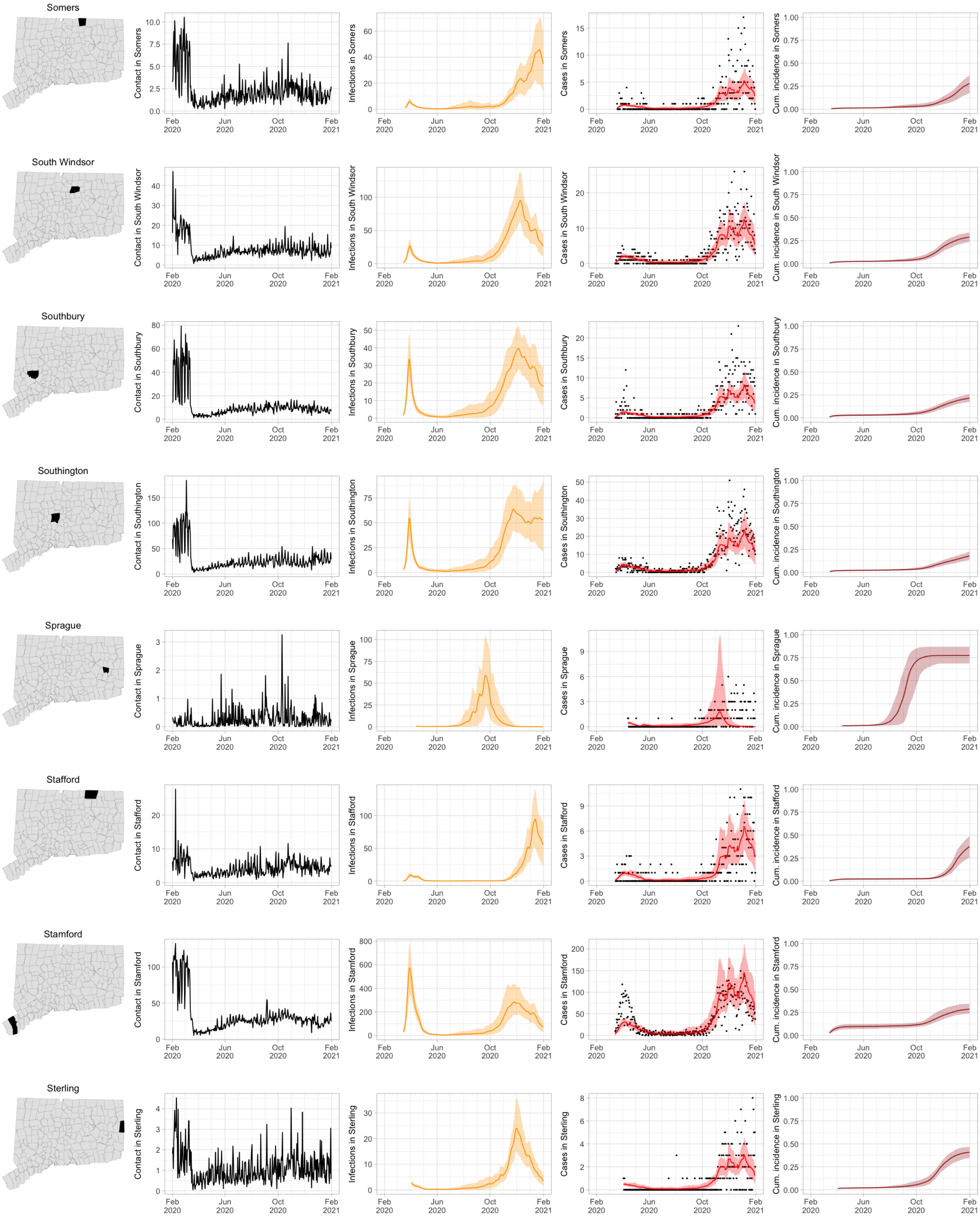

Figure 28: Contact for Connecticut towns and fitted SEIR model predictions with 95% uncertainty intervals.

medRxiv preprint doi: https://doi.org/10.1101/2021.03.10.21253282; this version posted March 12, 2021. The copyright holder for this preprint (which was not certified by peer review) is the author/funder, who has granted medRxiv a license to display the preprint in perpetuity.
It is made available under a CC-BY 4.0 International license .

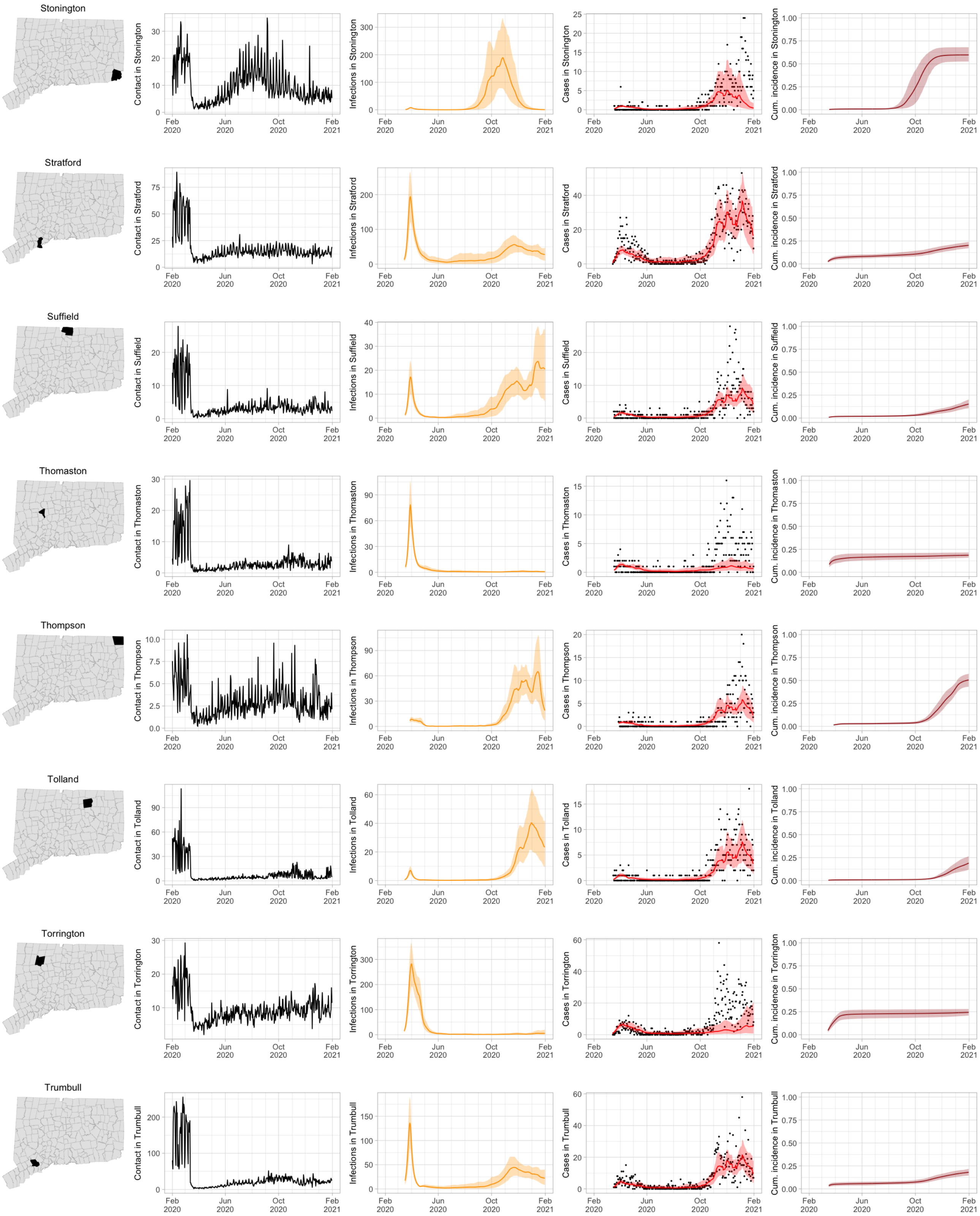

Figure 29: Contact for Connecticut towns and fitted SEIR model predictions with 95% uncertainty intervals.

medRxiv preprint doi: https://doi.org/10.1101/2021.03.10.21253282; this version posted March 12, 2021. The copyright holder for this preprint (which was not certified by peer review) is the author/funder, who has granted medRxiv a license to display the preprint in perpetuity.
It is made available under a CC-BY 4.0 International license .

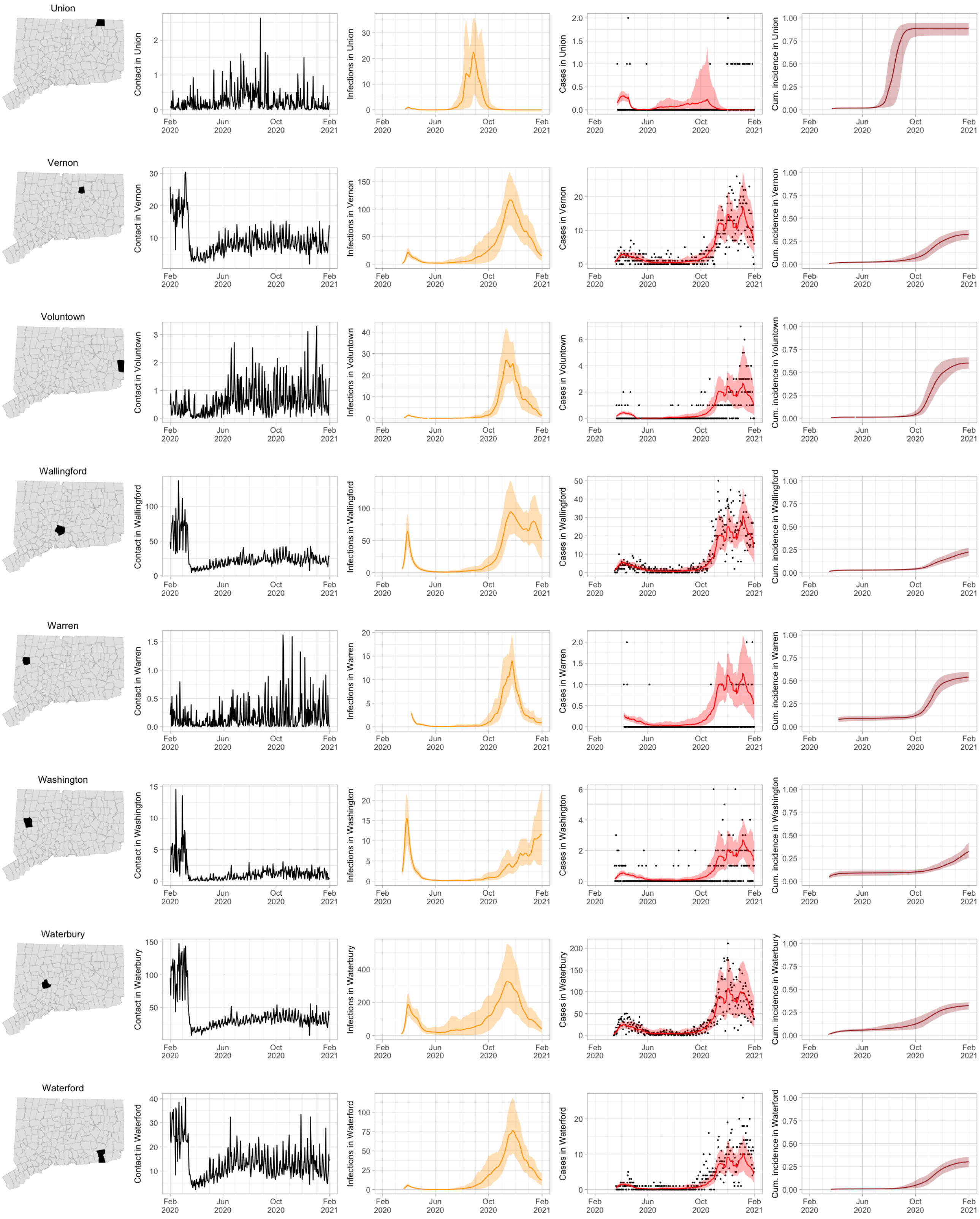

Figure 30: Contact for Connecticut towns and fitted SEIR model predictions with 95% uncertainty intervals.

medRxiv preprint doi: https://doi.org/10.1101/2021.03.10.21253282; this version posted March 12, 2021. The copyright holder for this preprint (which was not certified by peer review) is the author/funder, who has granted medRxiv a license to display the preprint in perpetuity.
It is made available under a CC-BY 4.0 International license .

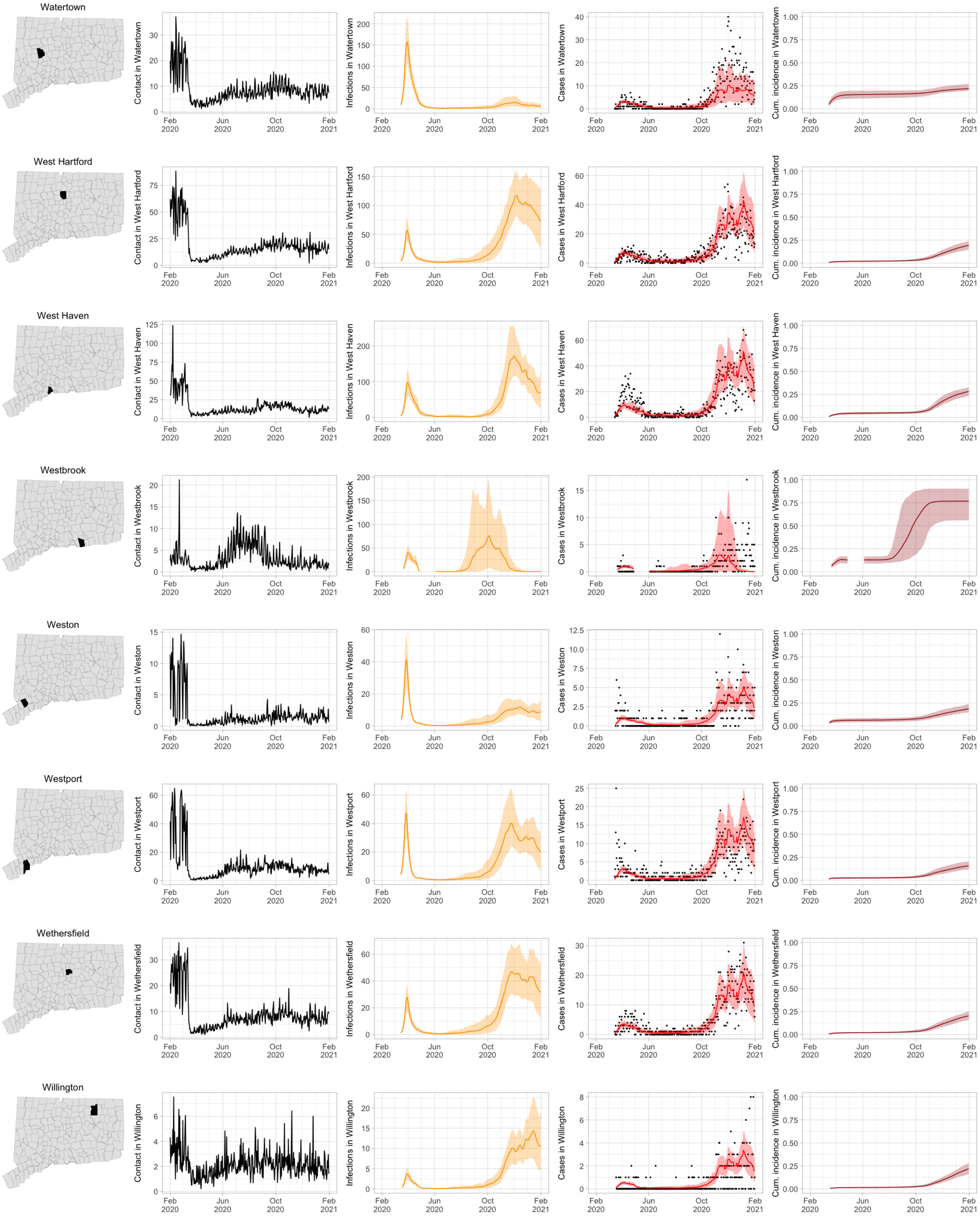

Figure 31: Contact for Connecticut towns and fitted SEIR model predictions with 95% uncertainty intervals.

medRxiv preprint doi: https://doi.org/10.1101/2021.03.10.21253282; this version posted March 12, 2021. The copyright holder for this preprint (which was not certified by peer review) is the author/funder, who has granted medRxiv a license to display the preprint in perpetuity.
It is made available under a CC-BY 4.0 International license .

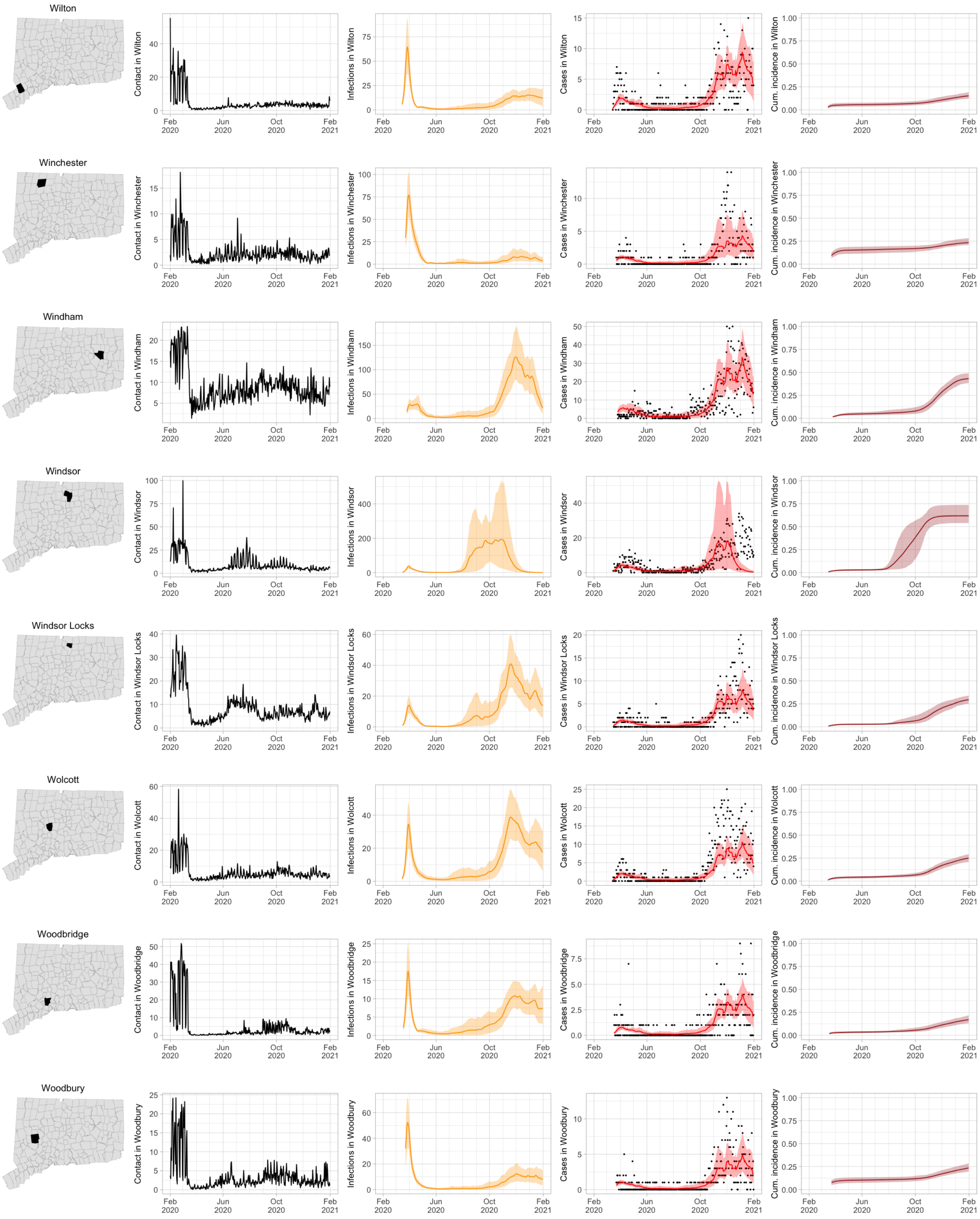

Figure 32: Contact for Connecticut towns and fitted SEIR model predictions with 95% uncertainty intervals.

medRxiv preprint doi: https://doi.org/10.1101/2021.03.10.21253282; this version posted March 12, 2021. The copyright holder for this preprint (which was not certified by peer review) is the author/funder, who has granted medRxiv a license to display the preprint in perpetuity.
It is made available under a CC-BY 4.0 International license .

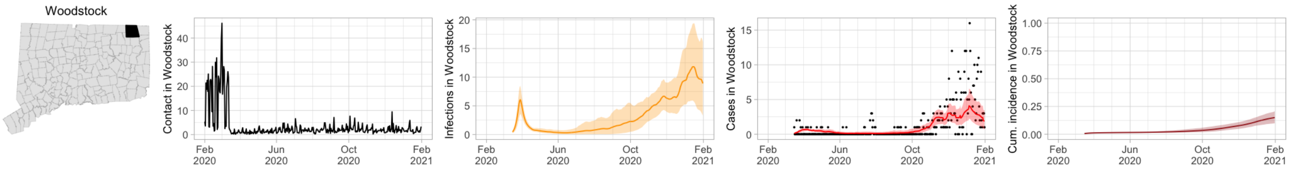

Figure 33: Contact for Connecticut towns and fitted SEIR model predictions with 95% uncertainty intervals.

References

[1] Glen Van Brummelen. *Heavenly mathematics: The forgotten art of spherical trigonometry*. Princeton University Press, 2012.

[2] Change to the list of reportable diseases, emergency illnesses and health conditions and the list of reportable laboratory findings. *Connecticut Epidemiologist*, 2020. URL `https://portal.ct.gov/-/media/DPH/EEIP/CTEPI/Vol40_No2.pdf`.

[3] Centers for Disease Control and Prevention. Coronavirus disease 2019 (covid-19) 2020 interim case definition, approved august 5, 2020, 2020. URL `https://wwwn.cdc.gov/nndss/conditions/coronavirus-disease-2019-covid-19/case-definition/2020/08/05/`.

[4] Olga Morozova, Zehang Richard Li, and Forrest W Crawford. A model for COVID-19 transmission in Connecticut. *medRxiv*, 2021. doi: https://doi.org/10.1101/2020.06.12.20126391. URL `https://www.medrxiv.org/content/10.1101/2020.06.12.20126391v2`.

[5] R Core Team. *R: A Language and Environment for Statistical Computing*. R Foundation for Statistical Computing, Vienna, Austria, 2020. URL `https://www.R-project.org/`.

[6] Karline Soetaert, Thomas Petzoldt, and R. Woodrow Setzer. Solving differential equations in R: Package deSolve. *Journal of Statistical Software*, 33(9):1–25, 2010.

[7] Tegan K Boehmer, Jourdan DeVies, Elise Caruso, Katharina L van Santen, Shichao Tang, Carla L Black, Kathleen P Hartnett, Aaron Kite-Powell, Stephanie Dietz, Matthew Lozier, et al. Changing age distribution of the COVID-19 pandemic — United States, May–August 2020. *Morbidity and Mortality Weekly Report*, 69(39):1404, 2020.

[8] Daniel P Oran and Eric J Topol. Prevalence of asymptomatic SARS-CoV-2 infection: A narrative review. *Annals of Internal Medicine*, 2020.

[9] Diana Buitrago-Garcia, Dianne Egli-Gany, Michel J Counotte, Stefanie Hossmann, Hira Imeri, Aziz Mert Ipekci, Georgia Salanti, and Nicola Low. Occurrence and transmission potential of asymptomatic and presymptomatic SARS-CoV-2 infections: A living systematic review and meta-analysis. *PLoS medicine*,

medRxiv preprint doi: https://doi.org/10.1101/2021.03.10.21253282; this version posted March 12, 2021. The copyright holder for this preprint (which was not certified by peer review) is the author/funder, who has granted medRxiv a license to display the preprint in perpetuity.
It is made available under a CC-BY 4.0 International license .

17(9):e1003346, 2020.

[10] Centers for Disease Control and Prevention. COVID-19 Pandemic Planning Scenarios. https://www.cdc.gov/coronavirus/2019-ncov/hcp/planning-scenarios.html, 2021. Accessed: 2021-01-18.

[11] Stephen M Kissler, Christine Tedijanto, Edward Goldstein, Yonatan H Grad, and Marc Lipsitch. Projecting the transmission dynamics of SARS-CoV-2 through the postpandemic period. *Science*, 368(6493):860–868, 2020.

[12] Henrik Salje, Cécile Tran Kiem, Noémie Lefrancq, Noémie Courtejoie, Paolo Bosetti, Juliette Paireau, Alessio Andronico, Nathanaël Hozé, Jehanne Richet, Claire-Lise Dubost, Yann Le Strat, Justin Lessler, Daniel Levy Bruhl, Arnaud Fontanet, Lulla Opatowski, Pierre-Yves Boelle, and Simon Cauchemez. Estimating the burden of SARS-CoV-2 in France. *Science*, 369(6500):208–211, 2020.

[13] Robert Verity, Lucy C Okell, Ilaria Dorigatti, Peter Winskill, Charles Whittaker, Natsuko Imai, Gina Cuomo-Dannenburg, Hayley Thompson, Patrick GT Walker, Han Fu, Amy Dighe, Jamie T Griffin, Marc Baguelin, Sangeeta Bhatia, Anne Boonyasiri, Adhiratha andd Cori, Zulma Cucunubá, Rich FitzJohn, Katy Gaythorpe, Will Green, Arran Hamlet, Wes Hinsley, Daniel Laydon, Gemma Nedjati-Gilani, Steven Riley, Sabine van Elsland, Erik Volz, Haowei Wang, Yuanrong Wang, Xiaoyue Xi, Christl A Donnelly, Azra C Ghani, and Neil M Ferguson. Estimates of the severity of coronavirus disease 2019: a model-based analysis. *The Lancet Infectious Diseases*, 20(6):669–677, 2020.

[14] Ruiyun Li, Sen Pei, Bin Chen, Yimeng Song, Tao Zhang, Wan Yang, and Jeffrey Shaman. Substantial undocumented infection facilitates the rapid dissemination of novel coronavirus (SARS-CoV-2). *Science*, 368(6490):489–493, 2020.

[15] Alberto Aleta, David Martin-Corral, Ana Pastore y Piontti, Marco Ajelli, Maria Litvinova, Matteo Chinazzi, Natalie E Dean, M Elizabeth Halloran, Ira M Longini Jr, Stefano Merler, Alex Pentland, Alessandro Vespignani, Esteban Moro, and Yamir Moreno. Modelling the impact of testing, contact tracing and household quarantine on second waves of COVID-19. *Nature Human Behaviour*, 4(9):964–971, 2020.

[16] Andrew William Byrne, David McEvoy, Aine B Collins, Kevin Hunt, Miriam Casey, Ann Barber, Francis Butler, John Griffin, Elizabeth A Lane, Conor McAloon, Kirsty O'Brien, Patrick Wall, Kieran A Walsh, and Simon J More. Inferred duration of infectious period of SARS-CoV-2: rapid scoping review and analysis of available evidence for asymptomatic and symptomatic COVID-19 cases. *BMJ Open*, 10(8), 2020. ISSN 2044-6055. doi: 10.1136/bmjopen-2020-039856. URL https://bmjopen.bmj.com/content/10/8/e039856.

[17] Wycliffe E Wei, Zongbin Li, Calvin J Chiew, Sarah E Yong, Matthias P Toh, and Vernon J Lee. Presymp-

medRxiv preprint doi: https://doi.org/10.1101/2021.03.10.21253282; this version posted March 12, 2021. The copyright holder for this preprint (which was not certified by peer review) is the author/funder, who has granted medRxiv a license to display the preprint in perpetuity.
It is made available under a CC-BY 4.0 International license .

tomatic transmission of SARS-CoV-2 – Singapore, January 23–March 16, 2020. *Morbidity and Mortality Weekly Report*, 69(14):411, 2020.

[18] Matthew Biggerstaff, Benjamin J Cowling, Zulma M Cucunubá, Linh Dinh, Neil M Ferguson, Huizhi Gao, Verity Hill, Natsuko Imai, Michael A Johansson, Sarah Kada, et al. Early insights from statistical and mathematical modeling of key epidemiologic parameters of COVID-19. *Emerging infectious diseases*, 26 (11), 2020.

[19] Conor McAloon, Áine Collins, Kevin Hunt, Ann Barber, Andrew W Byrne, Francis Butler, Miriam Casey, John Griffin, Elizabeth Lane, David McEvoy, et al. Incubation period of COVID-19: a rapid systematic review and meta-analysis of observational research. *BMJ open*, 10(8):e039652, 2020.

[20] Stephen A Lauer, Kyra H Grantz, Qifang Bi, Forrest K Jones, Qulu Zheng, Hannah R Meredith, Andrew S Azman, Nicholas G Reich, and Justin Lessler. The incubation period of coronavirus disease 2019 (COVID-19) from publicly reported confirmed cases: estimation and application. *Annals of Internal Medicine*, 172 (9):577–582, 2020.

[21] Jantien A Backer, Don Klinkenberg, and Jacco Wallinga. Incubation period of 2019 novel coronavirus (2019-ncov) infections among travellers from wuhan, china, 20–28 january 2020. *Eurosurveillance*, 25(5), 2020.

[22] Qun Li, Xuhua Guan, Peng Wu, Xiaoye Wang, Lei Zhou, Yeqing Tong, Ruiqi Ren, Kathy S.M. Leung, Eric H.Y. Lau, Jessica Y. Wong, Xuesen Xing, Nijuan Xiang, Yang Wu, Chao Li, Qi Chen, Dan Li, Tian Liu, Jing Zhao, Man Liu, Wenxiao Tu, Chuding Chen, Lianmei Jin, Rui Yang, Qi Wang, Suhua Zhou, Rui Wang, Hui Liu, Yinbo Luo, Yuan Liu, Ge Shao, Huan Li, Zhongfa Tao, Yang Yang, Zhiqiang Deng, Boxi Liu, Zhitao Ma, Yanping Zhang, Guoqing Shi, Tommy T.Y. Lam, Joseph T. Wu, George F. Gao, Benjamin J. Cowling, Bo Yang, Gabriel M. Leung, and Zijian Feng. Early transmission dynamics in Wuhan, China, of novel coronavirus–infected pneumonia. *New England Journal of Medicine*, 382(13):1199–1207, 2020.

[23] Jing Qin, Chong You, Qiushi Lin, Taojun Hu, Shicheng Yu, and Xiao-Hua Zhou. Estimation of incubation period distribution of COVID-19 using disease onset forward time: A novel cross-sectional and forward follow-up study. *Science Advances*, 6(33), 2020. doi: 10.1126/sciadv.abc1202. URL `https://advances.sciencemag.org/content/6/33/eabc1202`.

[24] Muge Cevik, Matthew Tate, Ollie Lloyd, Alberto Enrico Maraolo, Jenna Schafers, and Antonia Ho. SARS-CoV-2, SARS-CoV, and MERS-CoV viral load dynamics, duration of viral shedding, and infectiousness: a systematic review and meta-analysis. *The Lancet Microbe*, 2020.

[25] Kieran A Walsh, Karen Jordan, Barbara Clyne, Daniela Rohde, Linda Drummond, Paula Byrne, Susan

medRxiv preprint doi: https://doi.org/10.1101/2021.03.10.21253282; this version posted March 12, 2021. The copyright holder for this preprint (which was not certified by peer review) is the author/funder, who has granted medRxiv a license to display the preprint in perpetuity.
It is made available under a CC-BY 4.0 International license .

Ahern, Paul G Carty, Kirsty K O'Brien, Eamon O'Murchu, et al. SARS-CoV-2 detection, viral load and infectivity over the course of an infection. *Journal of Infection*, 2020.

[26] Xueting Qiu, Ali Ihsan Nergiz, Alberto Enrico Maraolo, Isaac I. Bogoch, Nicola Low, and Muge Cevik. Differences in secondary attack rates based on symptom status of index case(s)– a living systematic review. *medRxiv*, 2021. doi: 10.1101/2020.09.01.20135194. URL https://www.medrxiv.org/content/early/2021/01/16/2020.09.01.20135194.

[27] Rongrong Yang, Xien Gui, and Yong Xiong. Comparison of clinical characteristics of patients with asymptomatic vs symptomatic coronavirus disease 2019 in Wuhan, China. *JAMA Network Open*, 3(5): e2010182–e2010182, 2020.

[28] Kieran A Walsh, Susan Spillane, Laura Comber, Karen Cardwell, Patricia Harrington, Jeff Connell, Conor Teljeur, Natasha Broderick, Cillian F de Gascun, Susan M Smith, et al. The duration of infectiousness of individuals infected with SARS-CoV-2. *Journal of Infection*, 2020.

[29] Roman Wölfel, Victor M. Corman, Wolfgang Guggemos, Michael Seilmaier, Sabine Zange, Marcel A. Müller, Daniela Niemeyer, Terry C. Jones, Patrick Vollmar, Camilla Rothe, Michael Hoelscher, Tobias Bleicker, Sebastian Brünink, Julia Schneider, Rosina Ehmann, Katrin Zwirglmaier, Christian Drosten, and Clemens Wendtner. Virological assessment of hospitalized patients with COVID-2019. *Nature*, 581(7809): 465–469, 2020.

[30] David Mc Evoy, Conor G McAloon, Aine B Collins, Kevin Hunt, Francis Butler, Andrew W Byrne, Miriam Casey, Ann Barber, John M Griffin, Elizabeth A Lane, et al. The relative infectiousness of asymptomatic SARS-CoV-2 infected persons compared with symptomatic individuals: A rapid scoping review. *medRxiv*, 2020. doi: https://doi.org/10.1101/2020.07.30.20165084. URL https://www.medrxiv.org/content/10.1101/2020.07.30.20165084v1.

[31] Daihai He, Shi Zhao, Qianying Lin, Zian Zhuang, Peihua Cao, Maggie H Wang, and Lin Yang. The relative transmissibility of asymptomatic COVID-19 infections among close contacts. *International Journal of Infectious Diseases*, 2020.

[32] Centers for Disease Control and Prevention. Discontinuation of isolation for persons with COVID-19 not in healthcare settings. interim guidance. https://www.cdc.gov/coronavirus/2019-ncov/hcp/disposition-in-home-patients.html, 2021. Accessed: 2021-01-18.

[33] Shikha Garg, Lindsay Kim, Michael Whitaker, Alissa O'Halloran, Charisse Cummings, Rachel Holstein, Mila Prill, Shua J Chai, Pam D Kirley, Nisha B Alden, Breanna Kawasaki, Kimberly Yousey-Hindes, Linda Niccolai, Evan J Anderson, Kyle P Openo, Andrew Weigel, Maya L Monroe, Patricia Ryan, Justin Hen-

medRxiv preprint doi: https://doi.org/10.1101/2021.03.10.21253282; this version posted March 12, 2021. The copyright holder for this preprint (which was not certified by peer review) is the author/funder, who has granted medRxiv a license to display the preprint in perpetuity.
It is made available under a CC-BY 4.0 International license .

derson, Sue Kim, Kathy Como-Sabetti, Ruth Lynfield, Daniel Sosin, Salina Torres, Alison Muse, Nancy M Bennett, Laurie Billing, Melissa Sutton, Nicole West, William Schaffner, H. Keipp Talbot, Aquino Clarissa, Andrea George, Alicia Budd, Lynnette Brammer, Gayle Langley, Aron J Hall, and Alicia Fry. Hospitalization rates and characteristics of patients hospitalized with laboratory-confirmed coronavirus disease 2019 – COVID-NET, 14 states, March 1–30, 2020. *MMWR. Morbidity and Mortality Weekly Report*, 69, 2020.

[34] Pablo N Perez-Guzman, Anna Daunt, Sujit Mukherjee, Peter Crook, Roberta Forlano, Mara D Kont, Alessandra Løchen, Michaela Vollmer, Paul Middleton, Rebekah Judge, , Chris Harlow, Anet Soubieres, Graham Cooke, Peter J White, Timothy B Hallett, Paul Aylin, Neil Ferguson, Katharina Hauck, Mark Thursz, and Shevanthi Nayagam. Clinical characteristics and predictors of outcomes of hospitalized patients with coronavirus disease 2019 in a multiethnic London national health service trust: a retrospective cohort study. *Clinical Infectious Diseases*, 2020.

[35] Connecticut Hospital Association, 2021. URL `https://cthosp.org/`.

[36] Connecticut State Department of Public Health. COVID-19 Data Resources, 2021. URL `https://data.ct.gov/stories/s/COVID-19-data/wa3g-tfvc/`.

[37] United States Census Bureau. *American Community Survey (ACS)*, 2020. URL `https://www.census.gov/programs-surveys/acs`.

[38] CHIMEData, 2020. URL `https://chimedata.org/`.

[39] Iain Murray, Ryan Adams, and David MacKay. Elliptical slice sampling. In *Proceedings of the thirteenth international conference on artificial intelligence and statistics*, pages 541–548, 2010.

[40] Brian G Leroux, Xingye Lei, and Norman Breslow. Estimation of disease rates in small areas: a new mixed model for spatial dependence. In *Statistical Models in Epidemiology, the Environment, and Clinical Trials*, pages 179–191. Springer, 2000.

[41] Sumio Watanabe. Asymptotic equivalence of Bayes cross validation and widely applicable information criterion in singular learning theory. *Journal of Machine Learning Research*, 11(Dec):3571–3594, 2010.

[42] Purushottam W Laud and Joseph G Ibrahim. Predictive model selection. *Journal of the Royal Statistical Society: Series B (Methodological)*, 57(1):247–262, 1995.

[43] Jonathan W Pillow and James G Scott. Fully Bayesian inference for neural models with negative-binomial spiking. In *NIPS*, volume 1, pages 1907–1915, 2012.

[44] John Geweke. *Evaluating the Accuracy of Sampling-based Approaches to the Calculation of Posterior Moments*, volume 196. Federal Reserve Bank of Minneapolis, Research Department Minneapolis, MN, USA, 1991.

medRxiv preprint doi: https://doi.org/10.1101/2021.03.10.21253282; this version posted March 12, 2021. The copyright holder for this preprint (which was not certified by peer review) is the author/funder, who has granted medRxiv a license to display the preprint in perpetuity.
It is made available under a CC-BY 4.0 International license .

[45] Connecticut Data Collaborative. *Data By Topic*, 2020. URL http://data.ctdata.org/data_by_topic#demographics.

[46] Winston Chang, Joe Cheng, JJ Allaire, Yihui Xie, and Jonathan McPherson. *shiny: Web Application Framework for R*, 2020. URL https://CRAN.R-project.org/package=shiny. R package version 1.4.0.2.

[47] Joe Cheng, Bhaskar Karambelkar, and Yihui Xie. *leaflet: Create Interactive Web Maps with the JavaScript 'Leaflet' Library*, 2019. URL https://CRAN.R-project.org/package=leaflet. R package version 2.0.3.

[48] Kyle Walker. *tigris: Load Census TIGER/Line Shapefiles*, 2020. URL https://CRAN.R-project.org/package=tigris. R package version 1.0.

[49] Edward Parker. COVID-19 tracker, 2020. URL https://shiny.rstudio.com/gallery/covid19-tracker.html.

[50] Edward Parker. COVID-19 interactive mapping tool, 2020. URL https://github.com/eparker12/nCoV_tracker.

[51] Google. COVID-19 Community Mobility Reports. https://www.google.com/covid19/mobility, 2020.

[52] Apple. Mobility Trends Reports. https://covid19.apple.com/mobility, 2020.

[53] Facebook. COVID-19 Interactive Map & Dashboard. https://covid-survey.dataforgood.fb.com/?region=WORLD, 2020.

[54] Descartes Labs. Changes in Mobility. https://www.descarteslabs.com/mobility/, 2020.

[55] Michael S Warren and Samuel W Skillman. Mobility changes in response to COVID-19. *arXiv preprint arXiv:2003.14228*, March 2020. URL https://arxiv.org/abs/2003.14228.

[56] Cuebiq. Mobility Insights. https://www.cuebiq.com/visitation-insights-covid19/, 2020.
